# Supplementary cranial description of the types of *Edmontosaurus regalis* (Ornithischia: Hadrosauridae), with comments on the phylogenetics and biogeography of Hadrosaurinae

**DOI:** 10.1371/journal.pone.0175253

**Published:** 2017-04-06

**Authors:** Hai Xing, Jordan C. Mallon, Margaret L. Currie

**Affiliations:** 1 Beijing Museum of Natural History, Beijing Academy of Science and Technology, Beijing, China; 2 Palaeobiology, Canadian Museum of Nature, Ottawa, Ontario, Canada; Perot Museum of Nature and Science, UNITED STATES

## Abstract

The cranial anatomy of the flat-skulled hadrosaurine *Edmontosaurus regalis* (Ornithischia: Hadrosauridae) is extensively described here, based on the holotype and paratype collected from the middle part of the Horseshoe Canyon Formation in southern Alberta. Focus is given to previously undocumented features of ontogenetic and phylogenetic importance. This description facilitates overall osteological comparisons between *E*. *regalis* and other hadrosaurids (especially *E*. *annectens*), and revises the diagnosis of *E*. *regalis*, to which a new autapomorphy (the dorsal half of the jugal anterior process bearing a sharp posterolateral projection into the orbit) is added. We consider the recently named *Ugrunaaluk kuukpikensis* from the upper Campanian/lower Maastrichtian of Alaska a *nomen dubium*, and conservatively regard the Alaskan material as belonging to *Edmontosaurus* sp.. A phylogenetic analysis of Hadrosauroidea using maximum parsimony further corroborates the sister-taxon relationship between *E*. *regalis* and *E*. *annectens*. In the strict consensus tree, *Hadrosaurus foulkii* occurs firmly within the clade comprising all non-lambeosaurine hadrosaurids, supporting the taxonomic scheme that divides Hadrosauridae into Hadrosaurinae and Lambeosaurinae. Within Edmontosaurini, *Kerberosaurus* is posited as the sister taxon to the clade of *Shantungosaurus* + *Edmontosaurus*. The biogeographic reconstruction of Hadrosaurinae in light of the time-calibrated cladogram and probability calculation of ancestral areas for all internal nodes reveals a significantly high probability for the North American origin of the clade. However, the Laramidia–Appalachia dispersals around the Santonian–Campanian boundary, inferred from the biogeographic scenario for the North American origin of Hadrosaurinae, are in conflict with currently accepted paleogeographic models. By contrast, the Asian origin of Hadrosaurinae with its relatively low probability resulting from the biogeographic analysis is worth seriously considering, despite the lack of fossil material from the Santonian and lower Campanian of Asia. Extra fossil collecting in appropriate geographic locations and stratigraphic intervals of Asia and Europe will help to clarify the biogeographic dynamics of hadrosaurine dinosaurs in the near future.

## Introduction

Hadrosauridae is a derived group of ornithopod dinosaurs, currently known from the Upper Cretaceous (Santonian–Maastrichtian) of Eurasia and America [1–3]. The abundance and nearly cosmopolitan distribution of hadrosaurids implies that they were very successful, large-bodied herbivores during the closing stages of Cretaceous [4]. Hadrosauridae is phylogenetically defined as the least inclusive taxon containing *Saurolophus* and *Parasaurolophus* [5]. It is traditionally divided into two clades: the flat-skulled or solid-crested Hadrosaurinae and the hollow-crested Lambeosaurinae, based on the variable morphology of the facial skeleton, notably that of the paired premaxillae and nasals [1, 6].

**Fig 1:**
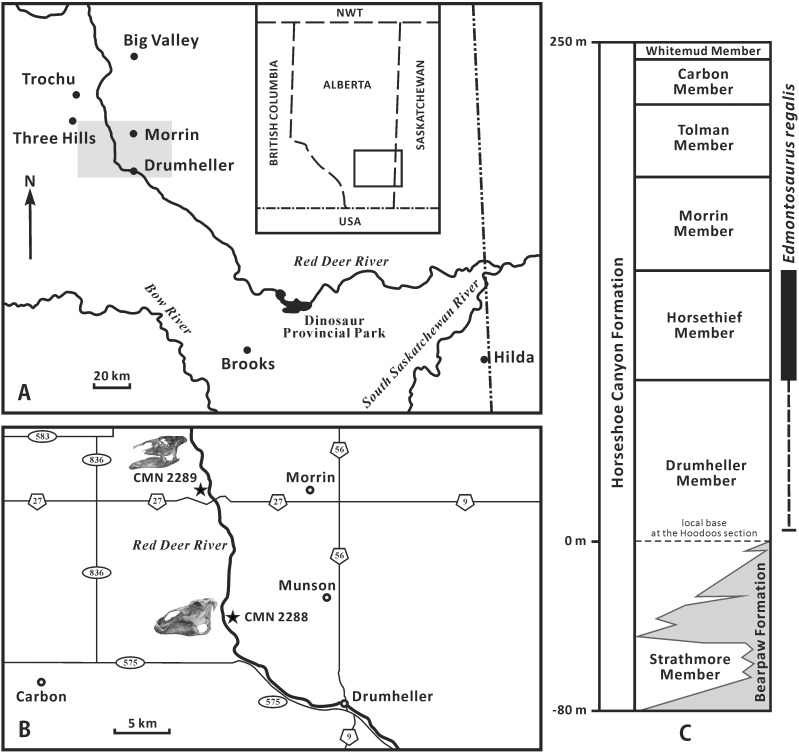
Geographic and stratigraphic distribution of *Edmontosaurus regalis* in Alberta (modified from [8, 17]). A, map of southern Alberta; the grey rectangle highlights the central region of the Red Deer River valley, where *E*. *regalis* specimens were collected. B, magnified map of the grey rectangular region in Fig 1A, showing the localities of the holotype (CMN 2288) and paratype (CMN 2289) of *E*. *regalis*. C, stratigraphic subdivision of the Horseshoe Canyon Formation, with the stratigraphic range of *E*. *regalis*.

*Edmontosaurus regalis* (the type species of the genus *Edmontosaurus*) has been recognized as a well-sampled flat-skulled hadrosaurine from North America [7, 8]. This species has proved integral to understanding patterns of ornithischian diversity [8], morphological variation of specific structures (such as the jaw muscles and circumnarial depression) in dinosaurs [9, 10], and histological growth dynamics of hadrosaurids [11]. *Edmontosaurus regalis* was named by Lambe [12] on the basis of two incomplete, partially articulated skeletons, namely the holotype (CMN 2288) and paratype (CMN 2289). Since the beginning of the 20th century, a large number of *E*. *regalis* specimens including the holotype and paratype, together with multiple bonebeds, were documented in the uppermost Campanian Horsethief Member of the Horseshoe Canyon Formation, along the central region of the Red Deer River valley in southern Alberta [13–15] (Fig 1). CMN 2288 was found by Levi Sternberg in 1912, along the east bank of the Red Deer River, opposite to the mouth of the Three Hills Creek (Fig 1B). The holotype quarry lies about 60 m above the river level, between the number 8 and number 9 coal seams. Subsequently, George F. Sternberg discovered CMN 2289 in 1916 along the west bank of the Red Deer River, 11 km west of the town of Morrin (Fig 1B). CMN 2289 occurs just below the number 9 coal seam, approximately 30 m above the river level [16, 17]. Bonebed material attributable to *E*. *regalis* has also recently been reported from the Horsethief Member near Edmonton, Alberta [18]. Given that a few *Edmontosaurus*-like remains have been identified just below the Horsethief Member, the stratigraphic range of *E*. *regalis* may extend down into the upper half of the Drumheller Member [17]. A coeval skull and partial vertebral column recently described from the Wapiti Formation near Grade Prairie, Alberta [19] supports this interpretation. Numerous disarticulated juvenile bones, possibly attributable to *E*. *regalis* (see Discussion), are also known from the Liscomb bonebed in the upper part of the Prince Creek Formation along the northwestern bank of the lower Colville River of northern Alaska [10, 20]. Radiometric dating constrains the narrow stratigraphic interval that encompasses this bonebed to the late Campanian–early Maastrichtian [21, 22]. The Liscomb quarry has proved difficult to precisely date because some studies provide inconsistent age estimates (late Campanian versus early Maastrichtian) for the bounding strata [20].

Lambe [16] described the cranial anatomy of *Edmontosaurus regalis* in detail, based on the holotype and paratype. His paper has greatly facilitated subsequent osteological studies of North American hadrosaurids. However, some anatomically and phylogenetically significant information for *E*. *regalis* was not mentioned or emphasized in Lambe’s paper, largely due to historical shortsightedness. Here, 100 years after the initial description of the species, we provide an important supplement to the cranial morphology of the types of *E*. *regalis*, in order to systematically revise this taxon and make a more detailed assessment of the phylogenetics and biogeography of Hadrosaurinae.

Institutional abbreviations: AENM, Amur Natural History Museum, RAS, Blagoveschensk, Russia; AMNH, American Museum of Natural History, New York, USA; BYU, Brigham Young University, Provo, USA; CCM, Carter County Museum, Ekalaka, USA; CM, Carnegie Museum of Natural History, Pittsburgh, USA; CMN, Canadian Museum of Nature, Ottawa, Canada; FMNH, The Field Museum, Chicago, USA; GMV, Geological Museum of China, Beijing, China; IVPP, Institute of Vertebrate Paleontology and Paleoanthropology, CAS, Beijing, China; MOR, Museum of the Rockies, Bozeman, USA; NHM, Natural History Museum, London, United Kingdom; NMMNH, New Mexico Museum of Natural History and Science, Albuquerque, USA; OTM, Old Trail Museum, Choteau, USA; PIN, Paleontological Institute of the Russian Academy of Sciences, Moscow, Russia; RAM, Raymond M. Alf Museum, Claremont, USA; ROM, Royal Ontario Museum, Toronto, Canada; SDSM, South Dakota School of Mines and Technology, Rapid City, USA; TMP, Royal Tyrrell Museum of Palaeontology, Drumheller, Canada; UALVP, University of Alberta Laboratory of Vertebrate Paleontology, Edmonton, Canada; UAM, University of Alaska Museum, Fairbanks, USA; USNM, United States National Museum, Smithsonian Institution, Washington, DC, USA; YPM, Yale Peabody Museum of Natural History, New Haven, USA; ZCDM, Zhucheng Dinosaur Museum, Zhucheng, China.

Anatomical abbreviations: aa, anterior apex of jugal; adp, anterodorsal process; af, alveolar foramina; alc, anterolateral concavity of premaxilla; alp, anterolateral process of palatine; amc, anteromedial concavity of premaxilla; an, angular; ap, anterior process; ar, anterior ramus; arp, alar process of basisphenoid; art, articular; avf, anteroventral flange of nasal; avp, anteroventral process of maxilla; bo, basioccipital; bpp, basipterygoid process of basisphenoid; bs, buccal shelf of dentary; bsp, basisphenoid–parasphenoid complex; co, crista otosphenoidalis of prootic; cp, coronoid process of dentary; cs, contact surface for designated bone; d, dentary; df, dorsal flange of pterygoid; dmf, dorsomedial flange of maxilla; dqp, dorsal quadrate process of pterygoid; dr, dorsal ramus of maxilla; edm, edentulous dorsal margin of dentary; en, external naris; ep, ectopterygoid; epr, ectopterygoid ridge of maxilla; eps, ectopterygoid shelf of maxilla; er, ectopterygoid ramus of pterygoid; es, enameled surface of tooth crown; ex, exoccipital–opisthotic complex; f, frontal; fm, foramen magnum; fo, fenestra ovalis; iaf, internal antorbital fenestra; ica, foramen for internal carotid artery; itf, infratemporal fenestra; j, jugal; jp, jugal process of lacrimal; jv, exit for jugular vein; jw, jugal wing of quadrate; l, lacrimal; laf, large anterior foramen of maxilla; lc, lateral condyle of quadrate; ldf, laterodorsal flange of surangular; lr, lateral ridge of nasal; lsp, laterosphenoid; m, maxilla; maf, mandibular adductor fossa; mc, medial condyle of quadrate; mes, medial shelf of surangular; mg, Meckelian groove of dentary; mp, medial process of pterygoid; mpr, median primary ridge of tooth crown; ms, metotic strut of exoccipital; mx, matrix; n, nasal; nc, neurocranium; om, oral margin of premaxilla; or, orbit; os, occlusal surface; osp, orbitosphenoid–presphenoid complex; p, parietal; pd, predentary; pdb, posterodorsal buttress of quadrate; pdf, posterodorsal flange of ectopterygoid; pdp, posterodorsal process; pf, posterior foramen of lacrimal; pl, palatine; plp, palatine process of maxilla; pls, posterolateral spur of quadrate; pm, premaxilla; pmp, posteromedial process of nasal; po, postorbital; pop, postorbital process of jugal; pp, posterior plate of nasal; pr, palatine ramus of pterygoid; prf, prefrontal; pro, prootic; pt, pterygoid; ptp, pterygoid process of maxilla; pvf, posteroventral fossa of prefrontal; pvfl, posteroventral flange; pvp, posteroventral process of premaxilla; pw, pterygoid wing of quadrate; q, quadrate; qg, quadrate glenoid of surangular; qh, quadrate head; qj, quadratojugal; qjn, quadratojugal notch of jugal; qs, quadrate shaft; rp, retroarticular process of surangular; sa, surangular; snd, subnarial depression; so, supraoccipital; sp, splenial; sq, squamosal; stf, supratemporal fenestra; syp, symphysial process of dentary; tr, tooth row; v, vomer; vpm, vestibular promontory of premaxilla; vqp, ventral quadrate process of pterygoid.

## Results

### Systematic paleontology

Dinosauria Owen, 1842 [23]

Ornithischia Seeley, 1887 [24]

Ornithopoda Marsh, 1881 [25]

Iguanodontia Dollo, 1888 [26] sensu Sereno, 1998 [5]

Hadrosauroidea Sereno, 1986 [27] sensu Sereno, 1998 [5]

Hadrosauridae Cope, 1870 [28] sensu Sereno, 1998 [5]

Hadrosaurinae Lambe, 1918 [29] sensu Sereno, 1998 [5]

Edmontosaurini Brett-Surman, 1989 [30]

*Edmontosaurus* Lambe, 1917 [12]

*Edmontosaurus regalis* Lambe, 1917 [12]

#### Synonym

*Thespesius edmontoni* Gilmore [31]; *Anatosaurus edmontoni* Lull and Wright [7]

#### Holotype

CMN 2288, partial, articulated cranium with a nearly complete left half and an incomplete postcranial skeleton, including six articulated anterior cervical vertebrae, eleven dorsal vertebrae, five caudal vertebrae, rib fragments, the sacrum, right scapula, left humerus, right ischium, and right hind limb missing some phalanges, and fragments of the paired ilia and pubes.

#### Paratype

CMN 2289, incomplete, partially articulated cranium and nearly complete, largely disarticulated postcranium, including three cervical vertebrae, fifteen dorsal vertebrae, five caudal vertebrae, seven cervical ribs, numerous dorsal ribs, the paired scapulae, right coracoid, paired sternals, paired humeri, paired ulnae, right radius, left second metacarpal, unguals of left manual digit II and right manual digit III, left ilium, paired pubes, paired ischia, paired femur, paired femora, paired tibiae, paired fibulae, and right astragalus, right metatarsals II and III, and left metatarsal IV (described in Campione [14]).

#### Referred material

All other known *Edmontosaurus* specimens recovered from the middle portion of the Horseshoe Canyon Formation and Unit 4 of the Wapiti Formation. Important specimens include: AMNH 5254, partial cranium; CM 26259, complete cranium and partial postcranium; CMN 8399, nearly complete skeleton missing the middle and posterior caudal vertebrae; CMN 8744, incomplete cranium; FMNH P15004, complete cranium; NHM R8927, complete cranium and postcranium; ROM 658, partial cranium; ROM 801, partial cranium and postcranium; ROM 867, incomplete cranium and postcranium; UALVP 53722, partial cranium, several cervical and dorsal vertebrae, and skin impressions showing fleshy cranial crest; and USNM 12711, complete cranium (modified from Campione and Evans [8]).

#### Locality and horizon

Outcrop and subsurface along the Red Deer River around the Drumheller, Morrin and Three Hills areas, southern Alberta, Canada [13]; along the North Saskatchewan River near Edmonton, Alberta [18]; and along the Red Willow River near Grande Prairie, Alberta [19].

Most specimens were found along the north-south oriented central area of the Red Deer River, and come from the Horsethief Member of the Horseshoe Canyon Formation, and possibly the upper half of the underlying Drumheller Member, ranging in age from 72.5 to 71.0 Ma (latest Campanian) [8, 17] (Fig 1). The Danek bonebed material from the North Saskatchewan River is restricted to the Horsethief Member [18]. The Red Willow River material is from Unit 4 of the Wapiti Formation, and is time-contemporaneous with the Drumheller Member of the Horseshoe Canyon Formation [19].

#### Revised diagnosis

Hadrosaurine of the genus *Edmontosaurus* characterized by the following three autapomorphies, most readily visible in large, presumably adult individuals: tapered ventral expansion of the subrectangular anterior end of the nasal; dorsal half of the jugal anterior process bearing a sharp posterolateral projection into the orbit; and mediolaterally wide, nearly horizontal shelf along the dorsal-facing base of the postorbital sutural surface of the jugal. Also diagnosed by the following unique combination of features: anteroposteriorly short prenarial region of the snout that is no more than 25% as long as the skull; postorbital strongly expanded laterally; deep fossa along the right-angled lateral half of the posteroventral side of the prefrontal; and truncated dorsolateral process of the laterosphenoid that is about 70% as long as the temporal plate of the bone. This taxon differs from *Edmontosaurus annectens* in having an anteroposteriorly short and dorsoventrally high cranium, a more swollen oral margin of the premaxilla that is posterodorsally curled and lip-like, two premaxillary foramina in the prenarial region of the circumnarial fossa separated by a relatively narrow vestibular promontory, a prominent arched posterodorsal margin of the circumnarial fossa ascending to the dorsal surface of the narial region of the snout, a slight insertion of the nasals into the frontals with a proportionally longer external interfrontal suture, a mediolaterally oriented, crenulated nasofrontal suture on the ectocranial surface, an anteroposteriorly wide, strongly bulgy laterally jugal process of the postorbital accompanied by a greater development of the postorbital pocket, and slightly mesiodistally narrower tooth crowns of the middle dentary having height/width ratios greater than 2.90 (modified from Campione and Evans [8]; Xing et al. [10]).

#### Remarks

In adult individuals of *Edmontosaurus regalis*, the well-defined posterodorsal margin of the strongly excavated circumnarial fossa ascends to the dorsal surface of the narial region of the snout, and markedly overhangs the dorsal edge of the external naris. Campione and Evans [8] first documented this feature, and regarded it as a diagnostic character of *E*. *regalis* that distinguishes the taxon from *E*. *annectens*. Nevertheless, Xing et al. [10] excluded this feature from the diagnosis of *E*. *regalis* because it seemed to be present in a few specimens of *E*. *annectens*, including the CCM unnumbered cranium. Our further observations indicate that the narial region in these specimens has suffered either distortion or artificial reconstruction. Therefore, the arcuate lateral ridge of the nasal ascending to the dorsal surface of the snout cannot be considered a natural character for *E*. *annectens*. In most cases, the arcuate lateral ridge of the nasal in *E*. *annectens* is positioned below the dorsal surface of the narial region of the snout in lateral view. We concur with Campione and Evans [8] that the prominent arched posterodorsal margin of the circumnarial fossa ascending to the dorsal surface of the narial region of the snout represents a diagnostic character of *E*. *regalis*, when compare to *E*. *annectens*. However, this feature is not autapomorphic for the taxon because the former is shared with *Shantungosaurus giganteus* (e.g. GMV 1780–2 and ZCDM HS0008; see Fig 4 in Xing et al. [10]).

### Osteological description and comparisons

**Fig 2:**
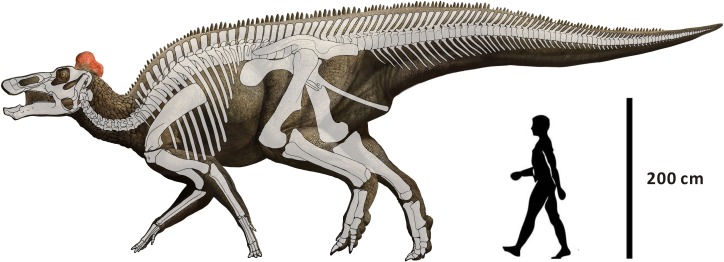
Reconstruction of *Edmontosaurus regalis* mainly based on CMN 2288, CMN 2289, CMN 8399, and UALVP 53722 (modified from Campione and Evans [8]).

The crania of the holotype and paratype of *Edmontosaurus regalis* are redescribed in this section, with the skeletal reconstruction of the species illustrated in Fig 2. Both CMN 2288 and CMN 2289 presumably represent the adult stage of *E*. *regalis* as indicated by their large size, the highly co-ossified braincase with closed neurocranial sutures, and the high number of alveoli in a single dentary. Here we emphasize important anatomical features not documented by Lambe [16], features of ontogenetic and phylogenetic significance, and remarkable osteological discrepancies among *E*. *regalis* and other hadrosaurids. Cranial measurements of the two specimens are listed in S1 Table.

#### Facial skeleton

**Fig 3:**
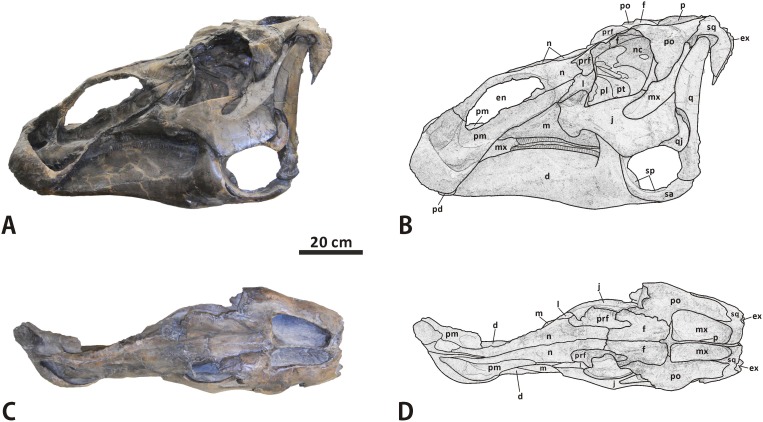
**Incomplete cranium of Edmontosaurus regalis (CMN 2288) in left lateral (A, B) and dorsal (C, D) views**; photographs (left) and line drawings (right).

**Fig 4:**
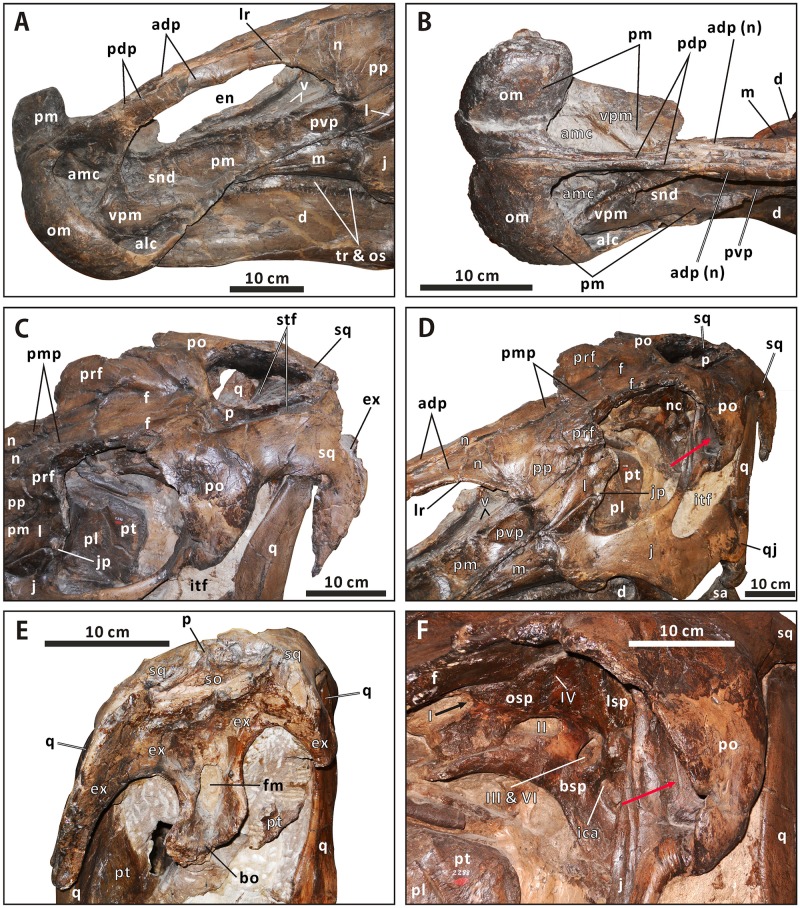
Close-ups of the cranium of *Edmontosaurus regalis* (CMN 2288). A, left laterodorsal view of the snout. B, anterodorsal view of the snout. C, left laterodorsal view of the skull roof. D, left anterolateral view of the posterior half of the skull. E, posterior view of the skull. F, left anterolateral view of the neurocranium through the orbit. The prominent postorbital pocket is indicated by a red arrow.

**Premaxilla** (Figs 3 and 4). The left premaxilla is well preserved in CMN 2288. This element consists of a laterally expanded oral region, a shortened, transversely thin posterodorsal process, and an elongate, sheet-like posteroventral process. The element bounds the external naris anteriorly and ventrally. The maximum width of the oral region is about 1.2 times greater than the minimum breadth of the posterior constriction. The outline of the oral margin is shallowly arched in dorsal view. The margin is strongly posterodorsally reflected, providing a thickened, lip-like anterior fence for the premaxilla. This is very similar to the condition in *Edmontosaurus annectens* (e.g. ROM 57100) and *Shantungosaurus giganteus* (GMV 1780–2), but is in striking contrast to the dorsoventrally flattened, slightly anteroventrally deflected oral margin in *Brachylophosaurus* [32], and the thin, gently upturned one in *Gryposaurus* [33]. In adults, the oral margin of the premaxilla in *E*. *annectens* is less swollen than that in *E*. *regalis* [8, 10]. Posterior to the oral margin, the circumnarial fossa in CMN 2288 has an anteriorly positioned, crescent-shaped outer narial fossa, as in other hadrosaurines [1]. A dorsolaterally-facing, subtriangular vestibular promontory divides the outer narial fossa into anteromedial and anterolateral concavities. The oval anteromedial concavity is slightly deeper and much wider than the teardrop-shaped anterolateral concavity. The prenarial region of the circumnarial fossa (the outer narial fossa) is proportionally shorter than that in *E*. *annectens* (e.g. AMNH 5730), with an anteroposteriorly narrower vestibular promontory.

The oral region extends and narrows posterodorsally to form the lateroventrally sloping posteroventral process. Immediately posterior to the vestibular promontory, the subnarial depression is well excavated and elongate. It occupies the anterior four fifths of the dorsolateral side of the posteroventral process. This depression forms part of the circumnarial fossa, which is posteriorly limited by an arcuate ridge, as in *Eotrachodon orientalis* [34]. The posteroventral process overlies the anteroventral process of the maxilla, and overlaps the anteroventral corner of the posterior plate of the nasal and anterodorsal portion of the lacrimal laterally. Unlike the condition in *Brachylophosaurus* (e.g. CMN 8893) and *Maiasaura* (e.g. ROM 44770), the posteroventral process does not reach the prefrontal. The posterodorsal process arises from the posteromedial part of the oral region, and contacts the anterodorsal process of the nasal laterally, along the anterior half of the dorsal edge of the external naris. Moving posteriorly, the process gradually thins transversely. It is about 40% as long as the posteroventral process. In CMN 2288, there is one premaxillary foramen at the anteriormost end of the subnarial depression and another in the posterior region of the anteromedial concavity. The palatal surface of the premaxilla does not have a rugose, shallowly arcuate lateroventral flange just anterior to the contact between the premaxilla and maxilla, which is observed in *Brachylophosaurus* and *Maiasaura*.

**Fig 5:**
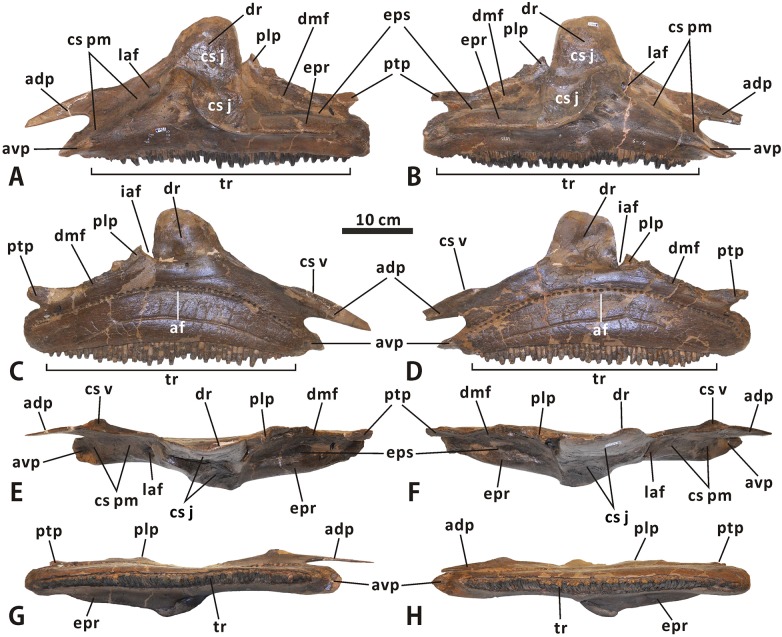
**Left and right maxillae of *Edmontosaurus regalis* (CMN 2289) in lateral (A, B), medial (C, D), dorsal (E, F), and ventral (G, H) views**.

**Fig 6:**
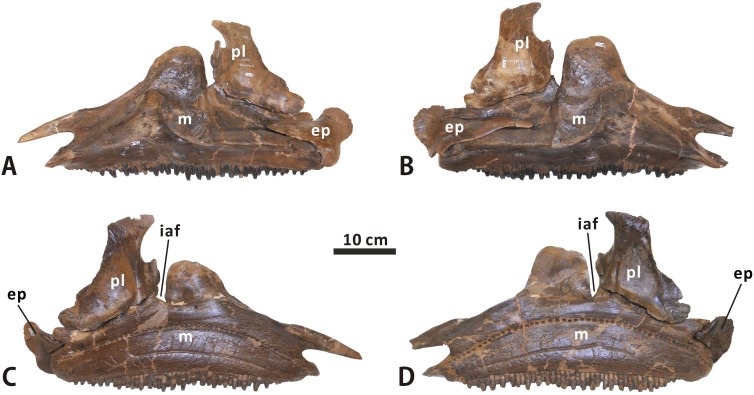
**Articulated left maxilla, palatine and ectopterygoid, and articulated right maxilla, palatine and ectopterygoid of *Edmontosaurus regalis* (CMN 2289) in lateral (A, B) and medial (C, D) views**.

**Maxilla** (Figs 3–6). The maxilla is roughly triangular in lateral view, with a nearly straight ventral edge and a tall dorsal ramus centered over the midline of the bone. In CMN 2289, the length of the maxilla at the level of the ventral edge is approximately 430 mm, which is ~160% greater than the maximum height of the element. The tooth row is partially exposed ventrally, and accounts for ~85% of the length of the maxillary ventral edge. Because the posterior end of the maxilla is laterally obscured by the dentary and jugal, the total number of tooth positions in CMN 2288 cannot be counted with precision.

Anteriorly, the maxilla is bifurcated into anterodorsal and anteroventral processes by a deep, posteriorly convex embayment. The anterodorsal process is anteroposteriorly elongate and mediolaterally flattened, as in other hadrosaurines. It gradually tapers anteriorly, but is entirely concealed laterally by the posteroventral process of the premaxilla when in articulation. This condition is also seen in *Gryposaurus notabilis* (e.g. ROM 873) and *Saurolophus angustirostris* (e.g. PIN 551/359). By contrast, the anterodorsal process of the maxilla is laterally visible through the external naris in articuated skulls of Brachylophosaurini [6]. In CMN 2289, the anterodorsal process projects dorsally from the dorsomedial corner of the anteroventral process. It is dorsally raised relative to the anteroventral process, and protrudes further anteriorly than the latter. In dorsal view, the medial side of the anterodorsal process bears a transversely narrow, triangular shelf for the reception of the vomer. The shelf is anteroposteriorly elongate, diminishing in width towards the dorsal ramus. The anterodorsal surface of the anteroventral process is modestly inclined ventrally and slightly concave. Its medial half, together with the lateral side of the anterodorsal process, forms the contact surface for the premaxilla, and is continuous posterodorsally with the sutural surface for the lacrimal along the dorsal margin of the dorsal ramus. A large, round anterior foramen on the anterodorsal surface of the anteroventral process is located half-way up the bone. This foramen is not covered by the premaxilla in CMN 2288.

Between the anterior foramen and the sutural surface for the jugal is a fully exposed, subtrapezoidal anterolateral promontory of the maxilla. The promontory is anteroposteriorly shortened along its dorsal half, compared to the equivalent in *Secernosaurus* and *Gryposaurus*. The dorsal ramus participates in the dorsal half of the jugal sutural surface. The apex of the dorsal ramus is bluntly round in lateral view, in contrast to the pointed dorsal extremity in all lambeosaurines, such as *Parasaurolophus tubicen* (e.g. NMMNH P-25100) and *Tsintaosaurus spinorhinus* (IVPP V725). The sutural surface for the jugal is irregularly diamond-shaped. Its ventral half faces laterodorsally, and bears a lateroventrally directed posterior margin as in all hadrosaurines except brachylophosaurins. The sharply defined anterior margin of the ventral half of the jugal sutural surface curves posteroventrally and laterally, and abuts the robust ectopterygoid ridge posteriorly. The ventral tip of the jugal sutural surface has a round eminence that is less developed than the equivalent in *Kundurosaurus* (e.g. AENM 2/84). Just anteroventral to the jugal sutural surface, the lateral surface of the maxilla is perforated by three large, ovate foramina that are anterodorsally-posteroventrally aligned.

The lip-shaped ectopterygoid ridge is straight and robust along its entire length. This ridge is located approximately one quarter of the way up the maxilla, where the bone is widest mediolaterally. A tall, gradually posteriorly descending flange projects dorsally from the medial surface of the maxillary posterior third. The subtriangular, dorsally directed palatine process and finger-shaped, posteriorly directed pterygoid process are located at the anterodorsal and posterodorsal corners of the dorsomedial flange, respectively. Lateral to the base of the flange, a shallow, transversely narrow groove occurs along the dorsal side of the ectopterygoid shelf. In medial view, the prominent notch between the dorsal ramus and palatine process forms part of the internal antorbital fenestra. A dorsally convex row of alveolar foramina arches across the medial surface of the maxilla at mid-height, as in other hadrosaurids.

**Fig 7:**
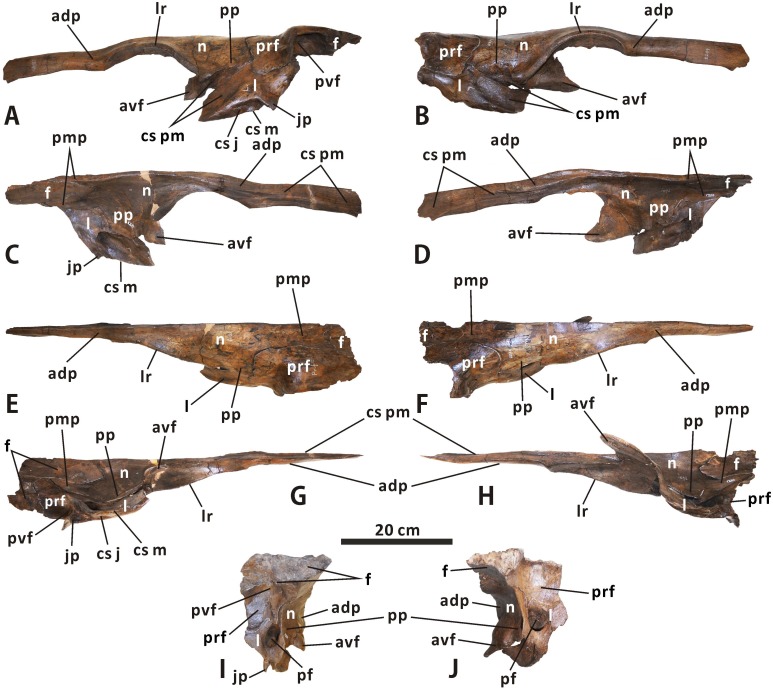
**Articulated left nasal, prefrontal, lacrimal and frontal, and articulated right nasal, prefrontal, lacrimal and frontal of *Edmontosaurus regalis* (CMN 2289) in lateral (A, B), medial (C, D), dorsal (E, F), ventral (G, H), and posterior (I, J) views**.

**Nasal** (Figs 3, 4 and 7). As in *Acristavus gagslarsoni* (MOR 1155), *Kundurosaurus nagornyi* (e.g. AENM 2/57), and *Edmontosaurus annectens* (e.g. USNM 3814), the nasal is hatchet-shaped in lateral view. It consists of an anteroposteriorly elongate anterodorsal process, a large, trapezoidal posterior plate that is laterodorsally convex, and a dorsoventrally thin, subrectangular posteromedial process. Except for the anterior half of the anterodorsal process, the nasal meets its counterpart along the smooth posterior three quarters of the dorsoventrally narrow dorsomedial surface. The anterodorsal process delimits the external naris dorsally. The anterior half of the process is nearly straight, mediolaterally compressed, and subrectangular. It flanks the posterodorsal process of the premaxilla laterally, and terminates at the anterior end of the external naris. This is distinct from the anteriorly pointed process in *Gryposaurus* (e.g. CMN 2278), *Kritosaurus* (BYU 12950), and *Maiasaura* (e.g. ROM 44770). The anteroventral corner of the anterodorsal process in CMN 2288 exhibits a tapered, slightly flared ventral expansion that is absent in *E*. *annectens* [8]. The lateroventral margin of the anterodorsal process abuts the vestibular promontory of the premaxilla. In CMN 2289, the dorsomedial surface of the anterior half of the anterodorsal process has a long, posteriorly tapered sutural surface for the premaxilla, which is ornamented with numerous longitudinal striations. The posterior half of the anterodorsal process gradually widens and deepens posteriorly along its dorsal region, to partially shroud the posterodorsal region of the circumnarial fossa, where the lateroventral margin of the anterodorsal process bifurcates posteriorly into a prominent, arched lateral ridge and the more medially positioned dorsal edge of the external naris. The thick lateral ridge is dorsally convex, extending posteroventrally to the lateral side of the posterior plate. This ridge forms the posterodorsal margin of the strongly excavated circumnarial fossa, the roof of which ascends to the dorsal surface of the narial region of the snout. By contrast, the position of the lateral ridge of the nasal is relatively low in *Kerberosaurus* (AENM 1/318), *E*. *annectens* (e.g. YPM 2182), and juvenile *E*. *regalis* (e.g. CM 26259). The posteriormost end of the lateral ridge in *E*. *regalis* forms a subtriangular protuberance, and is much thicker than the rest of the ridge.

A triangular flange projects anteroventrally from the anteroventral corner of the medial surface of the posterior plate. The dorsal margin of the flange defines the external naris posteroventrally, and is continuous with the lateroventral margin of the anterodorsal process. Posterior to the anteroventral flange, the main body of the posterior plate contacts the lacrimal posteroventrally, and is laterally overlapped by the anteromedial part of the prefrontal along its posterior terminus. The lateral side of the articulated nasal and lacrimal is slightly recessed to form a posterodorsally directed, finger-shaped contact surface for the premaxilla. Posterior to the posteroventral process of the premaxilla, a small part of the nasal-lacrimal contact is visible laterally in the articulated cranium of CMN 2288. The dorsal surface of the posterior plate is essentially flat. It does not bear the preorbital nasal protuberance in *Gryposaurus* and *Rhinorex* [35, 36]. The posteromedial process of the nasal laps onto the anterior platform of the frontal, and meets the prefrontal laterally. The ratio of the process length relative to the total length of the nasal is about 0.24, and the process is slightly shorter than that in *Edmontosaurus annectens*, where the equivalent ratio is over 0.29. In dorsal view, the dorsal nasofrontal suture is mediolaterally oriented and crenulated. It defines two small, slightly divergent processes of the paired nasals adjacent to the sagittal plane, which extend posteriorly onto the middle regions of the paired frontals. The external suture between the nasal and prefrontal approximates a right angle; it runs dorsoventrally and anteroposteriorly along the laterodorsal surface of the facial skeleton between the narial and orbital regions.

**Prefrontal** (Figs 3, 4 and 7). The prefrontal contacts the nasal anteromedially, the lacrimal ventrally, and the frontal posteriorly. The nearly square preorbital region of the prefrontal is mediolaterally thin and slightly convex laterodorsally. This region is anteroposteriorly elongate, occupying the anterior third of the bone, as in *Edmontosaurus annectens*. In contrast, the equivalent structure is extremely shortened in *Saurolophus* (e.g. AMNH 5220) and *Prosaurolophus* (e.g. ROM 1928). The posterior two thirds of the prefrontal in CMN 2288 and CMN 2289 broadens transversely to reach 2 times the maximum width of the anterior third, where it contributes to the anterodorsal part of the orbital margin. The lateral half of the orbital region of the prefrontal is right-angled in lateral view, in contrast to the arcuate lateral profile in Brachylophosaurini and basal hadrosauroids. Its posteroventral surface bears a deep, circular fossa, as in *E*. *annectens*. In dorsal view, the prefrontal posteriorly wedges into the anterolateral part of the frontal. The dorsal prefrontal-frontal suture is more posteriorly positioned than the ventral one. Ventrally, the prefrontal overlaps the lacrimal dorsolaterally, with a roughly W-shaped posterior suture and a U-shaped lateral suture between the two bones.

**Lacrimal** (Figs 3, 4 and 7). The lacrimal is well preserved in CMN 2289, especially the left one, where its contacts with the neighboring bones are clearly visible. This bone is subtrapezoidal in lateral view, with a transversely thickened posterior third. A low, subtriangular eminence is present in the centre of the lateral side of the lacrimal. The eminence tapers anteroventrally between the sutural sufaces for the premaxilla and jugal. There is a deep, anteroventrally-posterodorsally oriented trough on the medial surface of the anterior two thirds of the lacrimal. This trough passes posterodorsally through the rest of the bone, and opens into the oval posterior foramen that is common among hadrosauroids [32, 37]. The striated surface around the trough indicates that the trough was medially covered by the dorsal ramus of the maxilla. The posterior foramen is dorsoventrally deeper than mediolaterally wide.

In posterior view, the lacrimal is bounded by the sharp posterolateral and posteromedial margins. Lateral to the posterior foramen, the dorsal half of the posterolateral region of the lacrimal curves and thins laterally to form a fan-shaped flange along the anterior orbital margin, where the element reaches its greatest mediolateral width. A thin, subrectangular sheet projects dorsally from the posterior half of the dorsomedial part of the lacrimal. The sheet meets the prefrontal laterally and the nasal dorsomedially, and is about 40% as deep as the entire lacrimal. Medially, the lacrimal contacts the nasal anterodorsally along an anteroventrally-posterodorsally oriented, roughly W-shaped suture. The sutural surface for the jugal occurs along the entire ventral side of the lacrimal, except for the smooth anteriormost end and rugose medial edge of the latter that would contact the maxilla. The jugal surface is transversely narrow, lateroventrally facing and anteriorly tapering, with an obtusely angled lateral margin that opens ventrally. A stout, triangular jugal process is present at the posteroventral corner of the lateral surface of the bone, as in other hadrosaurines.

**Fig 8:**
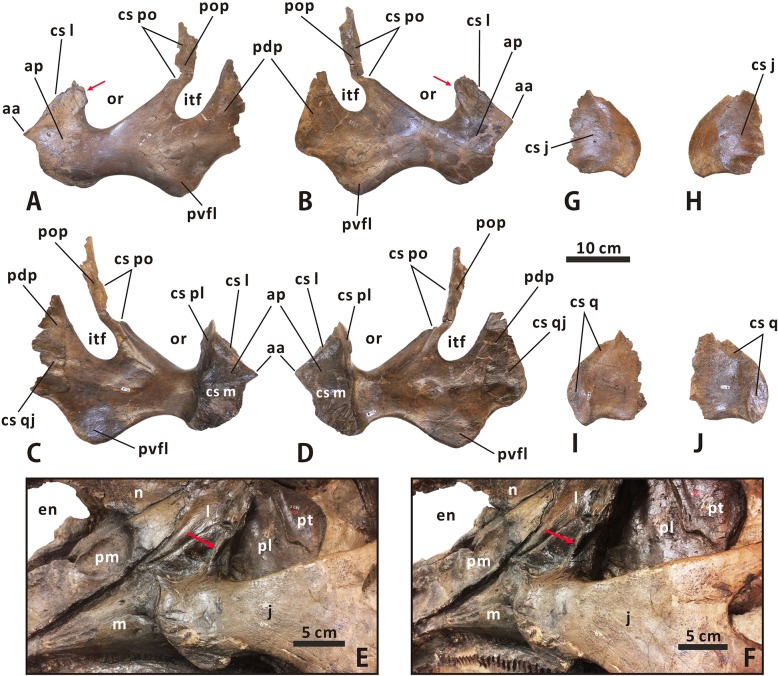
Left and right jugals of *Edmontosaurus regalis* (CMN 2289) in lateral (A, B) and medial (C, D) views. Close-ups of the jugal anterior process of CMN 2288 in lateral (E) and posterolateral (F) views. Left and right quadratojugals of *Edmontosaurus regalis* (CMN 2289) in lateral (G, H) and medial (I, J) views. The sharp posterolateral projection of the jugal along the orbital margin is indicated by an arrow.

**Jugal** (Figs 3, 4 and 8). As in other hadrosauroids, the jugal is bowed laterally and mediolaterally compressed between its contacts with the maxilla anteriorly and quadratojugal posteriorly [38, 39]. This triradiate bone has a dorsoventrally expanded, asymmetrically spade-shaped anterior process that tapers anteriorly to a stout triangular apex. In CMN 2288, the maxilla-lacrimal contact is laterally covered by the jugal. The anterior apex occurs within the dorsal half of the anterior process as in *Gryposaurus* (e.g. MOR 478-6-10-87-2), *Kundurosaurus* (e.g. AENM 2/921-2), and *Prosaurolophus* (e.g. CMN 2870), but is slightly shorter than that of these taxa. By contrast, the extremely elongate anterior apex is located at the mid-height of the subtriangular anterior process of the jugal in *Acristavus*, *Maiasaura*, and *Brachylophosaurus*. Posterior to the apex, the anterodorsal surface of the anterior process in both CMN 2288 and CMN 2289 is modestly anteroventrally inclined and transversely narrow, forming an angle of 40° with the horizontal. The surface is gently excavated to receive the lacrimal. The posterodorsal part of the anterior process broadens mediolaterally and deepens dorsoventrally, where it is remarkably flared posterolaterally to form a sharp projection that invades the orbital margin. In lateral view, the convex ventral edge of the anterior process is shallowly arcuate, and is comparable to that in Saurolophini. Medially, the rugose contact surface for the maxilla is posteriorly delineated by the prominent, dorsoventrally oriented, weakly wavy posteromedial margin of the anterior process. The dorsal half of the margin is formed by the narrow, strip-like palatine sutural surface that contrasts with the relatively wide, lunate equivalent in many lambeosaurines, such as *Amurosaurus riabinini* [40].

The postorbital process projects posterodorsally from the middle of the jugal, and is slightly tilted anteriorly along its dorsal half where the elongate contact surface for the postorbital occurs. The process has a bend roughly midway, with an angle of 150°. The dorsal half of the postorbital process is more mediolaterally compressed than the ventral half. There is a mediolaterally wide, nearly horizontal shelf at the dorsal-facing base of the postorbital contact surface [8]. The posterior neck below the infratemporal fenestra is more robust than that in all other hadrosauroids except *Edmontosaurus annectens*. In CMN 2289, the ratio between the depths of the posterior and anterior necks is ~1.50. Very similar to the condition in *Gryposaurus notabilis* (e.g. TMP 80.22.1) and *Probrachylophosaurus bergei* (e.g. MOR 2919), the posteroventral flange is strongly convex ventrally. It is more developed than the equivalent in *Prosaurolophus maximus* (e.g. USNM 12712) and *Saurolophus angustirostris* (e.g. PIN 551/357). The posterodorsal process is dorsoventrally deep and subquadrangular, in contrast to the relatively narrow, fan-shaped posterodorsal process in Brachylophosaurini [41]. The dorsal margin of the process is more dorsally deflected than the ventral margin. In medial view, the middle region of the jugal bears a large, suboval pit that probably functioned in muscle attachments.

**Quadratojugal** (Figs 3, 4 and 8). The quadratojugal is a thin, plate-like element that is sandwiched between the jugal and quadrate. The paired quadratojugals in CMN 2289 are nearly complete, and are missing their anteriormost parts. Judging from the medial outline of the quadratojugal sutural surface of the jugal, the quadratojugal appears to have a nearly vertical anterior edge and a roughly right-angled anteroventral corner. The lateral side of the bone is slightly anteroposteriorly convex, while the medial side is generally flat. The posteroventral region of the quadratojugal slightly projects ventrally to form a short triangular flange, in striking contrast to the elongate hook-like flange in *Hypacrosaurus altispinus*.

The anterior three fifths of the lateral side of the quadratojugal forms a rugose sutural facet for the jugal posterodorsal process. This facet is posteriorly bounded by a faint, deeply arched ridge. The rest of the lateral side is relatively smooth, and is externally exposed in articulated crania such as CMN 2288. In medial view, a shallow, anteroposteriorly narrow, crescent-shaped depression occurs along the arcuate posterior margin of the quadratojugal to accommodate the entire quadratojugal notch of the quadrate, suggesting that there was no gap between the quadratojugal and quadrate when in articulation.

**Fig 9:**
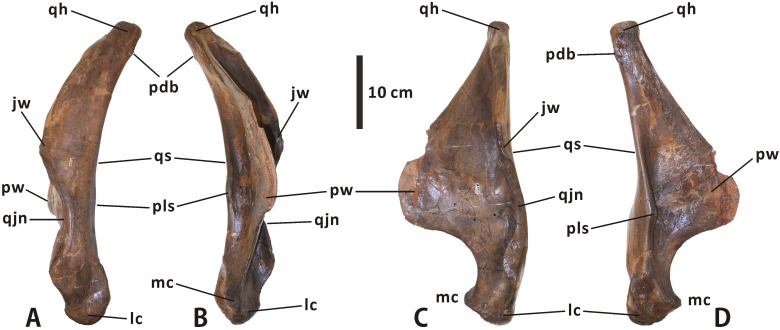
**Left quadrate of *Edmontosaurus regalis* (CMN 2289) in lateral (A), medial (B), anterior (C), and posterior (D) views**.

**Quadrate** (Figs 3, 4 and 9). The quadrate is dorsoventrally elongate and robust. It curves slightly posteriorly along its dorsal half, with a deflection angle of 155 degrees. The quadrate head is slightly anteroposteriorly longer than mediolaterally wide, and has a roughened, gently convex dorsal surface with an oval outline. A weakly developed buttress is found in the posterolateral region of the quadrate head, as in *Shantungosaurus* (e.g. ZCDM HS0031) and *Prosaurolophus* (e.g. ROM 1928). This condition strongly contrasts with the pronounced posterdorsal process of the quadrate in *Probrachylophosaurus* and *Gryposaurus* [33, 42]. The ventral half of the anteriorly projecting lateral flange of the quadrate shaft is slightly notched to receive the quadratojugal. The quadratojugal notch is symmetrical and shallowly arcuate, with a progressively descending anteroventral edge, closely resembling the condition in *Acristavus*, *Kundurosaurus*, and *Eotrachodon* but differing from the deep, asymmetrical notch bearing an anteriorly directed ventral edge in *Aralosaurus* and *Prosaurolophus*. In lateral view, the contact surface of the notch is narrow and slightly sunken, and faces anterolaterally. The thin, striated jugal wing occurs immediately dorsal to the quadratojugal notch.

The pterygoid wing arises from the medial surface of the quadrate shaft, and projects anteromedially. The wing is anteroposteriorly flattened and tongue-shaped in anterior outline. In dorsal view, it forms an angle of 70° with the jugal wing. The flat anterior surface of the pterygoid wing might act as the attachment area for M. adductor mandibulae posterior, as noted by Holliday [9]. As in *Velafrons coahuilensis* [43], a small spur protrudes medially from the posterolateral margin of the quadrate shaft, slightly below the mid-height of the bone. Between the posterolateral spur and the base of the pterygoid wing is a deep, longitudinal groove in the middle of the posterior side of the quadrate. Ventrally, the medial condyle is smaller and much more dorsally positioned than the lateral condyle. However, in *Probrachylophosaurus bergei* (e.g. MOR 2919), the medial condyle is slightly elevated relative to the lateral one.

**Fig 10:**
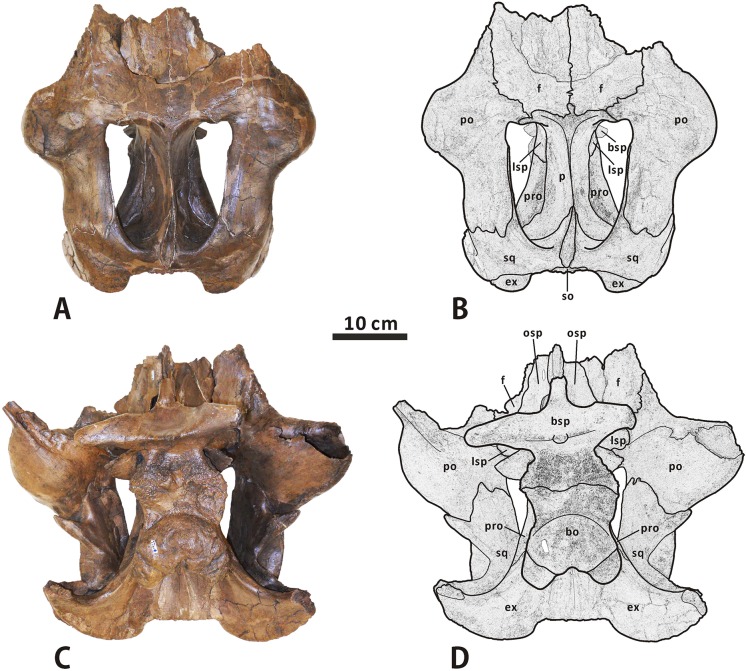
**Partial, articulated skull roof and neurocranium of *Edmontosaurus regalis* (CMN 2289) in dorsal (A, B) and ventral (C, D) views**; photographs (left) and line drawings (right).

**Fig 11:**
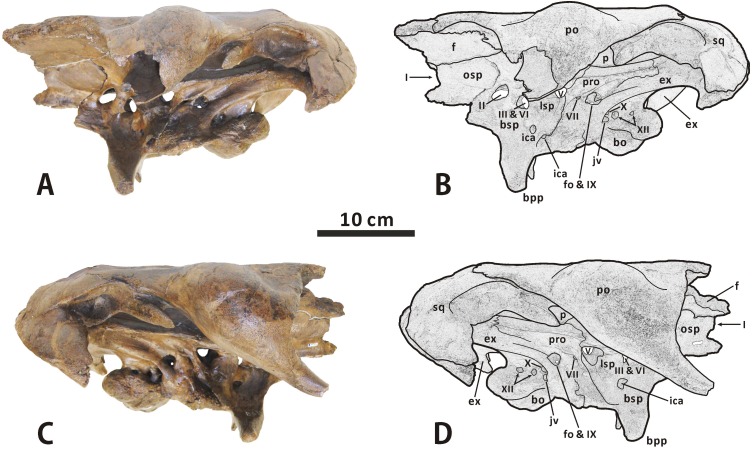
**Partial, articulated skull roof and neurocranium of *Edmontosaurus regalis* (CMN 2289) in left lateral (A, B) and right lateral (C, D) views**; photographs (left) and line drawings (right).

**Fig 12:**
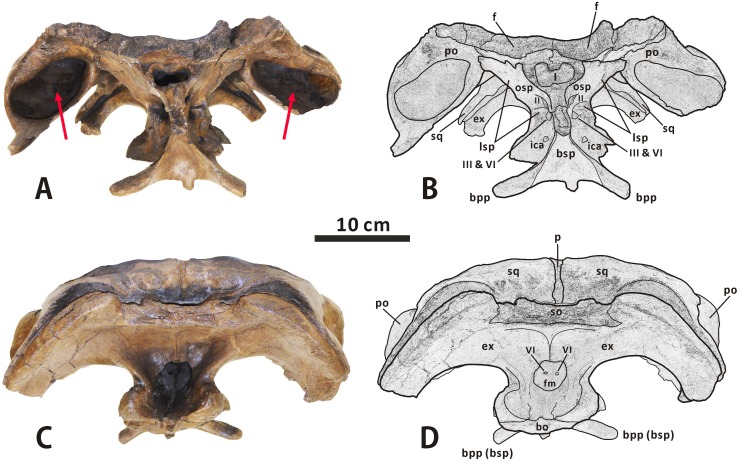
**Partial, articulated skull roof and neurocranium of *Edmontosaurus regalis* (CMN 2289) in anterior (A, B) and posterior (C, D) views**; photographs (left) and line drawings (right). The well-developed postorbital pocket is indicated by an arrow.

**Squamosal** (Figs 3, 4 and 10–12). The squamosal is a quadradiate, laterodorsally bowed bone that comprises the posterolateral corner of the supratemporal fenestra and the medioventral half of the intertemporal bar. As in other hadrosaurines, the central region of the squamosal is not drastically elevated relative to the neurocranium. The anterior half of the element is laterodorsally overlapped by the postorbital, where an extremely narrow band of the squamosal is dorsally exposed and forms part of the lateral margin of the supratemporal fenestra. In ventral view, a short, mediolaterally compressed process extends anteriorly from the central region of the squamosal, to meet the base of the jugal process of the postorbital. The quadrate cotylus is deep and suboval, and has a parasagittal long axis. This cotylus is slightly posteroventrally inclined, forming an angle of 20° with the dorsal side of the skull roof. Just below the postorbital-squamosal joint, there is a deep, strongly constricted precotyloid fossa that tapers posterodorsally and medially. The fossa limits the anteroventrally and slightly laterally directed, subconical precotyloid process anterodorsally. The precotyloid process is subtriangular in cross-section. It is proportionally shorter than that in *Prosaurolophus* (e.g. ROM 787), *Lophorhothon* (FMNH P27383), *Kritosaurus* (e.g. NMMNH P-16106), and *Gryposaurus* (e.g. AMNH 5350). The postcotyloid process is anteroposteriorly broad and anteroventrally curved. The process overlaps the ventral half of the exoccipital paroccipital process laterodorsally along its posterolateral region.

In posterior view, the lateral surface of the squamosal is moderately tilted dorsomedially. The central region of the bone contacts the dorsal half of the exoccipital paroccipital process along the deeply arcuate, sharply defined posterodorsal margin. The medial rami of the paired squamosals are separated by a posterior extension of the parietal sagittal crest, which is distinct from the connected squamosals seen in *Saurolophus* (e.g. AMNH 5221). Dorsally, the raised posterior margin of the supratemporal fenestra divides each ramus into a strongly concave anterodorsal surface and a slightly convex posterodorsal surface. The anteromedial region of the medial ramus extends and curves anteriorly, and shares a wedge-shaped suture with the parietal.

**Postorbital** (Figs 3, 4 and 10–12). The postorbital is composed of anteromedial, posterior, and jugal processes. The bone varies substantially from that in all other hadrosauroid taxa. The anteromedial surface of the jugal process is strongly concave posteriorly and expanded laterally to form a large and deep pocket [8, 16]. The pocket is subovate and lateroventrally-dorsomedially oriented in anterior view, and is approximately 68% as wide as the frontal across the orbital margin. By contrast, the postorbital pocket is relatively narrow mediolaterally and shallow anteroposteriorly in *Edmontosaurus annectens* (e.g. ROM 57100) and juvenile *E*. *regalis* (e.g. CMN 8744), where the width of the pocket is less than 50% the maximum width of the frontal. In both CMN 2288 and CMN 2289, the jugal process forms an equilateral triangle, and curves anteroventrally. The strong anteroposterior expansion of the process leads to an extreme constriction of the dorsal region of the infratemporal fenestra. However, the equivalent structure in juvenile *Edmontosaurus* specimens (e.g. UAM ES12965) is relatively slender, and is more comparable to that in most hadrosaurines, such as *Probrachylophosaurus* (MOR 2919) and *Saurolophus* (e.g. AMNH 5220). Through ontogeny, the jugal process of *E*. *regalis* becomes progressively larger relative to the skull roof.

The main body of the postorbital is dorsolaterally ornamented with a large, domed bump, which follows the great development of the postorbital pocket. The shortened anteromedial process abruptly widens to receive the frontal and parietal. The posterior process contacts the squamosal ventromedially along its entire length, and terminates just above the anterior half of the quadrate cotylus. In lateral view, the process is approximately level with the anteromedial process. This contrasts with *Kritosaurus navajovius* (e.g. USNM 8629), in which the posterior process of the postorbital is obliquely angled posteriorly relative to the anteromedial process and extends onto the anterolateral surface of the central squamosal immediately dorsal to the precotyloid fossa.

**Frontal** (Figs 3, 4 and 10–12). The frontal forms the anterior region of the skull roof, and is excluded from the supratemporal fenestra by the postorbital-parietal joint. The dorsal surface of the element is smooth and strongly depressed relative to the flat, rugose anterodorsal side of the postorbital, as in *Shantungosaurus giganteus* and *Edmontosaurus annectens* [10]. In dorsal view, the frontal contacts the postorbital along an interdigitated, posterolaterally convex suture. The anterior platform of the frontal for receiving the nasal is substantially shorter than that in *Brachylophosaurus* (e.g. MOR 1071-7-13-99-87-I) and *Probrachylophosaurus* (MOR 2919), and is less steeply angled than that in *Maiasaura* (e.g. OTM F138), *Saurolophus* (e.g. AMNH 5221), and *Prosaurolophus* (e.g. TMP 1981.23.140).

Ventrally, the paired frontals form the dorsal border of the anterior exit for the olfactory nerve (CN I) as they contact the narrow dorsal surfaces of the left and right orbitosphenoid–presphenoid complexes. The posterolateral part of the frontal meets the dorsolateral process of the laterosphenoid along a short scarf joint, whose ventral suture is continuous with the anteromedially-posterolaterally oriented lateral suture between the frontal and the orbitosphenoid. The endocranial surface of the frontal contributes to the anterior region of the shallow, subcircular cerebral fossa.

**Parietal** (Figs 3, 4 and 10–12). The single parietal consists of paired anterolateral and posterolateral processes, and an anteroposteriorly elongate main plate, with an hourglass-shaped dorsal outline. Between the slender anterolateral processes, there is a mediolaterally narrow, finger-shaped interfrontal process that arises from the main plate. The process is much more exposed endocranially than dorsally. Ventrally, the anterior third of the main plate forms the posterior cerebral fossa. As in other hadrosaurines, the sagittal crest of the main plate is straight and level with the posterior half of the skull roof, contrasting with the strongly down-warped sagittal crest in most lambeosaurines [44]. The crest remains narrow and sharp along its middle part, but slightly widens at the anterior and posterior ends of the main plate. The posterior third of the sagittal crest does not bear the bifurcated secondary structures observed in some basal hadrosauroids, such as *Levnesovia transoxiana* (USNM 538191). The parietal contacts the laterosphenoid, prootic, and supraoccipital ventrally along a medially and slightly ventrally embayed suture. The posteroventral part of the parietal is firmly upheld by the ascending process of the supraoccipital.

#### Palate

**Fig 13:**
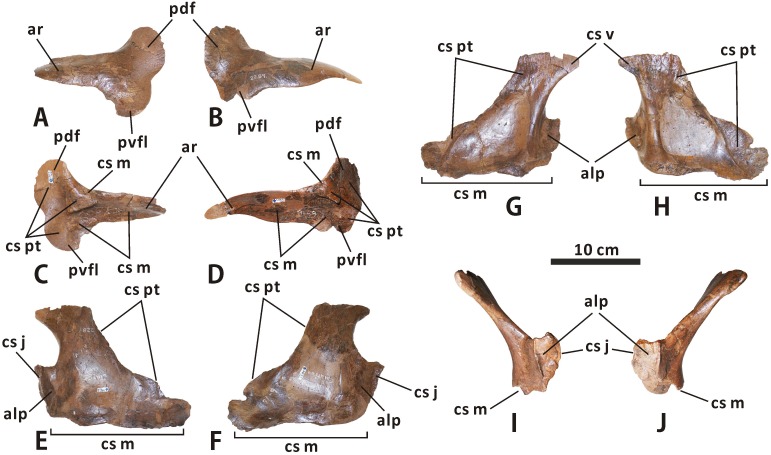
Left and right ectopterygoids of *Edmontosaurus regalis* (CMN 2289) in lateral (A, B) and medial (C, D) views. Left and right palatines of *Edmontosaurus regalis* (CMN 2289) in lateral (E, F), medial (G, H), and anterior (I, J) views.

**Ectopterygoid** (Figs 6 and 13). The ectopterygoid is a dorsoventrally flattened bone that comprises a long anterior ramus and a pair of tongue-like posterior flanges. This element is best preserved but partially reconstructed with plaster in CMN 2289. The anterior ramus covers most of the dorsal surface of the ectopterygoid shelf as it gradually narrows mediolaterally towards the external naris. This ramus is proportionately broader than in *Maiasaura* and *Brachylophosaurus*. A rimmed, subtriangular eminence adjacent to both the anterior ramus and posterodorsal flange projects medially from the dorsomedial margin of the ectopterygoid. When in articulation, the eminence is tightly locked between the pterygoid process of the maxilla anterodorsally and the posterior part of the ectopterygoid shelf ventrally, and is posteriorly and medially buttressed by the pterygoid. The posteromedial surface of the entire posterodorsal flange, together with the medial surface of the posterior half of the posteroventral flange, constitutes a gently concave, auriform sutural surface for the pterygoid.

**Palatine** (Figs 3, 4, 6 and 13). The palatine is best preserved and exposed in CMN 2289. The element has a thin, subtrapezoidal main plate, and a dorsoventrally expanded anterolateral process. The main plate progressively decreases in height along its posterior two thirds, and is anteromedially limited by a slightly anteriorly curved, cylindrical strut. In anterior view, the dorsal part of the plate is moderately inclined medially relative to the vertical base of the bone. Medially, a large, ovate fossa occupies the centre of the main plate. The palatine would receive the palatine ramus of the pterygoid along the sharp dorsal margin and rugose dorsal region of the ventromedial surface of its main plate. The ventromedial surface of the pointed anterodorsal corner of the main plate forms a subtriangular sutural surface for the vomer, which is marked by fine, anterodorsally-posteroventrally oriented striations. The anterolateral process extends at an angle of 110° from the anteroventral region of the main plate, and would contact the posteromedial margin of the maxillary anterior process. A low, vertically oriented ridge lies on the anteromedial side of the anterolateral process. The process probably defines the internal antorbital fenestra posteriorly, as pointed out by Horner [45].

**Fig 14:**
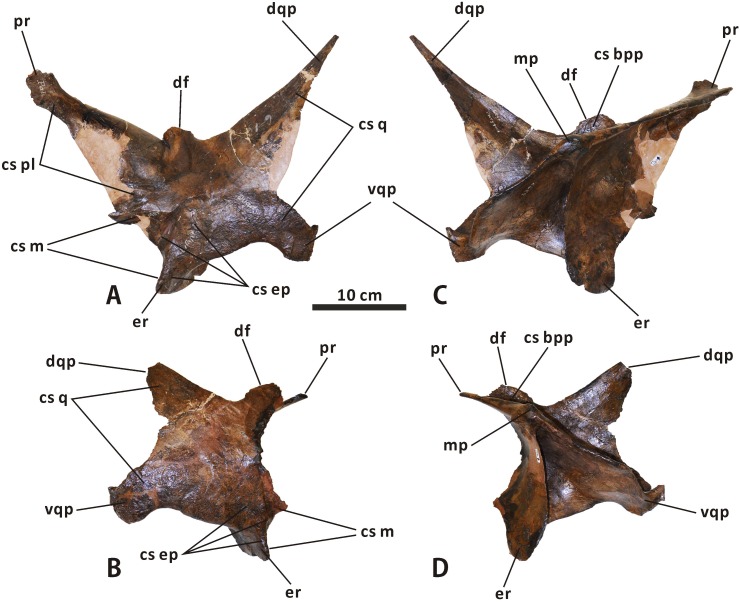
**Left and right pterygoids of *Edmontosaurus regalis* (CMN 2289) in anterolateral (A, B) and posteromedial (C, D) views**.

**Pterygoid** (Figs 3, 4 and 14). The pterygoid has been well illustrated and described for *Edmontosaurus regalis* [16, 46], based on CMN 2289. This element does not differ significantly from that of other hadrosaurids. The dorsal flange has an oval, posteromedially-facing, anterodorsally-posteroventrally elongate articular surface for the basipterygoid process of the basisphenoid. The articular surface is slightly proportionately larger than that of *Brachylophosaurus*. The prominent medial ridge of the ventral quadrate process in CMN 2289 is nearly straight, in contrast to the strongly anterolaterally convex equivalent in *Hypacrosaurus* (e.g. ROM 702) and *Corythosaurus* (e.g. CMN 8676).

**Vomer** (Fig 4). The vomer is a long, mediolaterally thin, subtriangular bone that gradually tapers anteriorly. This bone contacts the premaxilla anteriorly, the maxilla anterolaterally, the palatine posterodorsally and laterally, the pterygoid posteromedially, and its counterpart dorsomedially. In CMN 2288, the anterior parts of the paired vomers are visible throughout the external naris.

#### Neurocranium

**Fig 15:**
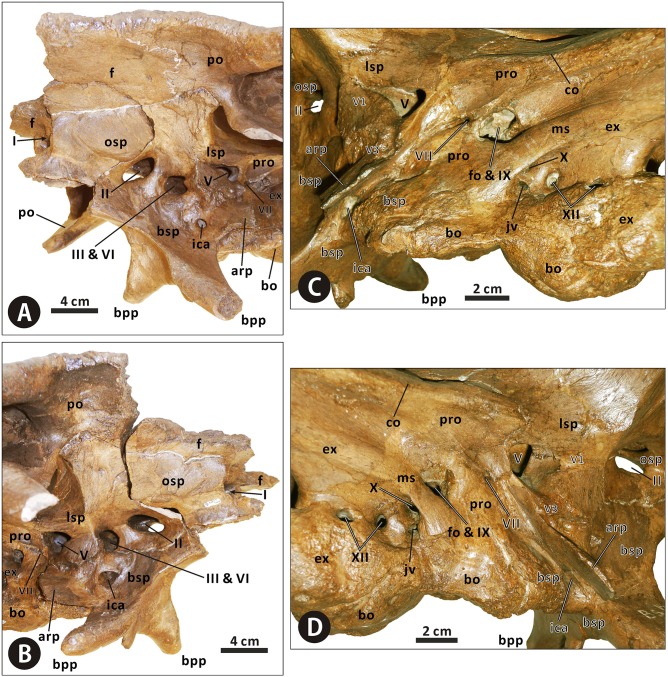
Anterior region of the neurocranium of *Edmontosaurus regalis* (CMN 2289) in left anterolateral (A) and right anterolateral (B) views. Posterior region of the neurocranium of *Edmontosaurus regalis* (CMN 2289) in left lateral (C) and right lateral (D) views.

**Orbitosphenoid–presphenoid complex** (Figs 4, 10–12 and 15). The presphenoid and orbitosphenoid are fused into a single unit in CMN 2288 and CMN 2289. The two bones form the anterodorsal part of the lateral wall of the braincase, and enclose most of the ventral region of the prosencephalon. The left and right presphenoids converge ventromedially to meet each other along the sagittal plane, and produce a reniform anterior exit of the olfactory nerve, together with the paired frontals. The ventral half of the orbitosphenoid is pierced by a large, oval foramen for the optic nerve (CN II), just above its nearly horizontal, weakly sinuous suture with the basisphenoid–parasphenoid complex. The posteroventral corner of the orbitosphenoid is slightly notched to form the anterodorsal margin of the circular foramen for the occulomotor nerve (CN III) and abducens nerve (CN VI). This foramen is located immediately laterodorsal to the pituitary fossa formed by the basisphenoid. In CMN 2288, the paired presphenoids contact the median cultriform process of the parasphenoid ventrally along the straight lateral sutures; the dorsal region of the orbitosphenoid is perforated by a small foramen that probably conducted the trochlear nerve (CN IV).

**Laterosphenoid** (Figs 4, 10–12 and 15). The laterosphenoid is a subtriangular bone that encloses the mesencephalon and posterior end of the prosencephalon laterally, between the orbitosphenoid and prootic. The bone consists of a stout dorsolateral process and a large temporal plate. The dorsolateral process is moderately shortened, about 70% the length of the temporal plate. By contrast, the equivalent ratio is greater than 90% in all other hadrosauroids except *Edmontosaurus annectens*. In CMN 2288 and CMN 2289, the blocky distal end of the dorsolateral process fits into a deep pit on the ventromedial surface of the postorbital main body. Anteriorly, the laterosphenoid has a strongly interdigitated union with the orbitosphenoid, where the suture between the two bones is unclear but discernable. The anteroventral region of the laterosphenoid gently curves medially to form the posterodorsal margin of the foramen for the occulomotor and abducens nerves. Posteriorly, the large, subcircular foramen for the trigeminal nerve (CN V) occurs along the contact between the laterosphenoid and prootic [47]. This foramen measures 25 mm in diameter. On the lateral surface of the temporal plate, a wide, horizontal sulcus for the ophthalmic branch (CN V1) extends anteriorly from the trigeminal foramen. Just below the trigeminal foramen, a narrow, anteroventrally directed groove extending onto the lateral surface of the basisphenoid indicates the passage for the mandibular branch (CN V3), as in other hadrosaurids [35, 38, 40].

**Prootic** (Figs 10, 11 and 15). The prootic is located centrally on the lateral wall of the braincase, and probably protected the metencephalon laterally. The bone bears an elongate, subrectangular posterolateral process between its contacts with the parietal and exoccipital. In lateral view, the anterior portion of the prootic exhibits a shallowly arcuate embayment that forms the posterior margin of the trigeminal foramen. Ventrally, the element is seamlessly fused to the basisphenoid. Posterior to the trigeminal foramen, the central body of the prootic is pierced by a small foramen for the facial nerve (CN VII). This foramen is contiguous with two narrow grooves on the lateral surface of the bone, which are directed posterodorsally and anteroventrally, respectively. The posterodorsal groove is slightly shorter and more poorly defined than the anteroventral one, and conducted the hyomandibular branch of the facial nerve [38, 47]. The second groove accommodated the palatine branch of the same cranial nerve, as in other hadrosaurids [38, 47]. The prootic defines the large fenestra ovalis anterodorsally, where the bone contacts the exoccipital posteriorly. The ventral part of the prootic–exoccipital suture cannot be observed in CMN 2289, but it is visible in juvenile *Edmontosaurus* (e.g. UAM ES4357). In CMN 2289, a nearly horizontal crista otosphenoidalis is well developed on the dorsolateral surface of the prootic; the crista emerges from the anterolateral margin of the bone, and extends posterolaterally onto the anterodorsal surface of the proximal region of the paroccipital process. In comparison, the crista otosphenoidalis of *Brachylophosaurus*, *Kritosaurus* and *Gryposaurus* is relatively slender and strongly inclined posterodorsally.

**Exoccipital–opisthotic complex** (Figs 3, 4, 10–12 and 15). In all specimens of *Edmontosaurus regalis*, the exoccipital and opisthotic are fully fused together, with no distinguishable suture between the two bones. Therefore, the exoccipital–opisthotic complex is herein abbreviated as the exoccipital. The paired exoccipitals probably surrounded the myelencephalon posterolaterally at the posterior end of the braincase. Either element is composed of a short occipital plate, a robust paroccipital process, a tall posteromedial shelf, and a bulbous condyloid.

The anterior region of the occipital plate circumscribes most of the fenestra ovalis posteroventrally. The fenestra ovalis is partially divided internally by a prominent, anteroventrally directed lamina, namely the crista interfenestralis. The posteroventral part of the fenestra may represent the lateral exit for the glossopharyngeal nerve (CN IX). The metotic strut adjacent to the fenestra ovalis is continuous posterodorsally with the anteroventral region of the paroccipital process. Further posteriorly, the lateral surface of the base of the occipital plate is pierced by four small oval foramina that are nearly equal in size. Directly below the robust metotic strut, the first three of these foramina are close to one another, and lie a short distance from the last one. The dorsal one of the first three foramina probably transmitted the vagus nerve (CN X), while the anteroventral one likely acted as the exit for the jugular vein [47, 48]. The combination of the two foramina is possibly homologous with the metotic foramen, as described by Gower and Weber [49]. By contrast, there is no single exit for the jugular vein beneath the vagus foramen in *Brachylophosaurus*, *Kundurosaurus*, and *Saurolophus*. In CMN 2289, the two more posteriorly located foramina probably conducted the branches of the hypoglossal nerve (CN XII). A thick, nearly vertical septum separates the vagus foramen from the foramen for the anterolateral branch of the hypoglossal nerve, as in *Shantungosaurus giganteus* (e.g. GMV 1780–1) and *Edmontosaurus annectens* (e.g. ROM 64623). This condition differs from the thin, strongly posteriorly inclined septum observed in *Gryposaurus notabilis* (e.g. AMNH 5350).

The condyloid is large, posteriorly projected and lateroventrally convex, with a fairly rugose lateral surface. It contacts the unpaired basioccipital ventrally along a slightly sinuous suture, and forms the laterodorsal part of the reniform occipital condyle. The condyloid is crescentic in occipital view, whereas in *Gryposaurus*, *Kritosaurus* and juvenile *Edmontosaurus* the posterior outline of the condyloid is either subtriangular or trapezoidal. The paroccipital process is an elongate, horn-shaped projection that extends and curves lateroventrally and slightly anteriorly from the posterodorsal region of the occipital plate. In occipital view, the left and right paroccipital processes lock the posterior end of the supraoccipital in between. The proximal part of the process is strongly expanded anteroventrally-posterodorsally, where its dorsal surface is slightly more dorsally positioned than the posterodorsal edge of the supraoccipital. In adults, *E*. *regalis* has a relatively shorter and more robust paroccipital process than *Gryposaurus*, *Kritosaurus* and *Prosaurolophus* of similar size. The posteromedial shelf contacts its complement along a prominent median ridge, and together they form a deeply depressed, strongly posterodorsally tilted posterior wall of the braincase above the foramen magnum.

**Supraoccipital** (Figs 4, 10 and 12). The supraoccipital, located immediately dorsal to the united exoccipitals, does not differ in any significant aspect from that of other hadrosaurids. Only the posterior region of the bone is visible in CMN 2288 and CMN 2289. In occipital view, the lateral boss in the posterolateral region of the dorsal surface of the supraoccipital invades the medial ramus of the squamosal. The posterior surface of the supraoccipital is dorsoventrally low and moderately anterodorsally inclined; it has numerous fine horizontal fissures along the entire width of the bone. This condition contrasts with the relatively smooth, nearly vertical posterior side of the supraoccipital in many non-hadrosaurid iguanodontians, such as *Dakotadon lakotaensis* (SDSM 8656) and *Probactrosaurus gobiensis* (e.g. PIN 2232/17-1). In CMN 2289, the supraoccipital does not wedge anteriorly into the laterally exposed contact between the parietal and prootic.

**Basisphenoid–parasphenoid complex** (Figs 4, 10–12 and 15). The basisphenoid is indistinguishably fused with the parasphenoid at late ontogenetic stages. The two unpaired bones together constitute the anteroventral part of the braincase, the centre of which probably accommodated the pituitary gland dorsally. The elongate cultriform process of the parasphenoid curves anterodorsally from the base of the basisphenoid, where the lateroventrally directed basipterygoid processes diverge from each other, forming an angle of approximately 125°. Each basipterygoid process is rod-shaped and distally tapered into a blunt point. The anterior side of the upper half of the process gradually narrows dorsomedially to form a sharp margin. The margin well defines the slightly concave, smooth anteroventral surface between the basipterygoid processes, which is located directly anterior to the transverse ridge and its small median protuberance. Posterior to the base of the basipterygoid processes, the posteroventral region of the basisphenoid contacts the basioccipital along weakly undulating lateral and ventral sutures, and forms the anterior two thirds of the sphenooccipital tubera. The sphenooccipital tubera are very rugose and roughly bulboid. They are ventrally separated by a shallow midline groove.

In lateral view, the basisphenoid–parasphenoid complex constitutes the ventral margin of the foramen for the occulomotor and abducens nerves. Posteriorly, the basisphenoid does not appear to participate in the formation of the trigeminal foramen, because of the presence of a sinuous, anteroposteriorly oriented lateral suture between the basisphenoid and laterosphenoid well below the foramen and immediately anterior to the dorsal part of the alar process. The ventral part of the alar process anterolaterally conceals a small oval foramen that is interpreted as the lateroventral entrance for the carotid canal (i.e. the passage of the internal carotid artery) [35, 38, 47]. Anteriorly, the endocranial surface of the basisphenoid is deeply excavated by a dorsoventrally tall pituitary fossa, located anteroventral to the endocranial floor. The left and right carotid canals extend anterodorsally and medially from their lateroventral entrances, through the basisphenoid, onto the posteroventral surface of the pituitary fossa, where a pair of large, round foramina occur. Dorsal to the internal exits for the left and right carotid canals, two relatively small foramina on the posterodorsal surface of the pituitary fossa open posterodorsally onto the dorsum sellae (i.e. the anterior end of the endocranial floor) via another two small foramina. The two pairs of foramina probably represent the endocranial openings of the left and right abducens nerves [47].

**Basioccipital** (Figs 4, 10–12 and 15). The basioccipital forms the posterior third of the sphenooccipital tubera, the posterior two thirds of the endocranial floor, and the ventral portion of the occipital condyle. This element is subtrapezoidal in occipital view. The ventral surface of the posterior half of the basioccipital is strongly convex mediolaterally, and possesses numerous short, randomly oriented indentations. As in *Edmontosaurus annectens* (e.g. AMNH 427) and *Shantungosaurus giganteus* (e.g. ZCDM HS0001), the occipital condyle is strongly deflected posteroventrally, forming an angle of approximately 42° with the anterior region of the skull roof. By contrast, in all other hadrosaurids, the corresponding deflection angle is no more than 25° [10].

#### Lower jaw

**Fig 16:**
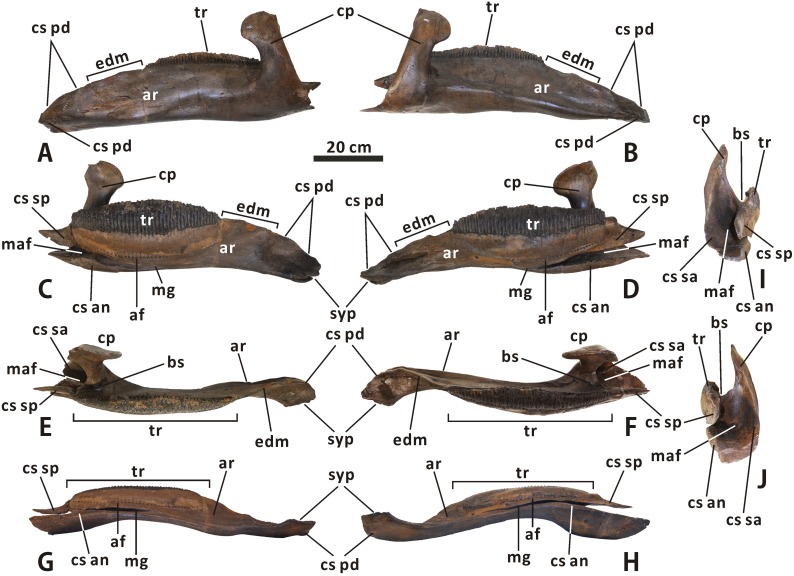
**Left and right dentaries of *Edmontosaurus regalis* (CMN 2289) in lateral (A, B), medial (C, D), dorsal (E, F), ventral (G, H), and posterior (I, J) views**.

**Fig 17:**
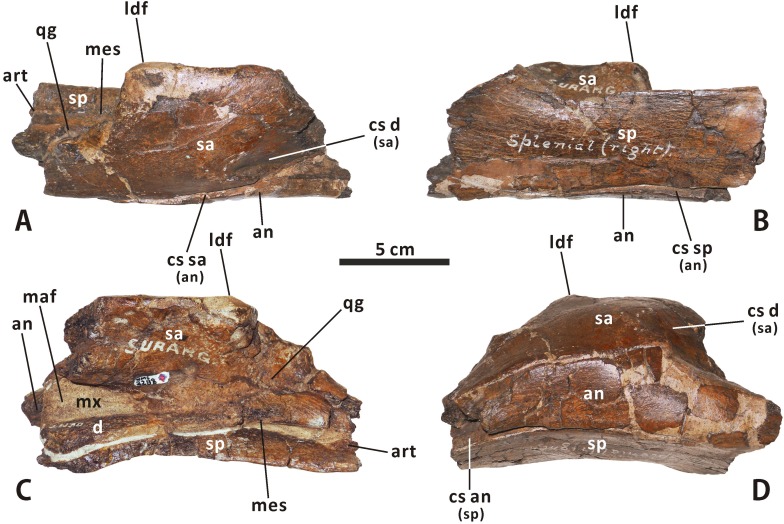
**Incomplete, articulated right dentary, surangular, angular, splenial and articular of *Edmontosaurus regalis* (CMN 2288) in lateroventral (A), dorsomedial (B), dorsal (C), and ventral (D) views**.

**Dentary** (Figs 3, 4, 16 and 17). Both the left and right dentaries are well preserved in CMN 2289, although the right one appears to be slightly distorted due to post-depositional dorsoventral crushing. The anterior part of the dentary ramus is gently anteroventrally deflected, forming an angle of 14° with the horizontal posterior part of the ramus. In comparison, the ventral deflection of the dentary anterior part is much stronger in *Kritosaurus* (e.g. AMNH 5799) and *Gryposaurus* (e.g. ROM 873), with a relatively steep dorsal contact surface for the predentary. Posterior to the symphysial process, the edentulous region of the dentary in CMN 2289 is approximately 38% as long as the tooth row. In fact, the ratio between the length of the edentulous region and that of the tooth row varies significantly among the adult dentaries of *Edmontosaurus regalis*, ranging from 0.34 to 0.50. The dentary has an anteroposteriorly elongate, subelliptical tooth row, the lingual surface of which is slightly anteroposteriorly and dorsoventrally convex. The tooth row bears 48 vertical tooth positions and a transversely narrow, dorsolaterally concave occlusal surface. In medial view, the long axis of the tooth row parallels the ventromedial margin of the middle part of the dentary ramus. This condition contrasts with the modestly anterodorsally inclined long axis of the tooth row in *Acristavus gagslarsoni* (e.g. MOR 1155) and *Probrachylophosaurus bergei* (MOR 2919). The tooth row extends slightly posterior to the coronoid process. As in all other hadrosaurines except Brachylophosaurini, the apex of the coronoid process in *E*. *regalis* is subcircular and anteroposteriorly expanded, with a pronounced posterior extension. The buccal shelf between the base of the coronoid process and the posterior end of the tooth row is mediolaterally broad and deeply depressed. Posteriorly, the anterior region of the mandibular adductor fossa becomes progressively narrower and shallower towards the apex of the coronoid process.

**Fig 18:**
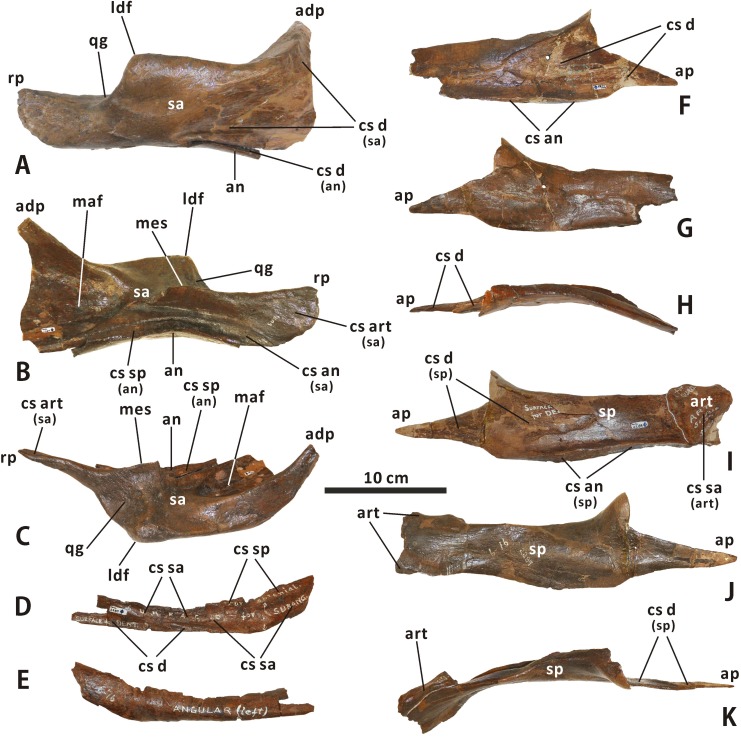
Incomplete, articulated right surangular and angular of *Edmontosaurus regalis* (CMN 2289) in lateroventral (A), dorsomedial (B), and dorsal (C) views. Incomplete left angular of *Edmontosaurus regalis* (CMN 2289) in laterodorsal (D) and ventromedial (E) views. Partial right splenial of *Edmontosaurus regalis* (CMN 2289) in lateral (F), medial (G), and dorsal (H) views. Nearly complete, articulated left splenial and articular of *Edmontosaurus regalis* (CMN 2289) in lateral (I), medial (J), and dorsal (K) views.

**Surangular** (Figs 3, 4, 17 and 18). The surangular morphology of *Edmontosaurus regalis* closely resembles that of other hadrosaurines: the posterior half of the element is dorsoventrally shortened, with a strongly ventrally twisted dorsolateral flange and a nearly straight, posteriorly directed retroarticular process. The anterior half of the surangular is markedly dorsomedially excavated by the posterior region of the mandibular adductor fossa that has a deeply arcuate edge contour, and is laterally shaped by a mediolaterally thin, ascending anterodorsal process. The anterodorsal process occurs directly dorsal to the posterior region of the mandibular adductor fossa. The posteromedial margin of the former is continuous posteroventrally with the dorsal edge of the latter. In articulated mandibles, including CMN 2288, most of the lateral surface of the anterodorsal process is concealed by the lateral wall of the triangular depression on the posterior surface of the dentary coronoid process, very similar to the condition in other hadrosaurids. By contrast, in many basal hadrosauroids such as *Equijubus normani* (IVPP V12534), only the anterior half of the anterodorsal process is laterally covered by the depressed posterior part of the coronoid process when in articulation.

In dorsal view, the surangular is modestly curved posteromedially near the centre, where the subtriangular laterodorsal flange emerges; the deflection angle along the surangular ventromedial margin is approximately 153°. The thickened ventromedial margin of the surangular produces an elongate, slightly convex contact surface for the angular. A narrow, subtriangular shelf extends medially and slightly dorsally from the dorsal surface of the middle of the surangular, where the oval glenoid is present. As in *Gryposaurus monumentensis* (RAM 6797), the anterior part of the medial shelf abuts the posteroventral edge of the mandibular adductor fossa. In CMN 2288, the medial shelf of the surangular does not meet the splenial; a distinct gap occurs between them. The condition is also observed in *Maiasaura* (e.g. ROM 44770) and *Brachylophosaurus* (e.g. CMN 8893). The glenoid between the laterodorsal flange and medial shelf is gently concave and marked by numerous small pits and fine striations. It would probably articulate with the entire lateral condyle and lateral third of the medial condyle of the quadrate dorsally in CMN 2289. The posteroventral corner of the retroarticular process is broadly arched in lateral view, contrasting with the nearly right-angled posteroventral margin of the process seen in most lambeosaurines, such as *Lambeosaurus magnicristatus* (e.g. TMP 66.04.01) and *Parasaurolophus walkeri* (e.g. ROM 768).

**Angular** (Figs 17 and 18). The angular is the longest post-dentary bone of the mandible. The bone is laterodorsally-ventromedially compressed, and is slightly curved dorsomedially along its posteror third. The complicated laterodorsal surface of the surangular contacts the dentary anteroventrally, the surangular posterodorsally, and the splenial dorsally. The paired angulars of CMN 2289 are missing their anterior thirds and posteriormost ends. In dorsolateral view, the flat contact surface for the dentary reaches its maximum depth at the anterior third of the bone, the dorsomedial and lateroventral margins of which well define the surface. Further posteriorly, the aforementioned surface gradually decreases in depth due to the emergence and widening of the gently concave, more dorsally positioned sutural surface for the surangular, and terminates at about 40% the length of the angular from the posterior margin. Just above the surangular facet, the contact surface for the splenial occurs along the posterior half of the dorsal side of the bone. The surface is bent and relatively narrow. In CMN 2288, the angular is completely obscured laterally by the surangular.

**Splenial** (Figs 3, 17 and 18). There is no evidence of a single prearticular posterolaterally overlapping the splenial in all available *Edmontosaurus regalis* material. Therefore, the prearticular and splenial might be completely fused to each other before the juvenile stages. The left and right splenials of CMN 2289 are disarticulated and well preserved, yet slightly reconstructed with plaster. The bluntly round posterior end is missing in both elements. The splenial is mediolaterally flattened, extending anteriorly to form an elongate, slender anterior process that accounts for approximately 27% the length of the bone. The lateral side of the process contributes to the anterior region of the contact surface for the dentary. Posterior to the anterior process, the remainder of the dentary facet is subtriangular and tapered posteriorly. It terminates adjacent to the midpoint of the lateral surface of the splenial. Ventrally, the contact surface for the angular is smooth and transversely broad. It protrudes laterally to produce a short, dorsoventrally thin shelf along the posterior two thirds of the bone. The shelf does not meet the ventromedial margin of the surangular in CMN 2288. Furthermore, the splenial in *E*. *regalis* is slightly more posteromedially curved than that in *Brachylophosaurus canadensis*.

**Articular** (Figs 17 and 18). In CMN 2289, the left articular contacts the posterior portion of the ipsilateral splenial medially along the sinuous anterior and dorsal sutures, and is missing its posterodorsal region. This element is approximately quadrangular in lateral view, and exhibits a dorsoventrally tall, subtriangular posterior outline that gradually narrows ventrally. The subtly concave contact surface for the surangular faces lateroventrally and slightly posterorly. The mediolaterally thickened dorsal part of the bony articular does not appear to form the medial region of the mandibular glenoid, contra Lambe [16]. This is because the bone does not meet the more anterolaterally positioned medial shelf of the surangular that limits the lateral two thirds of the mandibular glenoid medially, as in CMN 2288. We speculate that the articular would have a cartilaginous anterior extension in life. The extension would fill the gap between the surangular and splenial, and would contact the medial two thirds of the medial condyle of the quadrate dorsally.

#### Dentition

**Fig 19:**
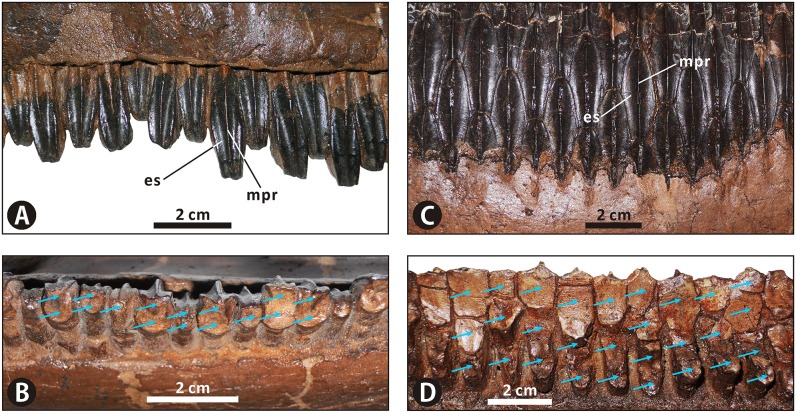
Dentitions of *Edmontosaurus regalis* (CMN 2289). Exposed teeth in the middle of the left maxilla in labial (A) and occlusal (B) views. Exposed teeth in the middle of the left dentary in lingual (C) and occlusal (D) views. Positions of active teeth on the occlusal surface are indicated by blue arrows.

**Maxillary teeth** (Figs 3–6 and 19). The left and right maxillae in CMN 2289 possess 53 and 51 alveoli, respectively. Only active teeth along the occlusal surface are recognizable in CMN 2289 because replacement teeth of the dental battery are entirely obscured from view by the thin medial parapet of the maxilla. In the middle dental battery, most of the occlusal surface is formed by two functional teeth per alveolus, namely a worn crown and a residual, more medially situated root. A small minority of alveoli along the middle part of the dental battery hold a single functional tooth for each position, which is applied to most of the alveoli along the anterior and posterior regions of the battery. The enameled labial surface of each tooth crown is diamond-shaped and evenly separated by a straight primary ridge. The mesial and distal margins of the apical half of the surface is poorly denticulated, contrasting with the large mammillate denticles seen in Brachylophosaurini. Of note, the maxillary tooth crowns of CMN 2289 are mesiodistally narrower and apicobasally shorter than the dentary ones. The tooth crown in the middle of the maxilla measures 8 mm in average maximum width, and has an estimated height of 29 mm.

**Dentary teeth** (Figs 3, 4, 16 and 19). The dentary dentition of *Edmontosaurus regalis* has been well studied by Lambe [16] and Xing et al. [10]. The middle region of the dental battery in CMN 2289 shows a maximum of seven teeth per alveolus, including 3–4 functional teeth on the occlusal surface. The enameled lingual surface of each tooth crown in the middle of the dental battery is at least 37 mm high and 12 mm wide, with a height/width ratio ranging from 2.90 to 3.16. By contrast, tooth crowns in the adult dentaries of *Edmontosaurus annectens* are slightly mesiodistally wider, and have height/width ratios up to 2.70. In CMN 2289, a median primary ridge runs longitudinally throughout the entire lingual surface of each tooth crown. This condition differs from the slightly distally offset primary ridge seen in *Rhinorex condrupus* [36].

## Discussion

### Comparison with *Edmontosaurus annectens*

*Edmontosaurus regalis* and *E*. *annectens* are the only two valid species of the genus *Edmontosaurus* [8]. Our osteological evaluation of the types of *E*. *regalis* further confirms the subtle morphological differences between this taxon and *E*. *annectens*. *Edmontosaurus regalis* is distinguished from *E*. *annectens* by the following nine non-autapomorphic characters consistently expressed in large, presumably adult specimens: 1) the prenarial region of the snout is relatively short anteroposteriorly, being no more than 25% as long as the skull; 2) the cranium is relatively short anteroposteriorly and dorsoventrally high, with a length/height ratio less than 2.10; 3) the posterodorsally reflected anterior margin of the premaxilla forms a strongly swollen, lip-like oral region of the skull; 4) the external interfrontal suture is proportionately longer because the posteromedial processes of the nasals are slightly inserted posteriorly into the frontals; 5) the prominent arched posterodorsal margin of the circumnarial fossa ascends to the dorsal surface of the narial region of the snout; 6) the jugal process of the postorbital strongly bulges laterally, giving rise to a greater development of the postorbital pocket; 7) the dorsal suture between the nasal and frontal is crenulated and mediolaterally oriented; 8) the subtriangular vestibular promontory between the two premaxillary foramina in the prenarial region of the circumnarial fossa is anteromedially-posterolaterally narrow, and therefore bounds a relatively large anteromedial concavity posterolaterally; 9) each tooth crown in the middle of the dentary is slightly mesiodistally narrower, with a height/width ratio greater than 2.90. In this paper, the last three characters are newly identified and subsequently added to the diagnosis of *E*. *regalis*. Furthermore, in *E*. *regalis*, the dorsal part of the jugal anterior process strongly flares posterolaterally to form a sharp, posterodorsally tilted projection that invades the orbital margin. This feature is in marked contrast with the smoothly continuous posterodorsal surface of the jugal anterior process acting as an indistinct, nearly vertical anteroventral margin of the orbit in all other iguanodontians, and is herein considered autapomorphic for *E*. *regalis*.

### Status of *Ugrunaaluk kuukpikensis*

Mori et al. [50] erected a new genus and species of Edmontosaurini, *Ugrunaaluk kuukpikensis*, based mostly on juvenile material from the Liscomb bonebed in the Price Creek Formation of northern Alaska. These authors argued that *U*. *kuukpikensis* is taxonomically distinct from *Edmontosaurus* in its possession of the following three diagnostic character: (1) the outer narial fossa of the circumnarial depression is laterodorsally marked by a transverse ridge, lacking a vestibular promontory; (2) the main body of the postorbital has a shallow internal fossa at the base of the jugal process; (3) the posteromedial rim of the anterior process of the jugal is strongly angled. They further distinguished *U*. *kuukpikensis* from *E*. *regalis* based on (4) the wide lateral exposure of the quadratojugal in the former taxon. Here we regard this differential diagnosis of *U*. *kuukpikensis* as problematic, and outline the reasons for questioning the validity of the species below.

The *Ugrunaaluk* material probably pertains to very young individuals much smaller than most comparable material available for *Edmontosaurus*. For example, the *Ugrunaaluk* specimens are approximately 50%–65% as long as the equivalent bones of the juvenile *Edmontosaurus* from Wyoming (ROM 53492–53541). Mori et al. [50] note that there is some overlap between *U*. *kuukpikensis* and *E*. *annectens* in their size classes 2 and 3, but admit that the overlap involves very few specimens. There is no size overlap whatsoever between known material of *U*. *kuukpikensis* and *E*. *regalis*. The authors were therefore forced to draw direct comparisons between the juvenile material of *U*. *kuukpikensis* and presumably adult material of *E*. *regalis* and *E*. *annectens*, establishing a differential diagnosis for the first taxon on the basis of arguably juvenile character states. Given the relatively small size and juvenile stages of the *U*. *kuukpikensis* material, the scarcity of size-overlapping material between *U*. *kuukpikensis* and *Edmontosaurus*, and the closest affinities between the two taxa, we cannot reject the following hypothesis: characters 1, 2, 3, and 4 may be ontogenetically variable in individuals of *U*. *kuukpikensis*, and therefore possibly represent transient states during the juvenile stages of *Edmontosaurus*. In fact, the development of the premaxillary vestibular promontory and postorbital internal fossa, as well as the decrease in width of the lateral exposure of the quadratojugal, is certainly most pronounced in the largest, presumably oldest individuals of *Edmontosaurus* [8].

The limited available size-equivalent material for *Ugrunaaluk kuukpikensis* and *Edmontosauru*s further led Mori et al. [50] to draw comparisons, not simply between the fossils themselves, but between growth trajectories established on the basis of relatively few data points (3–8 points for *U*. *kuukpikensis*). While we applaud the authors’ meticulousness, this approach is problematic, given the issues inherent with establishing a growth trajectory based on just a few specimens from one end of an ontogenetic spectrum [51].

With these considerations in mind, we see no compelling reason to accept that *U*. *kuukpikensis* represents a valid taxon. We instead regard the Alaskan material conservatively as *Edmontosaurus* sp. [8, 20]. We further suggest the possibility, as indicated by Xing et al. [10], that this material represents a northern occurrence of *E*. *regalis*, as it displays some salient features characteristic of the species. These include a relatively short prenarial region of the premaxilla with a posterodorsally reflected, lip-shaped oral margin (e.g. UAM ES4184), and a wide, anteroventrally tilted shelf at the base of the postorbital sutural surface of the jugal (e.g. UAM ES4187 and UAM ES4241) [10, 50]. Such an interpretation would have interesting implications regarding migration scenarios along latitudinal clines [52]. Finding more juvenile material attributable to *E*. *regalis*, and/or more adult material attributable to *U*. *kuukpikensis*, will go a long way toward resolving this taxonomic conundrum.

### Phylogenetics of Hadrosauridae

**Fig 20:**
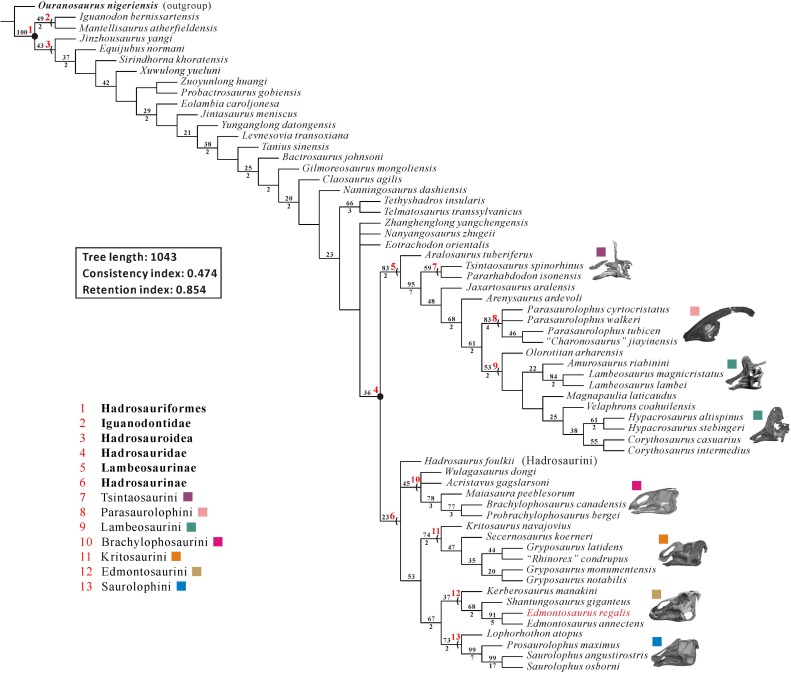
Strict consensus of 24 most parsimonious trees resulting from the maximum parsimony analysis of Hadrosauroidea, showing the sister-group relationship between Hadrosaurinae and Lambeosaurinae within Hadrosauridae. Numbers above lines represent bootstrap proportions, whereas those below lines represent Bremer decay values. Bootstrap proportions lower than 20 and Bremer decay values less than 2 are not shown.

A phylogenetic analysis of Hadrosauroidea resulted in 24 most parsimonious trees (MPTs) of 1037 steps, each with a consistency index of 0.476 and a retention index of 0.856. The strict consensus of the recovered MPTs reveals that the sister taxon to Lambeosaurinae consists of Brachylophosaurini, Kritosaurini, Saurolophini and Edmontosaurini, as well as Hadrosaurini that is solely represented by the enigmatic *Hadrosaurus foulkii* (Fig 20). In other words, *H*. *foulkii* is found to be firmly within the monophyletic group comprising all non-lambeosaurine hadrosaurids. Thus, we agree with the taxonomic scheme of Hadrosauridae both argued by Lull and Wright [7] and Sereno [5], where Hadrosauridae is divided into two clades, namely Hadrosaurinae and Lambeosaurinae. *H*. *foulkii*, Brachylophosaurini, and the clade of Kritosaurini + (Saurolophini + Edmontosaurini) form an unresolved polytomy at the base of Hadrosaurinae.

Based on the topology of the strict consensus tree, Edmontosaurini is depicted as the clade consisting of all hadrosaurines closer to *Edmontosaurus* than to *Saurolophus*, *Kritosaurus*, *Brachylophosaurus*, or *Hadrosaurus*. This clade is tentatively composed of *Kerberosaurus manakini*, *Shantungosaurus giganteus*, *E*. *regalis*, and *E*. *annectens*. The two valid species of *Edmontosaurus* together form a monophyletic group highly nested within Edmontosaurini, and are united by multiple synapomorphies: characters 27(1), 38(3), 48(2), 182(1), 221(1), 324(1), and 327(0). Node support for the clade of *E*. *regalis* + *E*. *annectens* is extremely high, with a bootstrap proportion of 91% and a Bremer decay value of 5 (Fig 20). A suite of synapomorphies, namely 1(2), 181(2), 183(1), 204(1), 234(0) and 309(2), definitely identify *Shantungosaurus* as the sister taxon to *Edmontosaurus*, which further corroborates the hypothesis of Xing et al. [10] regarding the close relationship of the two genera. *K*. *manakini* is recovered at the base of Edmontosaurini.

The phylogenetic topologies of other major clades of Hadrosaurinae in the strict consensus tree are briefly described as follows. In the monophyletic Brachylophosaurini, *Probrachylophosaurus bergei* is recovered as the sister taxon to *Brachylophosaurus canadensis*, as documented by Freedman Fowler and Horner [42]; the clade of *B*. *canadensis* + *P*. *bergei* is posited as more closely related to *Maiasaura peeblesorum* than to *Acristavus gagslarsoni* and *Wulagasaurus dongi*. With regard to the clade Kritosaurini, *“Rhinorex” condrupus* is depicted as the sister taxon to *Gryposaurus latidens*, and is deeply nested within an otherwise monophyletic *Gryposaurus*. *Lophorhothon atopus* occupies the most basal position within Saurolophini.

It is worth noting that *Eotrachodon orientalis* is depicted as a relatively derived non-hadrosaurid hadrosauroid, rather than one of the most basal hadrosaurids as suggested by Prieto-Márquez et al. [34]. *Sirindhorna khoratensis* at the base of Hadrosauroidea is positioned higher on the tree than *Equijubus normani*, but below *Xuwulong yueluni*. Furthermore, our current analysis indicates that *Olorotitan arharensis* represents the first lineage of Lambeosaurini to branch off.

### Biogeography of Hadrosaurinae

**Fig 21:**
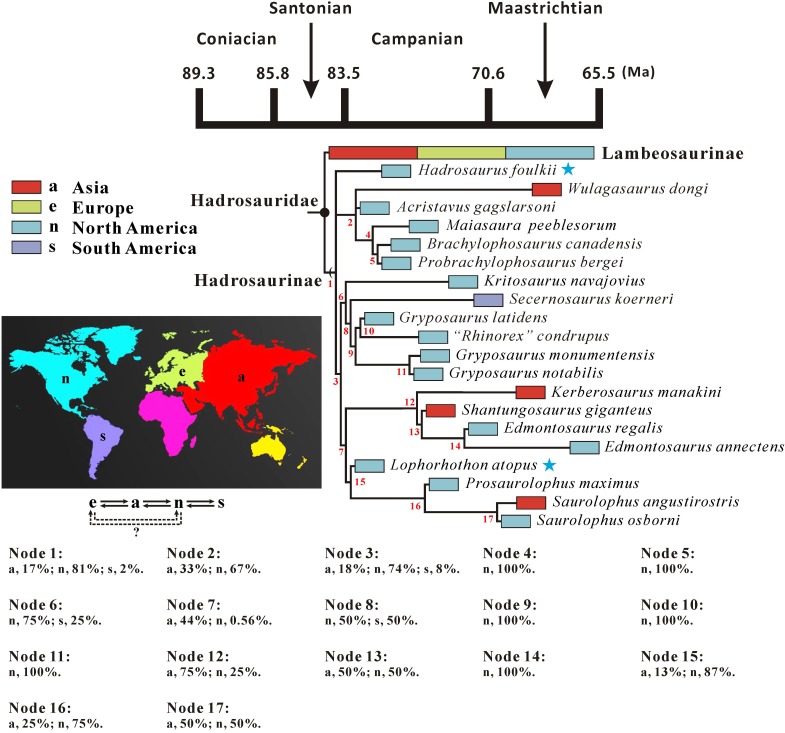
Time-calibrated cladogram of Hadrosaurinae derived from the strict consensus tree shown in Fig 20, with probability estimation of ancestral continental regions for all nodes of the taxon. Blue stars indicate hadrosaurine species recovered from Appalachia, eastern North America.

Since the 1980s, issues concerning the ancestral areas and dispersal dynamics of hadrosaurines during the latter half of the Late Cretaceous continue to be hotly debated [1, 2, 10, 30, 53–55]. Here, our temporally calibrated cladogram with probability calculation of ancestral areas provides some new insights into the biogeography of Hadrosaurinae (Fig 21). The results of our biogeographic analysis suggest the following information: Hadrosaurinae (node 1) has a considerably high probability (~81%) of having originated in North America; the first split of Hadrosaurinae is inferred to have occurred no later than the end of Santonian; Edmontosaurini (node 12) may originate in Asia, with a relatively high probability of 75%; during the latest Santonian–earliest Maastrichtian, multiple dispersals between Asia and western North America (i.e. Laramidia) would happen presumably via the Bering land bridge; the probabilities of the North and South American origins for Kritosaurini (node 6) are 75% and 25%, respectively (Fig 21).

Interestingly, the biogeographic scenario for the North American origin of Hadrosaurinae displays at least two Laramidia–Appalachia dispersal events around the boundary between the Santonian and Campanian, given the presence of *Hadrosaurus foulkii* and *Lophorhothon atopus* in lower Campanian exposures of Appalachia (i.e. eastern North America). This is inconsistent with the Laramidian restrictions for all known North American members of Tyrannosauridae and Ceratopsidae [56, 57], and conflicts with the paleogeographic hypothesis proposed by both Zharkov et al. [58] and Blakey [59], in which Laramidia was completely separated from Appalachia by the Western Interior Seaway during the Santonian to early Campanian. It is noteworthy that Hadrosaurinae (node 1) also has a probability of 17% indicating the ancestral area of Asia. In this biogeographic scenario, the occurrences of two hadrosaurine species in the lower Campanian of Appalachia could be explained as a result of pre-Campanian dispersals of some hadrosaurine clades from Asia to eastern North America throughout the European archipelago and landmass. Nevertheless, more fossil evidence from Asia and Europe in support of the hypothesis is still necessary.

Despite the fact that the North American origin of Hadrosaurinae is strongly favored by the biogeographic analysis, we cannot rule out the possibility that the clade originated in Asia. At present, the widely accepted paleogeographic reconstructions of North America in the Santonian–Campanian period demonstrate a strong bias against the biogeographic scenario for the North American origin of Hadrosaurinae. Similarly, the fossil record of Hadrosaurinae known from the Santonian and early Campanian of Asia is extremely scant: only a few isolated postcranial elements with hadrosaurine affinities have been reported; they were recovered from the middle Santonian of central China [60]. To better elucidate the biogeographic history of Hadrosaurinae, additional geological surveys and fossil collecting in Santonian and Campanian outcrops of Asia and Europe would be considerably beneficial.

## Methods

### Anatomical study

The osteological information of *Edmontosaurus regalis* described here was acquired by first-hand examinations and measurements of the types (CMN 2288 and 2289) publicly reposited at the Canadian Museum of Nature (CMN) in Ottawa (Ontario, Canada). Meanwhile, all necessary permits were obtained from the Paleobiology Section of the CMN for the described study, which complied with all relevant regulations. Comparative anatomical data from other iguanodontian taxa and referred material of *E*. *regalis* were collected by means of direct observation and from the relevant literature.

### Phylogenetic analysis

In order to assess the systematic position of *Edmontosaurus regalis* and phylogenetic topology of Hadrosauridae, a species-level cladistic analysis of Hadrosauroidea was conducted based on a data matrix comprising 62 taxa and 346 unordered, equally weighted characters. The non-hadrosauroid iguanodontian *Ouranosaurus nigeriensis* was constrained as the outgroup. Four recently-named hadrosauroid species (i.e. *Sirindhorna khoratensis*, *Eotrachodon orientalis*, *Probrachylophosaurus bergei* and *Rhinorex condrupus*) have been included in the analysis. The character list was slightly modified from Wang et al. [61], and consists of 235 characters pertaining to the cranium and 111 characters related to the axial and appendicular skeletons (see S1 Text). The character coding of *Kerberosaurus manakini* was based on morphological information collected from the material pertaining to this species and to its junior synonym *Kundurosaurus nagornyi* [10].

The character-taxon matrix (see S1 Dataset) was gathered in Mesquite version 3.10 [62], and was later analyzed in TNT version 1.1 [63], where maximum parsimony was used as the optimal criterion. A traditional search setting a random seed of 1 and 1000 replicates for Wagner trees was performed, under the tree bisection reconnection (TBR) swapping algorithm with 100 trees saved per replication. Bootstrap proportions and Bremer decay values for node support were calculated by the “Resampling” option with 1000 replicates of standard absolute frequencies and the “BREMER.RUN” script using minimum score in TNT, respectively. Phylogenetic definitions of Hadrosauriformes and its major clades largely follow Sereno [5], Brett-Surman [30], and Gates et al. [6].

### Biogeographic inference

The goals of our biogeographic analysis were to infer the ancestral areas and the latest split ages of Hadrosaurinae and its major clades. The result of the phylogenetic analysis served as the framework of the biogeographic investigations. The ancestral areas of nodes within Hadrosaurinae were inferred using probability calculation under the rules of multiplication and addition (see S2 Text). The rules are applicable to both bifurcate nodes and polytomies. Prior to calculation, each polytomy was transformed into multiple cladistic scenarios that all require a fully bifurcated topology. The evolutionary splitting events of Hadrosaurinae were putatively subject to dispersal and/or vicariance. We successively estimated the probabilities of ancestral areas for nodes from the tip to the base of the hadrosaurine phylogenetic tree derived from the strict consensus. The probabilities of ancestral areas of each node were completely determined by those of its subclades being equally treated. When a subclade corresponds to a species-level taxonomic unit, the ancestral area of the former would be restricted to the continental region where the material of the latter was recovered. The continental regions applied in this analysis were Asia, North America, and South America, where currently known hadrosaurine species have been recorded.

For the analysis, the presence of the Late Cretaceous land connections between Asia–North America and North America–South America was considered plausible. However, we employed some conservative constraints on the potential dispersal route between Asia and South America, as well as relevant probability calculation: the direct dispersal between the two continental regions was not allowed, and the probabilities of ancestral areas produced by multiplying two fractions (up to 1) that represent subprobabilities of Asian and South American origins, respectively, were inapplicable to the ultimate addition operations. This is because any hadrosaurine dispersal routes between the two non-adjacent areas would pass through a third continental region where the fossil evidence of relevant taxon/taxa with close affinities should be documented. Moreover, for Hadrosaurinae and its major clades, the split events were postulated to have occurred earlier than the first geological appearance of their respective subclades. The biostratigraphic information of hadrosaurine taxa used in the biogeographic analysis is based on published data documented in S2 Table. Given that the use of the geological time scale (GTS) 2012–2016 after the innovation of the ^40^Ar–^39^Ar dating technique may lead to the recalibration of absolute ages of known hadrosaurine taxa, the GTS 2009 was adopted to locate the stratigraphic ages of selected taxa in the time-calibrated cladogram.

## Supporting information

S1 Dataset Character-taxon matrix in nexus format. (NEX)

S1 Table Cranial linear measurements for the holotype and paratype of *Edmontosaurus regalis*. (PDF)

S2 Table Biostratigraphic information for hadrosaurine species used in the biogeographic analysis. (PDF)

S1 Text Character list for the cladistic analysis. (PDF)

S2 Text Probability calculation of ancestral areas for nodes within Hadrosaurinae. (PDF)
