# Supplementary material for: Supplementary cranial description of the types of Edmontosaurus regalis (Ornithischia: Hadrosauridae), with comments on the phylogenetics and biogeography of Hadrosaurinae
Source: PLoS One. 2017 Apr 6;12(4):e0175253. doi: 10.1371/journal.pone.0175253 (PMC5383305; doi:10.1371/journal.pone.0175253)
Supplement: S1 Table — (PDF) [file pone.0175253.s002.pdf]

S1 Table

Cranial linear measurements for the holotype and paratype of *Edmontosaurus regalis*

Table 1 Linear measurements (in mm) of the holotype (CMN 2288)

Element with measured attribute	Measurement
Cranium, width of skull roof across central bodies of paired postorbitals	220*
Cranium, width of skull across jugal processes of paired postorbitals	286*
Cranium, width of skull roof across quadrate cotyli of paired squamosals	195*
Cranium, total length	1060
Cranium, maximum height	585
Cranium, length of circumnarial fossa	455
Cranium, length of external naris	305
Cranium, depth of external naris	95
Cranium, width of orbit	175
Cranium, height of orbit	220
Cranium, width of dorsal region of infratemporal fenestra	20
Left quadrate, total height	420
Left jugal, total length	385
Left nasal, total length	575

*The measurement is probably influenced by either taphonomic deformation or plaster reconstruction.

Table 2 Linear measurements (in mm) of the paratype (CMN 2289)

Element with measured attribute	Measurement
Cranium, width of skull roof across central bodies of paired postorbitals	310
Cranium, width of skull across jugal processes of paired postorbitals	425
Cranium, width of skull roof across quadrate cotyli of paired squamosals	260
Cranium, width of dorsal region of infratemporal fenestra	18
Cranium, maximum width of foramen magnum	110
Cranium, length of supratemporal fenestra	170
Cranium, maximum width of supratemporal fenestra	80
Cranium, width of skull across paroccipital processes	320
Cranium, depth of central shelf of paired exoccipitals	115
Left squamosal, length of quadrate cotylus	76
Right squamosal, length of quadrate cotylus	76
Parietal, total length	212
Left frontal, maximum width	118

Right frontal, maximum width	114
Left maxilla, length along ventral margin	430
Left maxilla, total height	165
Left maxilla, length of ectopterygoid ridge	158
Left maxilla, length of dorsal surface of anteroventral process	210
Right maxilla, length along ventral margin	440
Right maxilla, total height	177*
Right maxilla, length of ectopterygoid ridge	168
Right maxilla, length of dorsal surface of anteroventral process	214
Left jugal, total length	365
Left jugal, height of anterior process	170
Left jugal, depth of anterior neck	73
Left jugal, depth of posterior neck	110
Left jugal, width of infratemporal fenestra along ventral margin of bone	48
Right jugal, total length	365
Right jugal, height of anterior process	177
Right jugal, depth of anterior neck	76
Right jugal, depth of posterior neck	120
Right jugal, width of infratemporal fenestra along ventral margin of bone	50
Left nasal, total length	570
Left nasal, maximum width of circumnarial fossa along bone	42
Right nasal, total length	570
Right nasal, maximum width of circumnarial fossa along bone	40
Left lacrimal, total length	160
Left lacrimal, width along posterior surface	60
Right lacrimal, total length	160
Right lacrimal, width along posterior surface	62
Left prefrontal, total length	185
Left prefrontal, maximum width	78
Right prefrontal, maximum width	83
Left palatine, total length	165*
Left palatine, total height	134
Right palatine, total length	170
Right palatine, total height	130
Left quadrate, total height	420
Left quadrate, width across pterygoid wing	140
Left quadrate, depth of quadratojugal notch	140
Left quadrate, width of ventral condyles	70
Left quadrate, width of dorsal head	40
Right quadrate, depth of quadratojugal notch	140
Right quadrate, width of dorsal head	28*
Left quadratojugal, total length	122
Left quadratojugal, total height	135
Right quadratojugal, total length	122

Right quadratojugal, total height	135
Left pterygoid, length of ventral part across ectopterygoid ramus	180
Left pterygoid, height across articular surface for basipterygoid process	149
Right pterygoid, length of ventral part across ectopterygoid ramus	180
Right pterygoid, height across articular surface for basipterygoid process	165*
Left dentary, total length	770
Left dentary, height across coronoid process	290
Left dentary, length of dental battery	375
Left dentary, length of apex of coronoid process	105
Left dentary, maximum width of symphysial process	100
Right dentary, total length	780
Right dentary, height across coronoid process	245*
Right dentary, length of dental battery	375
Right dentary, length of apex of coronoid process	110
Right dentary, maximum width of symphysial process	105
Left surangular, height across laterodorsal flange	43
Left surangular, height across retroarticular process	28*
Right surangular, height across laterodorsal flange	45
Right surangular, height across retroarticular process	33
Left splenial, maximum height	74*
Right splenial, maximum height	80
Dentition, width of three selected tooth crowns in middle part of left maxilla	8, 8, 8
Dentition, width of three selected tooth crowns in middle part of right maxilla	8, 8, 8
Dentition, height of three selected tooth crowns in middle part of left dentary	38, 39, 37
Dentition, width of three selected tooth crowns in middle part of left dentary	13, 13, 12
Dentition, height of three selected tooth crowns in middle part of right dentary	38, 38, 38
Dentition, width of three selected tooth crowns in middle part of right dentary	12, 13, 13

*The measurement is probably influenced by either taphonomic deformation or plaster reconstruction.
