# Supplementary material for: Supplementary cranial description of the types of Edmontosaurus regalis (Ornithischia: Hadrosauridae), with comments on the phylogenetics and biogeography of Hadrosaurinae
Source: PLoS One. 2017 Apr 6;12(4):e0175253. doi: 10.1371/journal.pone.0175253 (PMC5383305; doi:10.1371/journal.pone.0175253)
Supplement: S2 Table — (PDF) [file pone.0175253.s003.pdf]

S2 Table

Biostratigraphic information for hadrosaurine species used in the biogeographic analysis

Table 1 Published biostratigraphic data of selected hadrosaurine taxa adopted in this paper

Taxon	Stratigraphic range	Location	Landmass	Age
**Hadrosaurini**				
Hadrosaurus foulkii<br>Leidy, 1858; Prieto-Márquez et al., 2006	Woodbury Formation<br>(precise horizon unknown)	northern New Jersey, USA	North America<br>(Appalachia)	early–middle Campanian<br>80.5–78.5 Ma*<br>Gallagher, 2005; Ross and Fastovsky, 2006
**Brachylophosaurini**				
Acristavus gagslarsoni<br>Gates et al., 2011	lower part of Two Medicine Formation;<br>middle part of Wahweap Formation	northern Montana, USA;<br>southern Utah, USA	North America<br>(Laramidia)	early Campanian<br>80.7–79.7 Ma*<br>Freedman Fowler and Horner, 2015
Maiasaura peeblesorum<br>Horner and Makela, 1979	middle to upper part of Two Medicine Formation	northern Montana, USA	North America<br>(Laramidia)	middle Campanian<br>~76.7 Ma*<br>Varricchio et al., 2010
Brachylophosaurus canadensis<br>Sternberg, 1953; Cuthbertson and Holmes, 2010	middle to upper part of Judith River Formation; middle part of Oldman Formation	northern and central Montana, USA; southern Alberta, Canada	North America<br>(Laramidia)	middle Campanian<br>79.0–77.8 Ma*<br>Freedman Fowler and Horner, 2015

Probrachylophosaurus bergei Freedman Fowler and Horner, 2015	middle part of Judith River Formation	northern Montana, USA	North America (Laramidia)	early Campanian 79.8–79.5 Ma* Freedman Fowler and Horner, 2015
Wulagasaurus dongi Godefroit et al., 2008	upper part of Yuliangzi Formation	northern Heilongjiang, China	Asia	middle Maastrichtian (specific age uncertain) Markevich et al., 2009
**Kritosaurini**				
Kritosaurus navajovius Brown, 1910; Prieto-Márquez, 2014	upper part of Kirtland Formation; lower part of Cerro del Pueblo Formation	northwestern New Mexico, USA; southern Coahuila, Mexico	North America (Laramidia)	late Campanian 73.7–71.5 Ma* Eberth et al., 2004; Sullivan and Lucas, 2006
Gryposaurus latidens Horner, 1992	lower part of Two Medicine Formation	northern Montana, USA	North America (Laramidia)	early Campanian 80.0–78.0 Ma* Horner et al., 2001
Gryposaurus notabilis Lambe, 1914	lower half of Dinosaur Park Formation	southern Alberta, Canada	North America (Laramidia)	middle Campanian 76.5–75.9 Ma* Arbour et al., 2009; Mallon et al., 2012
Gryposaurus monumentensis Gates and Sampson, 2007	middle part of Kaiparowits Formation	southern Utah, USA	North America (Laramidia)	middle–late Campanian 76.1–74.8 Ma* Roberts et al., 2005
Rhinorex condrupus Gates and Scheetz, 2015	lower part of Neslen Formation	eastern Utah, USA	North America (Laramidia)	middle Campanian 75.7–74.9 Ma* Cobban et al., 2006

Secernosaurus koerneri Brett-Surman, 1979	upper part of Bajo Barreal Formation; middle part of Los Alamitos Formation	southern Chubut, Argentina; southeastern Rio Negro, Argentina	South America	late Campanian–early Maastrichtian (specific age uncertain) Prieto-Márquez and Salinas, 2010
**Edmontosaurini**				
Edmontosaurus regalis Lambe, 1917	middle part of Horseshoe Canyon Formation	southern Alberta, Canada	North America (Laramidia)	latest Campanian 72.5–71.0 Ma* Campione and Evans, 2011; Eberth et al., 2013
Edmontosaurus annectens Marsh, 1892; Campione and Evans, 2011	Lance, Hell Creek, and Frenchman formations	southeastern Montana, USA; northeastern Wyoming, USA; southwestern North Dakota, USA; northwestern South Dakota, USA; southwestern Saskatchewan, Canada	North America (Laramidia)	late Maastrichtian 66.9–65.5 Ma* Hicks et al., 2002; Lehman et al., 2006
Kerberosaurus manakini Bolotsky and Godefroit, 2004	lower to upper part of Udurchukan Formation	southern Russian Far East	Asia	early–middle Maastrichtian (specific age uncertain) Markevich and Bugdaeva, 2001; Herman et al., 2009
Shantungosaurus giganteus Hu, 1973	top of Xingezhuang Formation to middle of Hongtuya Formation	southeastern Shandong, China	Asia	middle–late Campanian (specific age uncertain) Yan et al., 2005; Liu et al., 2011
**Saurolophini**				

Lophorhothon atopus Langston, 1960	middle to upper part of Mooreville Chalk Formation (precise horizon unknown)	westcentral Alabama, USA	North America (Appalachia)	early Campanian (specific age uncertain) Langston, 1960; Kiernan, 2002
Prosaurolophus maximus Brown, 1916	upper half of Dinosaur Park Formation upper part of Two Medicine Formation	southern Alberta, Canada northern Montana, USA	North America (Laramidia)	middle–late Campanian 75.7–74.1 Ma* Mallon et al., 2012; McGarrity et al., 2013
Saurolophus osborni Brown, 1912	middle to upper part of Horseshoe Canyon Formation	southern Alberta, Canada	North America (Laramidia)	early–middle Maastrichtian 70.4–68.4 Ma* Eberth et al., 2013
Saurolophus angustirostris Rozhdestvensky, 1952	middle to upper part of Nemegt Formation (V. R. Alifanov pers. comm., 2013)	southern Bayankhongor, Mongolia; southern Govi–Altai, Mongolia; western Ömnögovi, Mongolia	Asia	early–middle Maastrichtian (specific age uncertain) Jerzykiewicz and Russell, 1991; Jerzykiewicz, 2003

*The absolute ages of designated taxa were estimated by the respective dating approaches, under the geologic time scale (GTS) established in 2009.

References

Arbour VM, Burns ME, Sissons RL (2009) A redescription of the ankylosaurid dinosaur *Dyoplosaurus acutosquameus* Parks, 1924 (Ornithischia: Ankylosauria) and a revision of the genus. Journal of Vertebrate Paleontology 29(4): 1117–1135.

Bolotsky YL, Godefroit P (2004) A new hadrosaurine dinosaur from the Late Cretaceous of Far Eastern Russia. Journal of Vertebrate Paleontology 24: 354–368.

Brett-Surman MK (1979) Phylogeny and palaeobiogeography of hadrosaurian dinosaurs. Nature 277: 560–562.

Brown B (1910) The Cretaceous Ojo Alamo beds of New Mexico with description of the new dinosaur genus *Kritosaurus*. Bulletin American Museum of Natural History 28: 267–274.

Brown B (1912) A crested dinosaur from the Edmonton Cretaceous. Bulletin American Museum of Natural History 31: 131–136.

Brown B (1916) A new crested trachodont dinosaur *Prosaurolophus maximus*. Bulletin American Museum of Natural History 35: 701–708.

Campione NE, Evans DC (2011) Cranial growth and variation in edmontosaurs (Dinosauria: Hadrosauridae): implications for latest Cretaceous megaherbivore diversity in North America. PLoS ONE 6(9): e25186.

Cobban WA, Walaszczyk I, Obradovich JD, McKinney KC (2006) A USGS zonal table for the Upper Cretaceous Middle Cenomanian–Maastrichtian of the Western Interior of the United States based on ammonites, inoceramids, and radiometric ages. U.S. Geological Survey Open-File Report 2006-1250 pp. 1–46.

Cuthbertson RS, Holmes RB (2010) The first complete description of the holotype of Brachylophosaurus canadensis Sternberg, 1953 (Dinosauria: Hadrosauridae) with comments on intraspecific variation. Zoological Journal of the Linnean Society 159: 373–397.

Eberth DA, Delgado-de Jesús CR, Lerbekmo JF, Brinkman DB, Rodríguez-de la Rosa RA, Sampson SD (2004) Cerro del Pueblo Fm (Difunta Group, Upper Cretaceous), Parras Basin, southern Coahuila, Mexico: reference sections, age, and correlation. Revista Mexicana de Ciencias Geológicas 21(3): 335–352.

Eberth DA, Evans DC, Brinkman DB, Therrien F, Tanke DH, Russell LS (2013) Dinosaur biostratigraphy of the Edmonton Group (Upper Cretaceous), Alberta, Canada: evidence for

climate influence. Canadian Journal of Earth Sciences 50(7): 701–726.

Freedman Fowler EA, Horner JR (2015) A new brachylophosaurin hadrosaur (Dinosauria: Ornithischia) with an intermediate nasal crest from the Campanian Judith River Formation of Northcentral Montana. PLoS ONE 10(11): e0141304.

Gallagher WB (2005) Recent mosasaur discoveries from New Jersey and Delaware, USA: stratigraphy, taphonomy and implications for mosasaur extinction. Netherlands Journal of Geosciences 84(3): 241–245.

Gates TA, Horner JR, Hanna RR, Nelson CR (2011) New unadorned hadrosaurine hadrosaurid (Dinosauria, Ornithopoda) from the Campanian of North America. Journal of Vertebrate Paleontology 31(4): 798–811.

Gates TA, Sampson SD (2007) A new species of *Gryposaurus* (Dinosauria: Hadrosauridae) from the late Campanian Kaiparowits Formation, southern Utah, USA. Zoological Journal of the Linnean Society 151: 351–376.

Gates TA, Scheetz R (2015) A new saurolophine hadrosaurid (Dinosauria: Ornithopoda) from the Campanian of Utah, North America. Journal of Systematic Palaeontology 13(8): 711–725.

Godefroit P, Hai SL, Yu TX, Lauters P (2008) New hadrosaurid dinosaurs from the uppermost Cretaceous of northeastern China. Acta Palaeontologica Polonica 53: 47–74.

Herman AB, Akhmetiev MA, Kodrul TM, Moiseeva MG, Iakovleva AI (2009) Flora development in Northeastern Asia and Northern Alaska during the Cretaceous-Paleogene transitional epoch. Stratigraphy and Geological Correlation 17(1): 79–97.

Hicks JF, Johnson KR, Obradovich JD, Tauxe L, Clark D (2002) Magnetostratigraphy and geochronology of the Hell Creek and basal Fort Union Formations of southwestern North Dakota and a recalibration of the age of the Cretaceous-Tertiary boundary. Geological Society of America Special Papers 361: 35–55.

Horner JR (1992) Cranial morphology of *Prosaurolophus* (Ornithischia: Hadrosauridae) with descriptions of two new hadrosaurid species and an evaluation of hadrosaurid phylogenetic relationships. Museum of the Rockies, Occasional Paper 2: 1–119.

Horner JR, Makela R (1979) Nest of juveniles provides evidence of family structure among dinosaurs. Nature 282: 296–298.

Horner JR, Schmitt JG, Jackson F, Hanna R (2001) Bones and rocks of the Upper Cretaceous Two

Medicine – Judith River clastic wedge complex, Montana. Museum of the Rockies, Occasional Paper 3: 1–14.

Hu CZ (1973) A new hadrosaur from the Cretaceous of Zhucheng, Shantung. Acta Geologica Sinica 2: 179–206.

Jerzykiewicz T (2003) Lithostratigraphy and sedimentary settings of the Cretaceous dinosaur beds of Mongolia. In: Benton MJ, Shishkin MA, Unwin DM, Kurochkin EN, eds. The Age of Dinosaurs in Russia and Mongolia. Cambridge: Cambridge University Press. pp. 279–296.

Jerzykiewicz T, Russell DA (1991) Late Mesozoic stratigraphy and vertebrates of the Gobi Basin. Cretaceous Research 12: 345–377.

Kiernan CR (2002) Stratigraphic distribution and habitat segregation of mosasaurs in the Upper Cretaceous of western and central Alabama, with an historical review of Alabama mosasaur discoveries. Journal of Vertebrate Paleontology 22(1): 91–103.

Lambe LM (1914) On *Gryposaurus notabilis*, a new genus and species of trachodont dinosaur from the Belly River Formation of Alberta. Ottawa Naturalist 27: 145–155.

Lambe LM (1917) A new genus and species of crestless hadrosaur from the Edmonton Formation of Alberta. Ottawa Naturalist 31: 65–73.

Langston WD (1960) The vertebrate fauna of the Selma Formation of Alabama, Part VI: the dinosaurs. Fieldiana Geology Memoirs 3: 313–363.

Lehman TM, Mcdowell FW, Connelly JN (2006) First isotopic (U-Pb) age for the Late Cretaceous Alamosaurus vertebrate fauna of West Texas, and its significance as a link between two faunal provinces. Journal of Vertebrate Paleontology 26(4): 922–928.

Leidy J (1858) *Hadrosaurus foulkii*, a new saurian from the Cretaceous of New Jersey, related to Iguanodon. Proceedings of the Academy of Natural Sciences of Philadelphia 10: 213–218.

Liu YQ, Kuang HW, Peng N, Xu H, Liu YX (2011) Sedimentary facies of dinosaur trackways and bonebeds in the Cretaceous Jiaolai Basin of Shandong Province and their paleogeographical implications. Earth Science Frontiers 18(4): 9–24.

Mallon JC, Evans DC, Ryan MJ, Anderson JS (2012) Megaherbivorous dinosaur turnover in the Dinosaur Park Formation (upper Campanian) of Alberta, Canada. Palaeogeography, Palaeoclimatology, Palaeoecology 350: 124–138.

Markevich VS, Bugdaeva EV (2001) Correlation of the Upper Cretaceous and Palaeogene

plant-bearing deposits of the Russian Far East. In: Bugdaeva E V ed. Flora and dinosaurs at the Cretaceous–Paleogene boundary of Zeya-Bureya Basin. Vladivostok: Dalnauka. pp. 79–96.

Markevich VS, Bugdaeva EV, Sun G (2009) Palynoflora of Wulaga dinosaur site in Jiayin (Zeya-Bureya Basin, China). Global Geology 12(3): 117–121.

Marsh OC (1892) Notice of new reptiles from the Laramie Formation. American Journal of Science (third series) 257: 449–453.

McGarrity CT, Campione NE, Evans DC (2013) Cranial anatomy and variation in *Prosaurolophus maximus* (Dinosauria: Hadrosauridae). Zoological Journal of the Linnean Society 167(4): 531–568.

Prieto-Márquez A (2014) Skeletal morphology of *Kritosaurus navajovius* (Dinosauria: Hadrosauridae) from the Late Cretaceous of the North American south-west, with an evaluation of the phylogenetic systematics and biogeography of Kritosaurini. Journal of Systematic Palaeontology 12(2): 133–175.

Prieto-Márquez A, Salinas GC (2010) A re-evaluation of *Secernosaurus koerneri* and *Kritosaurus australis* (Dinosauria, Hadrosauridae) from the Late Cretaceous of Argentina. Journal of Vertebrate Paleontology 30(3): 813–837.

Prieto-Márquez A, Weishampel DB, Horner JR (2006) The hadrosaurid dinosaur *Hadrosaurus foulkii* from the Campanian of the East coast of North America, with a review of the genus. Acta Palaeontologica Polonica 51: 77–98.

Roberts EM, Deino AL, Chan MA (2005) $^{40}Ar/^{39}Ar$ age of the Kaiparowits Formation, southern Utah, and correlation of contemporaneous Campanian strata and vertebrate faunas along the margin of the Western Interior Basin. Cretaceous Research 26: 307–318.

Ross MR, Fastovsky DE (2006) Trans-Atlantic correlations of Upper Cretaceous marine sediments: the Mid-Atlantic (USA) and Maastricht (Netherlands) regions. Northeastern Geology and Environmental Sciences 28(1): 34–44.

Rozhdestvensky AK (1952) A new representative of the duck-billed dinosaurs from the Upper Cretaceous deposits of Mongolia. Dokladi Akademii Nauk USSR 86: 405–408.

Sternberg CM (1953) A new hadrosaur from the Oldman Formation of Alberta: discussion of nomenclature. Bulletin of the National Museum of Canada 128: 275–286.

Sullivan RM, Lucas SG (2006) The Kirtlandian land-vertebrate “age” – faunal composition,

temporal position and biostratigraphic correlation in the nonmarine Upper Cretaceous of western North America. New Mexico Museum of Natural History and Science Bulletin 35: 7–29.

Varricchio DJ, Koeberl C, Raven RF, Wolbach WS, Elsik WC, Miggins DP (2010) Tracing the Manson impact event across the Western Interior Cretaceous Seaway. Geological Society of America Special Paper 465: 269–299.

Yan J, Chen JF, Xie Z, Gao TS, Foland KA, Zhang XD, et al. (2005) Studies on petrology and geochemistry of the Late Cretaceous basalts and mantlederived xenoliths from eastern Shandong. Acta Petrologica Sinica 21(1): 99–112.
