# Supplementary material for: Supplementary cranial description of the types of Edmontosaurus regalis (Ornithischia: Hadrosauridae), with comments on the phylogenetics and biogeography of Hadrosaurinae
Source: PLoS One. 2017 Apr 6;12(4):e0175253. doi: 10.1371/journal.pone.0175253 (PMC5383305; doi:10.1371/journal.pone.0175253)
Supplement: S1 Text — (PDF) [file pone.0175253.s004.pdf]

S1 Text

Character list for the cladistic analysis

Dentition

1. Maximum number of dental alveoli (tooth positions) in the dentary dental battery (modified from Horner et al., 2004 character 1): (0) 30 or fewer; (1) 31 to 45; (2) more than 45.

2. Number of dentary teeth per centimeter of the dental battery (modified from Prieto-Márquez and Wagner, 2009 character 2): (0) less than 0.7 tooth/cm for the dental battery; (1) 0.7 or more teeth/cm for the dental battery.

3. Maximum number of teeth per alveolus of the dentary in adults (modified from Horner et al., 2004 character 2): (0) two; (1) three; (2) four to six; (3) seven or more.

4. Maximum number of functional teeth on the dentary occlusal surface (modified from Prieto-Márquez and Wagner, 2009 character 4): (0) only one functional tooth; (1) one functional tooth on the anterior and posterior portions of the occlusal surface, and up to two near the middle part of the dental battery; (2) two functional tooth on the anterior and posterior portions of the occlusal surface, and up to three near the middle part of the dental battery; (3) three functional teeth throughout most of the occlusal surface (in some cases, with the sporadic presence of four functional teeth), decreasing to two at the anterior and posterior ends of the dental battery.

5. Height/width ratio of the dentary tooth crown in lingual view (corresponding with Prieto-Márquez and Wagner, 2009 character 5): (0) ratio less than 1.95; (1) ratio from 1.95 to 2.70; (2) ratio greater than 2.70 and up to 3.30; (3) ratio greater than 3.30.

6. Maximum number of ridges on the enameled surface of the dentary tooth crown in lingual view (modified from Horner et al., 2004 character 6): (0) five or more in total (a pronounced primary ridge ranging from the dorsal to the ventral end of the tooth crown, a slightly short secondary ridge and several less developed subsidiary ridges); (1) two to four in total (a pronounced primary ridge, a slightly short secondary ridge and no more than two less developed subsidiary ridges); (2) two or three in total (a pronounced primary ridge and one or two less developed subsidiary ridges); (3) only one primary ridge.

7. Position of the primary ridge on the enameled surface of the dentary tooth crown

(corresponding with Prieto-Márquez and Wagner, 2009 character 7): (0) distal to the midline of the tooth crown; (1) median for most teeth and maybe distal to the midline of the tooth crown for some teeth.

8. Shape of the primary ridge of the dentary tooth crown (modified from Godefroit et al., 2000 character 23): (0) straight in all teeth; (1) straight for some teeth and slightly curved for others.

9. Angle between crown and root of dentary teeth (modified from Godefroit et al., 2000 character 24): (0) not more than 110º; (1) more than 110º and at most 130º; (2) more than 130º.

10. General profile of the root of dentary teeth in anterior view (corresponding with Xing et al., 2012 character 10): (0) curved labially; (1) basically straight and slightly inclined lingually.

11. General shape of the marginal denticles on the lingual enameled crowns of dentary teeth (modified from Prieto-Márquez and Wagner, 2009 character 10): (0) bamboo leaf-like denticles along the dorsal half of the margin of the crown; (1) slightly big mammilliform denticles along the dorsal half of the margin of the crown; (2) relatively small mammilliform denticles along the dorsal half of the margin of the crown.

12. Composition of the marginal denticles of dentary teeth (modified from Prieto-Márquez and Wagner, 2009 character 11): (0) each denticle consisting of several small and rounded knobs; (1) each denticle consisting of one rounded knob.

13. Shape of septa that separate the dentary alveoli (corresponding with Prieto-Márquez and Wagner, 2009 character 13): (0) thick and stick-like septa; (1) thin and sheet-like septa.

14. Morphology of dental alveoli in the dentary (modified from Prieto-Márquez and Wagner, 2009 character 14): (0) short and tongue-like alveoli; (1) long, narrow and upright alveoli which is parallel with each other.

15. Distribution of the enamel of dentary crowns (corresponding with Norman, 2002 character 30): (0) presence of a thin veneer labially, thick lingually; (1) only present lingually.

16. Maximum number of dental alveoli (tooth positions) in the maxillary dental battery (modified from Horner et al., 2004 character 1): (0) 32 or fewer; (1) 33 to 45; (2) more than 45.

17. Number of teeth per centimeter of the maxillary dental battery in adults (corresponding with Prieto-Márquez and Wagner, 2009 character 17): (0) less than 1.25 teeth/cm of dental battery; (1) 1.25 or more teeth/cm of dental battery.

18. Comparison between the number of maxillary alveoli (N_m) and dentary ones (N_d) in adult

specimens (corresponding with Prieto-Márquez and Wagner, 2009 character 18): (0) $N_m \leq N_d$; (1) $N_m > N_d$.

19. Maximum number of functional teeth exposed on the maxillary occlusal surface (modified from Horner et al., 2004 character 3): (0) only one functional tooth; (1) one functional tooth for most of the occlusal surface, and up to two casually near the middle part of the maxilla; (2) two functional teeth throughout most of the occlusal surface, decreasing to one at the anterior and posterior ends of the maxilla.

20. Maximum number of ridges on the enameled surface of the maxillary tooth crown in labial view (modified from Horner et al., 2004 character 7): (0) four or more in total (a pronounced primary ridge extending longitudinally through the tooth crown and three or more less developed subsidiary ridges); (1) one to three in total (a pronounced primary ridge and at most two less developed subsidiary ridge).

21. Position of the primary ridge on the enameled surface of the maxillary tooth crown (modified from You et al., 2003a character 36): (0) distal to the midline of the tooth crown; (1) median for most teeth and maybe distal to the midline of the tooth crown for the minority of teeth.

22. Shape of the primary ridge of the maxillary tooth crown (modified from Godefroit et al., 2000 character 23): (0) straight for all teeth; (1) straight for some teeth and slightly sinuous for others.

23. General shape of marginal denticles on the enameled labial surfaces of the maxillary tooth crowns (modified from Prieto-Márquez and Wagner, 2009 character 23): (0) saw-toothed denticles along the ventral half of the crown margin; (1) slightly big mammilliform denticles along the ventral half of the crown margin; (2) relatively small mammilliform denticles along the ventral half of the crown margin.

Mandible

Predentary

24. Ratio between the maximum mediolateral width and maximum anteroposterior length of the predentary (corresponding with Prieto-Márquez and Wagner, 2009 character 24): (0) less than 1.20; (1) 1.20 to 1.75; (2) more than 1.75.

25. Ratio between the dorsoventral depth of the anterior region and the length of the lateral process of the predentary (corresponding with Prieto-Márquez and Wagner, 2009 character 25): (0)

more than 0.38; (1) 0.38 or less.

26. Shape of the denticles of the predentary anterior margin (modified from Horner et al., 2004 character 13): (0) triangular; (1) trapeziform.

27. Angle between the anteroventral surface of the anterior end and the dorsal surface of the lateral process of the predentary in lateral view (corresponding with Prieto-Márquez and Wagner, 2009 character 26): (0) 75º or greater; (1) 56º to 74º; (2) 40º to 55º; (3) less than 40º.

28. Spacing between two adjacent denticles of the predentary in adults (corresponding with Prieto-Márquez and Wagner, 2009 character 28): (0) the spacing between two adjacent denticles is longer than or equal to the width of each denticle; (1) the spacing between two adjacent denticles is shorter than the width of each denticle but longer than 25% of its width; (2) no spacing between two adjacent denticles, tightly connecting with each other.

29. Number of the denticles on the anterior surface of half the predentary in adults, not including the middle one in the count (modified from Prieto-Márquez and Wagner, 2009 character 29): (0) up to five; (1) six; (2) more than six.

30. Position of the predentary denticles (corresponding with Prieto-Márquez and Wagner, 2009 character 30): (0) denticles extending to the lateral process of the predentary; (1) denticles only limited to the anterior surface of the predentary.

31. Shape of the joint between the anterior part and the lateral process of the predentary (modified from Horner et al., 2004 character 13): (0) smoothly rounded, forming a bowed predentary in dorsal view; (1) subsquare; (2) almost square, with the presence of a remarkable anterolateral projection.

32. Lateral shelf of the predentary lateral process (corresponding with Xing et al., 2012 character 32): (0) absent; (1) present.

33. Morphology of the lateral shelf along the predentary lateral process (modified from Prieto-Márquez and Wagner, 2009 character 32): (0) absent, presence of an anteroposteriorly short and shallow depression at the posterior end of the lateral process; (1) presence of a short and less developed lateral shelf along the posterolateral margin of the lateral process; (2) presence of a short, well incised and mediolaterally broad lateral shelf along the anterolateral margin of the lateral process; (3) presence of a long and well incised lateral shelf throughout the lateral margin of the lateral process, forming a mediolaterally broad plane; (4) presence of a long, well incised

and mediolaterally narrow lateral shelf throughout the lateral margin of the lateral process.

34. Strong posterior extension of the posterolateral corner of the predentary lateral process (corresponding with Prieto-Márquez and Wagner, 2009 character 33): (0) absent, the lingual margin of the lateral process is slightly longer or nearly equal to the labial margin of the lateral process; (1) present, the labial margin of the lateral process is much longer than the lingual margin of the lateral process.

35. Development of the dorsal median process of the predentary (modified from Prieto-Márquez and Wagner, 2009 character 34): (0) presence of a faint dorsal median process that is slightly protrudent posterodorsally; (1) presence of a well developed dorsal median process without a prominent ridge; (2) presence of a well developed dorsal median process with a prominent ridge on its lingual side, extending posteriorly from the posterior margin of the predentary anterior region.

36. Comparison between the anteroposterior length of the ventral median process (L) and the dorsoventral depth (D) of the anterior end of the predentary (modified from Prieto-Márquez and Wagner, 2009 character 35): (0) $L > D$; (1) $L \leq D$.

37. Anteroposterior length of the split of the bilobate ventral median process relative to the undivided portion in ventral view (modified from Prieto-Márquez and Wagner, 2009 character 36): (0) short split and relatively long undivided portion; (1) long split and relatively short undivided portion.

Dentary

38. Ratio between the length of the edentulous slope anterior to the dentary dental battery (not including the anteriormost portion which contacts the predentary) and the horizontal distance between the first tooth position and the posterior end of the coronoid process (corresponding with Prieto-Márquez and Wagner, 2009 character 37): (0) less than 0.20; (1) 0.20 to 0.31; (2) greater than 0.31 and up to 0.45; (3) greater than 0.45.

39. Angle between the edentulous slope of the dentary anterior portion and the level (corresponding with Prieto-Márquez and Wagner, 2009 character 38): (0) less than 150º; (1) 150º or greater.

40. Angle between the slope of the dentary anterior region that contacts the predentary and the horizontal (modified from Prieto-Márquez and Wagner, 2009 character 39): (0) greater than 130º;

(1) 115º to 130º; (2) less than 115º.

41. Degree of the downward deflection of the anterior part of the dentary that contacts the predentary, measured as the angle between the ventral margin of the anterior part of the dentary ramus and the dorsolateral margin of the posterior part of the ramus (modified from Prieto-Márquez and Wagner, 2009 character 40): (0) faintly deflected ventrally, with the angle less than 17º; (1) moderately deflected ventrally, with the angle between 17º to 25º; (2) markedly deflected ventrally, with the angle greater than 25º.

42. Ratio between the horizontal distance from the posterior margin of the coronoid process to the posteriormost end of the deflected ventral margin of the dentary and horizontal distance from the posterior margin of the coronoid process to the first alveolus (corresponding with Prieto-Márquez and Wagner, 2009 character 41): (0) greater than 0.78; (1) 0.66 to 0.78; (2) less than 0.66.

43. Ratio between the maximum mediolateral width of the dentary symphysial region and the minimum breadth of the dentary posterior to the dentary symphysial region in dorsal view (modified from Prieto-Márquez and Wagner, 2009 character 42): (0) up to 1.65; (1) greater than 1.65 and up to 2.60; (2) more than 2.60.

44. Angle between the medial surface of dentary symphysis and the lateral surface of the anterior region of the dentary in ventral view (modified from Prieto-Márquez and Wagner, 2009 character 43): (0) greater than 15º; (1) up to 15º.

45. General profile of the dorsal margin of the dentary anterior region that contacts the predentary in medial or lateral view (corresponding with Prieto-Márquez and Wagner, 2009 character 45): (0) a smooth, gradually descending dorsal margin in the anterodorsal region of the dentary; (1) a relatively steep dorsal margin, forming a prominent depression in the dentary symphysial region.

46. Degree of the lingual curvature of the dentary symphysial region in anterior view (corresponding with Prieto-Márquez and Wagner, 2009 character 44): (0) markedly curved lingually, forming a nearly 90º lateroventral profile in anterior view; (1) gently curved lingually, forming a sloping, wide arcuate lateroventral profile in anterior view.

47. General profile of the dentary ventral margin below the coronoid process in lateral view (modified from Prieto-Márquez and Wagner, 2009 character 46): (0) a slightly bowed ventral

margin below the coronoid process; (1) a well developed bowed ventral margin below the coronoid process.

48. Coronoid process (modified from Godefroit et al., 2008 character 39): (0) vertical to the dorsal margin of the dentary or inclined posteriorly; (1) slightly inclined anteriorly, with an angle between 70º and 85º; (2) obviously inclined anteriorly, with an angle less than 70º.

49. Shape of the apex of the coronoid process in adults (modified from Horner et al., 2004 character 17): (0) only slightly expanded anteriorly, with less developed anterior and posterior margins; (1) markedly expanded anteroposteriorly, with well developed anterior and posterior margins; (2) markedly expanded anteroposteriorly, with well developed anterior and posterior margins, bearing a more pronounced posterior margin.

50. Medial wall of the triangular depression on the posterior side of the coronoid process in posterior view (corresponding with Xing et al., 2014 character 50): (0) not extending to the base of the dorsal apex, whose width is about one-quarter of the maximum mediolateral width of the apex; (1) extending to the base of the dorsal apex, whose width is about one-third of the maximum mediolateral width of the apex.

51. A sharp projection on the posterodorsal surface of the coronoid process of the dentary (corresponding with Prieto-Márquez and Wagner, 2009 character 49): (0) absent; (1) present.

52. Development of the ridge on the medial side of the coronoid process of the dentary (corresponding with Prieto-Márquez and Wagner, 2009 character 50): (0) presence of a faint, projecting medially ridge on the medial surface of the coronoid process; (1) presence of a well developed and expanded posteriorly ridge, forming the mediodorsal boundary of a depressed facet which contacts the anterodorsal process of the surangular.

53. Angle between the lateral surface of the dentary portion anterior to the coronoid process and that of the dentary portion anteroventral to the coronoid process in dorsal or ventral view (corresponding with Prieto-Márquez and Wagner, 2009 character 51): (0) the lateral surface of the dentary portion ventral to the coronoid process is only slightly expanded laterally, with an angle greater than 165º; (1) the lateral surface of the dentary portion ventral to the coronoid process is remarkably expanded laterally, with an angle up to 165º.

54. Orientation of the longitudinal axis of the dentary occlusal surface relative to the lateral margin of the dentary in dorsal view (corresponding with Prieto-Márquez and Wagner, 2009

character 52): (0) presence of a relatively inclined longitudinal axis, forming an angle of about 20º with the lateral margin of the dentary; (1) presence of a longitudinal axis running parallel with the lateral margin of the dentary.

55. Lingual curvature of the longitudinal axis of the dentary occlusal surface in dorsal view (corresponding with Prieto-Márquez and Wagner, 2009 character 53): (0) present; (1) absent.

56. Position of the coronoid process relative to the dentary dental battery (modified from Horner et al., 2004 character 10): (0) the posterior margin of the coronoid process is located posterior to the posterior end of the dental battery; (1) the posterior margin of the coronoid process is overlapped with the posterior end of the dental battery; (2) the posterior margin of the coronoid process is located anterior to the posterior end of the dental battery.

57. Degree of separation between the dentary dental battery and the coronoid process (corresponding with Prieto-Márquez and Wagner, 2009 character 55): (0) the coronoid process is not obviously separate from the dentary dental battery; (1) the coronoid process is obviously separate from the dentary dental battery, with the presence of a depressed buccal shelf separating the base of coronoid process from the dental battery.

58. Anterodorsal inclination of the long axis of the dentary tooth row relative to the ventral margin of the middle part of the dentary ramus (corresponding with Wang et al., 2015 character 58): (0) present; (1) absent, the long axis of the tooth row is either generally parallel with the ventral margin of the middle region of the dentary ramus or slightly anteroventrally inclined relative to this margin.

Surangular

59. Surangular foramen (corresponding with Norman, 2002 character 27): (0) present; (1) absent.

60. Surangular accessory foramen on the anterolateral surface of the surangular (corresponding with Kobayashi and Azuma, 1999 character 15): (0) present; (1) absent.

61. Contact surface of the surangular anterodorsal process for the coronoid process of the dentary (modified from Xing et al., 2012 character 60): (0) the anterior half of the anterolateral surface of the anterodorsal process; (1) most of the anterolateral surface of the anterodorsal process.

62. Participation of the surangular in the ventral side of the mandibular posterior part (modified from Prieto-Márquez and Wagner, 2009 character 59): (0) absent, surangular only participating in the lateral side of the mandibular posterior part; (1) present, surangular facing more laterally than

ventrally; (2) present, surangular facing more ventrally than laterally.

63. Strong upward curvature of the retroarticular process of the surangular (corresponding with Prieto-Márquez and Wagner, 2009 character 60): (0) absent; (1) present.

64. Lateral profile of the surangular posteroventral corner ventral to the retroarticular process (corresponding with Xing et al., 2014 character 65): (0) broadly arcuate; (1) a nearly 90º angle.

65. General shape of the retroarticular process of the surangular (corresponding with Xing et al., 2014 character 66): (0) relatively robust in lateral view, forming a round, blunt posterodorsal extremity; (1) subtriangular in lateral view, forming a posterodorsally tapering extremity.

66. Angle between the ventromedial margin of the anterior part of the surangular and that of the retroarticular process (modified from Prieto-Márquez and Wagner, 2009 character 61): (0) 150º or greater; (1) less than 150º.

Angular

67. Position of the angular of the mandible (corresponding with Weishampel et al., 1993 character 26): (0) the angular exposed in lateral view, with a ventral and slightly lateral position; (1) the angular not exposed in lateral view, with a medial position.

Coronoid

68. Coronoid bone (corresponding with Wagner, 2001 character 28): (0) present; (1) absent.

Prearticular

69. Prearticular bone (corresponding with Prieto-Márquez and Wagner, 2009 character 64): (0) present; (1) absent.

Splenial

70. Position of the splenial relative to the dentary dental battery in medial view (corresponding with Xing et al., 2012 character 68): (0) located ventral to the dental battery; (1) located posterior to the dental battery.

Other Viscerocranial Bones

Premaxilla

71. Shape of the premaxillary oral margin in dorsal view (corresponding with Horner et al., 2004 character 22): (0) horseshoe-shaped, forming a continuous semicircle that is abruptly constricted behind the oral margin; (1) broadly arcuate in the oral margin and gradually constricted to the postoral region.

72. Transverse expansion of the premaxilla in the oral region (ratio between the maximum width of the premaxilla and the minimum width of this bone at the posterior contractive region) (modified from Prieto-Márquez and Wagner, 2009 character 65): (0) faintly expanded, with the ratio less than 1.70; (1) moderately expanded, with the ratio between 1.70 and 2.00; (2) markedly expanded, with the ratio greater than 2.00.

73. Degree of the downward deflection of the anterior end of the premaxilla relative to the occlusal surface of the dentition (corresponding with Norman, 2002 character 2): (0) slightly deflected ventrally from the occlusal surface; (1) strongly deflected ventrally from the occlusal surface, almost covering the predentary in lateral view.

74. Morphology of the anterior portion of the premaxillary oral margin (modified from Prieto-Márquez and Wagner, 2009 character 67): (0) presence of a relatively narrow transversely, dorsoventrally thick, slightly deflected ventrally oral margin anterior to the circumnarial fossa; (1) presence of a moderately expanded transversely, dorsoventrally thick, strongly deflected ventrally oral margin that is slightly elevated; (2) presence of a moderately expanded transversely, dorsoventrally thin, strongly deflected ventrally oral margin, forming a smooth, gentle slope from the circumnarial fossa; (3) presence of a moderately expanded transversely, dorsoventrally thick, strongly deflected ventrally oral margin that is slightly dorsally recurved and located anterior to the premaxillary accessory narial fossa; (4) presence of a moderately expanded transversely, dorsoventrally thick, strongly deflected ventrally oral margin that is lip-shaped and markedly reflected posterodorsally.

75. Shape of the anterolateral corner of the premaxillary oral margin (corresponding with Prieto-Márquez and Wagner, 2009 character 68): (0) rounded; (1) triangular.

76. Premaxillary foramen in the anterior region of the external naris that opens onto the palate (corresponding with Horner et al., 2004 character 23): (0) absent; (1) present.

77. Premaxillary accessory foramen entering into the premaxillary accessory narial fossa, located anterior to the premaxillary foramen (corresponding with Horner et al., 2004 character 24): (0) absent; (1) present, sharing a common chamber with the premaxillary foramen.

78. Slightly elevated premaxillary accessory narial fossa (outer narial fossa) in the anterior region of the circumnarial fossa (corresponding with Horner et al., 2004 character 26): (0) absent; (1) present.

79. Premaxillary oral margin with a “double layer” morphology consisting of a dorsal denticle-bearing layer and a ventral layer which is offset slightly from the oral margin and separated from the denticular layer by a deep sulcus bearing vascular foramina (corresponding with Horner et al., 2004 character 25): (0) absent; (1) present.

80. At least one oval foramen on the anteromedial surface of the premaxillary lip (corresponding with Prieto-Márquez, 2010 character 287): (0) absent; (1) present.

81. Additional deep fossa located lateral to the premaxillary accessory narial fossa (corresponding with Prieto-Márquez and Wagner, 2009 character 73): (0) absent; (1) present.

82. Structure of the external naris anterior to the orbit (modified from Horner et al., 2004 character 27): (0) the external naris consists of the premaxilla and the nasal, and the premaxillary posterodorsal process does not meet posteroventral process posterior to the external naris; (1) the external naris only consists of the premaxilla, and premaxillary posterodorsal and posteroventral processes elongate and meet posterior to the external naris, forming the posterior margin of the external naris and excluding the nasal.

83. Development of the premaxillary posterodorsal process in adults (modified from Prieto-Márquez and Wagner, 2009 character 74): (0) relatively short, forming the dorsal margin of the external naris with the nasal; (1) moderately elongate posterodorsally, forming the anterior part of the hollow supracranial crest; (2) markedly elongate posterodorsally, forming the anterior and posterodorsal portions of the hollow supracranial crest; (3) markedly elongate posterodorsally, forming the anterior portion and most of the posterior portion of the hollow supracranial crest.

84. Development of the premaxillary posteroventral process in adults (modified from Prieto-Márquez and Wagner, 2009 character 76): (0) relatively short, the posterior end of the premaxillary posteroventral process is located anteroventral to the prefrontal; (1) moderately elongate posterodorsally, the posterior end of premaxillary posteroventral process is located anteromedial to the prefrontal; (2) strongly elongate posterodorsally, the posterior end of premaxillary posteroventral process is located dorsal or posterodorsal to the prefrontal, forming the anteroventral part of the hollow supracranial crest.

85. Premaxillary posteroventral process contacting with the nasal (corresponding with Xing et al., 2012 character 82): (0) present; (1) absent.

86. Vertical groove on the posteroventral process of the premaxilla, located anterior to the dorsal

process of the maxilla (corresponding with Evans and Reisz, 2007 character 5): (0) absent; (1) present.

87. Morphology of the posterior region of the premaxillary posteroventral process in adults (modified from Prieto-Márquez and Wagner, 2009 character 77): (0) mediolaterally compressed, arrowhead-shaped and anterior to the orbit; (1) arrowhead-shaped, anteroposteriorly expanded and dorsal to the orbit; (2) triangular, dorsoventrally expanded and dorsal to the orbit.

88. Bone posterior to the premaxillary posteroventral process (corresponding with Xing et al., 2012 character 85): (0) lacrimal; (1) nasal.

89. Premaxillary posterodorsal process with an accessory anteroventral flange that overlaps the lateral surface of the nasal in the anterior region of the crest (corresponding with Evans and Reisz, 2007 character 18): (0) absent; (1) present.

90. Shape of the external naris (modified from Evans and Reisz, 2007 character 4): (0) spindle-shaped or elliptical; (1) triangular, the posterodorsal and posteroventral margins of the external naris gradually converge posterodorsally in the posterior region; (2) relatively short and teardrop-shaped, the posterodorsal and posteroventral margins of the external naris abruptly converge posterodorsally in the posterior region.

91. Ratio between the anteroposterior length and the maximum mediolateral width of the external naris of derived lambeosaurine dinosaurs in dorsolateral view (modified from Prieto-Márquez and Wagner, 2009 character 81): (0) less than 4; (1) 4 or greater.

92. Deflection angle of the dorsal margin of the premaxillary posterodorsal process anterior to the lacrimal in the adults of lambeosaurines (modified from Prieto-Márquez and Wagner, 2009 character 194): (0) greater than 170º; (1) 100º to 170º; (2) less than 100º.

93. A well developed dorsolateral flange which is sheet-shaped in the middle of the mediolaterally compressed premaxillary posteroventral process in non-lambeosaurine iguanodontians (corresponding with Prieto-Márquez and Wagner, 2009 character 82): (0) absent; (1) present.

Maxilla

94. Anterodorsal process of the maxilla (corresponding with Horner et al., 2004 character 42): (0) presence of the maxillary anterodorsal process extending to the region medial to the premaxillary posteroventral process and forming part of ventral margin of the external naris; (1) absence of the

maxillary anterodorsal process, the anterodorsal portion of the maxilla forming a sloping shelf that underlies the premaxilla.

95. Lateral exposure of the anterodorsal process of the maxilla (corresponding with Gates and Sampson, 2007 character 45): (0) absent, the anterodorsal process cannot be observed through the external naris in lateral view; (1) present, the anterodorsal process can be seen through the external naris in lateral view.

96. Anterior extension of the anterodorsal process of the maxilla (corresponding with Xing et al., 2014 character 97): (0) the anterodorsal process is relatively truncated and low, and does not exceed the anteriormost end of the anteroventral process; (1) the anterodorsal process is elongated and elevated, and exceeds the anteriormost end of the anteroventral process.

97. Strongly downward curvature of the anteroventral process of the maxilla (corresponding with Prieto-Márquez and Wagner, 2009 character 94): (0) present; (1) absent.

98. Angle between the dorsal margin of the maxillary anteroventral process and the ventral margin of the maxillary anterior portion where the dental battery occurs (modified from Prieto-Márquez and Wagner, 2009 character 95): (0) less than 25º; (1) 25º to 40º; (2) greater than 40º.

99. General shape of the maxillary lateral surface anteroventral to the jugal sutural surface (modified from Prieto-Márquez and Wagner, 2009 character 96): (0) trapeziform or subrectangular; (1) triangular and anteroposteriorly shortened.

100. Ratio between the distance from the apex of the maxillary lateral surface located anteroventral to the jugal contact surface to the anteriormost end of the maxilla and the anteroposterior length of the maxilla (corresponding with Prieto-Márquez and Wagner, 2009 character 98): (0) more than 0.57; (1) 0.47 to 0.57; (2) 0.35 to 0.46; (3) less than 0.35.

101. Location of the apex of the adult maxilla in lateral view (corresponding with Horner et al., 2004 character 47): (0) markedly posterior to the midline of the maxilla; (1) at or anterior to the midline of the maxilla.

102. Shape of the dorsal ramus of the maxilla in lateral view (modified from Horner et al., 2004 character 48): (0) triangular, with the width greater than the height, bearing a slightly round apex; (1) triangular, with the height greater than the width, bearing a peaked and posteriorly inclined apex.

103. Morphology of the jugal contact surface in the dorsolateral region of the maxilla (modified from Prieto-Márquez and Wagner, 2009 character 100): (0) presence of a laterally offset finger-shaped jugal contact surface located at the anterior two-thirds of the maxilla; (1) presence of a low, dorsolaterally facing subquadrangular jugal contact surface located anterodorsal to the ectopterygoid shelf; (2) presence of an irregular diamond-shaped jugal contact surface at the midline of the maxilla, bearing a dorsolaterally facing ventral half which is slightly inclined posteriorly and located anterior to the ectopterygoid shelf in lateral view; (3) presence of a regular diamond-shaped jugal contact surface at the midline of the maxilla, bearing a dorsolaterally facing ventral half anterior to the ectopterygoid shelf; (4) presence of a dorsally elevated subrectangular jugal contact surface, bearing a dorsolaterally facing ventral half anterodorsal to the ectopterygoid shelf.

104. Arrangement of maxillary foramina anteroventral to the jugal contact surface (corresponding with Prieto-Márquez and Wagner, 2009 character 101): (0) the maxillary foramina are arranged anteroposteriorly and scattered on the lateral side of the maxilla; (1) the maxillary foramina are arranged anterodorsally-posteroventrally in a row.

105. Number of the maxillary foramina anteroventral to the jugal contact facet excluding the large anterior foramen (modified from Prieto-Márquez and Wagner, 2009 character 102): (0) seven or more; (1) at most six.

106. One or two large maxillary foramina immediately adjacent to the ventral extremity of the jugal contact surface of the maxilla (corresponding with Xing et al., 2014 character 107): (0) absent; (1) present.

107. Location of the large anterior maxillary foramen (modified from Evans and Reisz, 2007 character 22): (0) located on the anterior half of the maxillary anterolateral surface and exposed in lateral view; (1) located on the dorsal half of the maxillary anterolateral surface and exposed in lateral view; (2) located on the maxillary anterodorsal surface along the maxilla-premaxilla contact and not exposed in lateral view.

108. Lateral exposure of the antorbital fenestra (corresponding with You et al., 2003a character 4): (0) present; (1) absent.

109. Lateral exposure of the maxilla-lacrimal contact (corresponding with Evans and Reisz, 2007 character 23): (0) present in lateral view; (1) absent in lateral view and laterally covered by the

jugal-premaxilla contact.

110. Ratio between the vertical distance from the apex of the maxillary dorsal process to the maxillary ventral margin and the maximum length of the maxillary ventral margin (modified from Weishampel et al., 1993 character 20): (0) up to 0.35; (1) greater than 0.35 and up to 0.45; (2) greater than 0.45.

111. Ratio between the length of the ectopterygoid shelf and the anteroposterior length of the maxillary dental battery (corresponding with Prieto-Márquez and Wagner, 2009 character 105): (0) up to 0.25; (1) greater than 0.25 and at most 0.35; (2) more than 0.35.

112. Degree of the inclination of the ectopterygoid shelf (angle between the lateral margin of the ectopterygoid shelf and the anteroposterior axis of the maxillary dental battery in lateral view) (modified from Prieto-Márquez and Wagner, 2009 character 106): (0) presence of a steeply inclined posteroventrally ectopterygoid shelf with an angle greater than 20º; (1) presence of a markedly inclined posteroventrally ectopterygoid shelf with an angle from 10º to 20º; (2) presence of a slightly inclined posteroventrally ectopterygoid shelf with an angle greater than 5º and less than 10º; (3) presence of a almost horizontal ectopterygoid shelf with an angle up to 5º.

113. Development of the ectopterygoid ridge (modified from Godefroit et al., 2000 character 14): (0) presence of a less developed, dorsoventrally thin ridge on the lateral surface of the maxillary posterior portion; (1) presence of a faint ridge which is dorsoventrally thin anteriorly and gradually thickened posteriorly along the lateral margin of the maxillary posterior portion; (2) presence of a prominent, dorsoventrally thick ridge on the lateral surface of the maxillary posterior portion.

114. Location of the middle part of the special foramina (neurovascular alveolar foramina) on the medial side of the maxilla relative to half the dorsoventral height of the maxilla excluding the height of the dorsal process (corresponding with Prieto-Márquez and Wagner, 2009 character 108): (0) ventral to or at half the height of the maxilla; (1) dorsal to half the height of the maxilla.

Jugal

115. Morphology of the posterodorsal margin of the jugal anterior process (modified from Weishampel et al., 1993 character 15): (0) dorsoventrally narrow and slightly curved dorsally; (1) dorsoventrally deep and markedly dorsally recurved.

116. Anterior apex of the anterior process of the jugal (modified from Prieto-Márquez and

Wagner, 2009 character 111): (0) presence of a very long, wedge-shaped and sharp anterior apex located at the middle point of dorsoventral depth of the anterior process; (1) presence of a long, wedge-shaped and sharp anterior apex located near the dorsal half of the anterior process; (2) presence of a short, mammilla-shaped and blunt anterior apex near the dorsal half of the anterior process; (3) presence an extremely short, less developed and blunt anterior apex near the dorsal half of the anterior process; (4) absence of the anterior apex.

117. General shape of the ventral margin of the jugal anterior process (corresponding with Prieto-Márquez and Wagner, 2009 character 113): (0) bowed or curved; (1) inverted triangular, with the width approximately equal to the height; (2) inverted triangular and often recurved ventrally, with the height greater than the width.

118. Position of palatine articulating facet on the medial surface of the jugal anterior process (corresponding with Xing et al., 2012 character 113): (0) dorsal to the maxillary contact surface; (1) posterior to the maxillary contact surface and dorsal to the maxillary process of the jugal.

119. Degree of the inclination of the palatine contact surface of the jugal (angle between the long axis of the jugal and the long axis of palatine contact surface) (modified from Prieto-Márquez and Wagner, 2009 character 116): (0) extremely inclined anteriorly and almost horizontal, with an angle of approximately 180º; (1) strongly inclined anteriorly, with an angle between 110º and 150º; (2) slightly inclined anteriorly and almost vertical, with an angle less than 110º.

120. Location of the ventral apex of the jugal anterior process relative to the posterior margin of the lacrimal process of the jugal (the longitudinal axis of the anterior process represents horizontal) (corresponding with Prieto-Márquez and Wagner, 2009 character 114): (0) located posteroventral to the posterior margin of the lacrimal process; (1) located ventral to the posterior margin of the lacrimal process.

121. Morphology of the concave ventral margin of the jugal (modified from Norman, 2002 character 16): (0) relatively shallow and wide; (1) relatively deep and narrow.

122. Ratio between the maximum dorsoventral depth of the jugal posteroventral portion ventral to the infratemporal fenestra and minimum vertical distance of the posterior contractive portion of the jugal (modified from Prieto-Márquez and Wagner, 2009 character 118): (0) up to 1.35; (1) greater than 1.35 and up to 1.55; (2) more than 1.55.

123. General profile of the posterior process of the jugal in lateral view (corresponding with

Prieto-Márquez and Wagner, 2009 character 119): (0) auricular in shape and relatively slender, with a nearly straight posterior margin; (1) auricular in shape and relatively wide anteroposteriorly, with a convex posterior margin and a short blunt apex along the dorsal margin of the posterior process; (2) auricular in shape and relatively wide anteroposteriorly, with a convex posterior margin and a tall pointed apex along the dorsal margin of the posterior process; (3) regularly fan-shaped, with a nearly straight or slightly convex posterior margin.

124. Morphological character of the concavity of the posteroventral margin of the jugal (modified from Weishampel et al., 1993 character 18): (0) relatively short and shallow; (1) relatively long and deep.

125. Ratio between the minimum vertical distance of the posterior contractive portion of the jugal ventral to the infratemporal fenestra and the distance from the lowest point of the orbital rim to the lowest point of the rim of the infratemporal fenestra (modified from Weishampel et al., 1993 character 13): (0) up to 0.5; (1) greater than 0.5 and less than 0.6; (2) 0.6 to 0.8; (3) greater than 0.8.

126. Width of the orbital margin of the jugal relative to that of the infratemporal margin of the bone (corresponding with Prieto-Márquez and Wagner, 2009 character 123): (0) wider orbital margin; (1) the orbital margin and infratemporal margin are almost equally wide; (2) wider infratemporal margin.

127. Ectopterygoid contact facet on the medial side of the jugal (corresponding with Godefroit et al., 2001 character 12): (0) present; (1) absent.

Quadratojugal

128. General shape of the quadratojugal in lateral view (corresponding with Xing et al., 2012 character 123): (0) L-shaped; (1) trapeziform.

Quadrate

129. Degree of the curvature of the quadrate in lateral view (angle between the long axis of the dorsal portion of the quadrate and the one of the ventral portion of the quadrate) (modified from Wagner, 2001 character 35): (0) slightly curved posteriorly, with an angle more than 150°; (1) strongly curved posteriorly, with an angle up to 150°.

130. Position of the quadratojugal notch relative to the dorsoventral height of the quadrate (corresponding with Prieto-Márquez and Wagner, 2009 character 125): (0) the midpoint of the

quadratojugal notch is located near half the dorsoventral height of the quadrate; (1) the midpoint of the quadratojugal notch is located well below half the dorsoventral height of the quadrate.

131. Angle between the dorsal margin of the quadratojugal notch and the long axis of the quadrate (modified from Prieto-Márquez and Wagner, 2009 character 126): (0) greater than 40º; (1) up to 40º.

132. General shape of the quadratojugal notch of the quadrate (modified from Weishampel and Horner, 1986): (0) semicircular and relatively deep anteroposteriorly, with the ventral margin of the notch which is slightly recurved and directed anteriorly; (1) wide arcuate and relatively shallow anteroposteriorly, with the ventral margin of the notch which is smooth and directed anteroventrally; (2) wide arcuate and relatively shallow anteroposteriorly, with the ventral margin of the notch which is slightly recurved and directed anteriorly.

133. Squamosal buttress (posterodorsal protuberance) on the posterior side of the dorsal end of the quadrate (modified from Weishampel and Horner, 1986): (0) presence of a well developed protuberant buttress; (1) absence of the buttress or presence of a less developed faint buttress.

134. Ratio between the anteroposterior length of the lateral condyle and the mediolateral width of the ventral end of the quadrate (modified from Weishampel et al., 1993 character 22): (0) up to 0.75; (1) more than 0.75.

135. Elevation of the medial condyle of the quadrate in anterior view (modified from Prieto-Márquez and Wagner, 2009 character 129): (0) the medial condyle is slightly elevated upwards relative to the lateral condyle; (1) the medial condyle is remarkably elevated upwards relative to the lateral condyle.

136. Lateral exposure of the quadratojugal notch of the quadrate (modified from Prieto-Márquez and Wagner, 2009 character 198): (0) present; (1) absent.

Neurocranial Bones

Nasal

137. Hollow supracranial crest dorsal to the orbit (corresponding with Godefroit et al., 2008 character 6): (0) absent; (1) present.

138. Solid supracranial crest dorsal to the orbit (modified from Horner et al., 2004 character 38): (0) absent; (1) present but not laterally excavated by the posterior expansion of the circumnarial fossa; (2) present but laterally excavated by the posterior expansion of the circumnarial fossa.

139. Supracranial ornament dorsal to the orbit (modified from Horner et al., 2004 character 40): (0) absent; (1) present, composed of nasals; (2) present, composed of frontals and nasals; (3) present, composed of nasals and premaxillae.

140. Participation of the nasal in the hollow crest (modified from Godefroit et al., 2008 character 19): (0) absent, with the presence of the nasal anterior to the orbit forming a part of the margin of the external naris; (1) present, with the presence of the nasal anterior to the orbit forming a part of the margin of the external naris and bearing a less developed crest-like structure which is hollow; (2) present, with the presence of the well developed nasal dorsal to the orbit forming the posterior half of the hollow crest; (3) present, with the presence of the well developed nasal dorsal to the orbit forming the posteroventral portion of the hollow crest.

141. Position of the nasal cavity (common median chamber inside the crest) in the adult skull (modified from Horner et al., 2004 character 33): (0) restricted to a horizontal area anteromedial to the orbit; (1) restricted to an oval area anterodorsal and medial to the orbit; (2) restricted to an ellipsoid-shaped area dorsomedial to the orbit.

142. Shape of the posterior part of the nasals in non-lambeosaurine iguanodontians (corresponding with Xing et al., 2012 character 137): (0) presence of a relatively flat posterior part without any protuberance or crest; (1) presence of a low, dome-shaped protuberance on the dorsal surface of the posterior part; (2) presence of a transversely narrow, arched promontory in the posterior region; (3) presence of a dorsoventrally compressed, paddle-like posterior portion overhanging the braincase; (4) presence of an elongate, stick-shaped posterior portion, which bears a median dorsal ridge and is triangular in cross section, extending to the region dorsal to the occiput; (5) presence of a strongly anterodorsally curved and abruptly posterodorsally elevated posterior portion that forms a sharp mediolateral protuberance with the frontal; (6) presence of a gradually posterodorsally elevated, laterodorsally depressed and transversely broad nasal crest dorsal to the anterior part of the frontal.

143. Nasal inserting under the premaxillary posteroventral process (corresponding with Xing et al., 2012 character 138): (0) absent; (1) present.

144. Morphology of the anterior end of the nasal contacting the premaxillary posterodorsal process in lateral view (modified from Horner et al., 2004 character 34): (0) presence of a slender, wedge-shaped anterior end, gradually decreasing anteriorly in width and depth; (1) presence of a

relatively thick subrectangular anterior end, abruptly and markedly decreasing anteriorly in depth near the anteriormost end; (2) presence of a long, finger-shaped anterior end without any prominent change in depth; (3) presence of a vertical, rod-like nasal with a trough-shaped, dorsoventrally oriented anterior side meeting the premaxillary posterodorsal process; (4) presence of a relatively slender anterior end fitting along the ventral margin of premaxillary posterodorsal process; (5) presence of a thick, sheet-shaped anterior end bearing the bilobate anterior margin and meeting the premaxilla in a complex W-shaped interfingering suture.

145. Morphology of the posteroventral portion of the nasal that contacts the posteroventral process of the premaxilla in non-lambeosaurine iguanodontians (corresponding with Prieto-Márquez and Wagner, 2009 character 86): (0) the posterior region of the nasal forms a subrectangular flange, which is laterally exposed dorsal to the premaxillary posteroventral process; (1) the posterior region of the nasal forms a large, elongate, hook-like anteroventral process, which is laterally exposed dorsal to the premaxillary posteroventral process; (2) the posterior region of the nasal forms a greatly shortened, dorsoventrally narrow, hook-like anteroventral process, which is laterally exposed dorsal to the premaxillary posteroventral process.

146. Posteriormost apex of the external naris (corresponding with Godefroit et al., 2008 character 16): (0) consisting of the nasal and premaxilla; (1) consisting entirely of the nasal; (2) consisting entirely of the premaxilla.

147. Participation of the anterior end of the nasal in the anterior margin of the external naris (modified from Prieto-Márquez and Wagner, 2009 character 87): (0) absent, the anterior end of the nasal is located at the anterior one-third of the external naris; (1) absent, the anterior end of the nasal participating in the hollow supracranial crest is far away from the anterior margin of the external naris; (2) present, the anterior end of the nasal reaches the anterior margin of the external naris.

148. Posterior ends of the nasals forming a pair of processes along the frontonasal suture of the skull (modified from Gates and Sampson, 2007 character 65): (0) absent; (1) present, the posterior ends of the nasals have a pair of divergent processes lying on top of the frontals; (2) present, the posterior ends of the nasals have a pair of conjunctive processes inserted into the frontals.

149. A pronounced arched promontory in the posterodorsal region of the nasal, which is located anterodorsal to the orbit (modified from Prieto-Márquez and Wagner, 2009 character 91): (0)

absent; (1) present, the summit of the promontory is located dorsal to the posterior margin of the external naris; (2) present, the summit of the promontory is located posterodorsal to the posterior margin of the external naris.

150. Lateral profile of the dorsal margin of the rostrum dorsal to the external naris (modified from Prieto-Márquez and Wagner, 2009 character 176): (0) slightly convex and arcuate; (1) highly convex and arcuate; (2) basically straight.

151. Angle between the long axis of the external naris and the maxillary dental battery (modified from Prieto-Márquez and Wagner, 2009 character 177): (0) less than 30º; (1) 30º to 40º; (2) more than 40º.

152. Lateral exposure of the circumnarial fossa (nasal vestibule) (corresponding with Xing et al., 2012 character 147): (0) present, with the presence of the circumnarial fossa surrounding the external naris; (1) absent, with the presence of the circumnarial fossa enclosed within the posterodorsal and posteroventral premaxillary processes and internalized.

153. Roof of the nasal passage in the anterior region of the skull (modified from Horner et al., 2004 character 27): (0) formed by the nasal; (1) formed by the premaxillary posterodorsal process owing to the nasal passage completely enclosed by the tubular premaxilla.

154. Nasal passage divided into two parts (corresponding with Xing et al., 2012 character 149): (0) absent, only one nasal passage connecting with the cavum nasi proprium; (1) present, two nasal passages (left and right ones) connecting with the lateral diverticulum and common medial chamber.

155. Posterior extension of the nasal passage (modified from Evans and Reisz, 2007 character 9): (0) absent, the nasal passage restricted to the region anterior to the orbit; (1) present, the nasal passage extending to the region dorsal to the orbit; (2) present, the nasal passage extremely extending to the region posterodorsal to the occipital.

156. Narial foramen that is the external opening of the nasal (corresponding with Xing et al., 2012 character 151): (0) presence of the narial foramen which is equivalent to the external naris when the nasal is located anterior to the orbit; (1) absence of the narial foramen when the nasal extends above the orbit and becomes part of hollow supracranial crest.

157. General shape of the narial foramen in lateral view (corresponding with Prieto-Márquez and Wagner, 2009 character 179): (0) broad dorsoventrally and elliptic; (1) relatively narrow

dorsoventrally and subelliptic; (2) extremely narrow dorsoventrally and slit-shaped.

158. Premaxilla-nasal fontanelle surrounded by the nasal and the premaxilla on the lateral surface of the supracranial crest (modified from Norman, 2002 character 5): (0) absent; (1) present, the nasal passage is incompletely closed on the lateral surface of the crest because of the premaxilla-nasal fontanelle persisting into the late ontogenetic stage; (2) present, the nasal passage is completely closed on the lateral surface of the crest because of the premaxilla-nasal fontanelle disappearing in the adult stage.

159. Ratio between the length of the external naris and the length of the lateroventral margin of the rostrum along the circumnarial depression in non-lambeosaurine iguanodontians (corresponding with Prieto-Márquez and Wagner, 2009 character 181): (0) up to 0.40; (1) greater than 0.40 but less than 0.60; (2) 0.60 to 0.65; (3) greater than 0.65.

160. Posterior margin of the circumnarial fossa posterodorsal to the narial foramen (corresponding with Horner et al., 2004 character 31): (0) absent, the circumnarial fossa does not reach the posterior margin of the narial foramen; (1) present, the circumnarial fossa reaches the posterior margin of the narial foramen.

161. Morphological character of the posterior region of the circumnarial fossa along the posterior margin of the external naris (corresponding with Horner et al., 2004 character 32): (0) absent; (1) present, slightly incised into nasal and premaxilla, often poorly demarcated; (2) present, deeply incised into nasal and premaxilla, well demarcated, and usually invaginated.

162. Strongly invaginated, excavated circumnarial fossa enclosing the entire external naris (corresponding with Xing et al., 2014 character 163): (0) absent; (1) present, the circumnarial fossa is dorsoventrally tall along its entire length, with a prominent, arched posterodorsal margin; (2) present, the circumnarial fossa is unusually low dorsoventrally in its middle and posterior regions, with a thick, right-angled posterodorsal margin.

163. Strong elevation or specialized development of the nasal (corresponding with Prieto-Márquez and Wagner, 2009 character 190): (0) absent; (1) present.

164. Morphology of the hollow supracranial crest in lambeosaurine dinosaurs (modified from Horner et al., 2004 character 36): (0) the nasal anterior to the orbit forms the posterodorsal margin of the external naris, bearing a less developed crest-like structure which is hollow and protuberant; (1) the nasal, which extends dorsally to follow the posterodorsal expansion of the premaxilla,

forming a vertical tube-shaped structure dorsal to the orbit; (2) the premaxilla extends posterodorsally to facilitate the backward growth and development of the nasal, forming a long curved tubular hollow crest dorsal to the occiput with the nasal; (3) the premaxilla extends posterodorsally to facilitate the backward growth and development of the nasal, forming a cockscomb-shaped hollow crest dorsal to the occiput with the nasal.

165. Position of the posteriormost end of the nasal of adult lambeosaurine dinosaurs in lateral view (corresponding with Prieto-Marquez and Wagner, 2009 character 195): (0) anterodorsal to the orbit; (1) dorsal to the posterior margin of the orbit; (2) dorsal to the occiput.

166. S-loop in the nasal passage (nasal vestibule) completely enclosed by premaxilla anterior to the orbit (corresponding with Evans and Reisz, 2007 character 8): (0) absent; (1) present.

167. Lateral diverticulum connecting with common medial chamber of nasal cavity (corresponding with Xing et al., 2012 character 161): (0) absence of the lateral diverticulum; (1) presence of the lateral diverticulum as a part of the cavum nasi proprium (the lateral diverticulum and common median chamber are together homologous with the cavum nasi proprium).

168. Position of the lateral diverticulum relative to the common median chamber (corresponding with Weishampel, 1981): (0) located posterodorsal to the common median chamber; (1) located lateral to the common median chamber; (2) located anterior to the common median chamber.

169. Connection between the nasal passage and the lateral diverticulum inside the hollow crest (corresponding with Weishampel, 1981 and Evans, 2006): (0) absent, the nasal passage completely enclosed by the premaxilla connects directly with the common median chamber without the participation of the lateral diverticulum; (1) present, the nasal passage connects with the lateral diverticulum which communicates with the common median chamber in succession.

Lacrimal

170. Lateral profile of the lacrimal in articulated adult cranium (modified from Prieto-Márquez and Wagner, 2009 character 109): (0) subquadrangular or trapeziform, with a relatively short anteroventral corner; (1) trapeziform and anteroposteriorly elongate, bearing a dorsoventrally low, relatively long anterior process; (2) triangular and anteroposteriorly elongate, with a straight, dorsoventrally thick anterior process; (3) triangular and anteroposteriorly elongate, with a curved, slender anterior process.

Prefrontal

171. Participation of the prefrontal in the lateral border of the hollow supracranial crest (corresponding with Godefroit et al., 2008 character 22): (0) absent; (1) present.

172. A well-developed, anteroposteriorly oriented ridge on the dorsal surface of the prefrontal as the medial wall of the anterior platform supporting the base of the hollow crest (modified from Prieto-Márquez and Wagner, 2009 character 130): (0) absence of the prefrontal ridge; (1) presence of the prefrontal ridge that does not extend posteriorly to the prefrontofrontal suture; (2) presence of the prefrontal ridge that extends posteriorly over the dorsal surface of the frontal.

173. General shape of the anterodorsal margin of the prefrontal along the orbital rim in lateral view (modified from Horner et al., 2004 character 50): (0) arcuate or smoothly curved; (1) nearly right-angled.

174. Morphology of the laterally exposed prefrontal anterior portion contacting with the lacrimal (corresponding with Prieto-Márquez and Wagner, 2009 character 132): (0) mediolaterally wide and relatively robust in lateral view; (1) mediolaterally narrow and relatively slender in lateral view.

175. Eversion of the anterodorsal orbital margin of the prefrontal (corresponding with Horner et al., 2004 character 49): (0) absent, the prefrontal lies flush with the elements nearby; (1) present, the prefrontal flares dorsolaterally to form a thin, everted, wing-shaped margin around the anterodorsal orbital rim.

176. Degree of the lateral exposure of the prefrontal-nasal contact (modified from Wagner, 2001 character 37): (0) the prefrontal-nasal contact is completely exposed in lateral view; (1) only the posterior region of the prefrontal-nasal contact is visible in lateral view, owing to the covering of the premaxilla along the laterodorsal margin of the prefrontal.

Palpebral

177. Palpebral (supraorbital) bone (corresponding with Norman, 2002 character 13): (0) present; (1) absent or fused to the orbital margin.

Postorbital

178. Dorsal promontorium on the dorsal surface of the postorbital anterior process in adults (corresponding with Godefroit et al., 2004 character 17): (0) absent; (1) present.

179. A prominent depression on the dorsal surface of the postorbital above the ventral process of the element (corresponding with Xing et al., 2012 character 173): (0) absent, the dorsal surface of

the postorbital is flat or slightly concave; (1) present, the dorsal surface of the postorbital is deeply depressed.

180. Degree of the curvature of the posterodorsal orbital margin of the postorbital (modified from Xing et al., 2012 character 174): (0) presence of a slightly concave anteroventrally or nearly straight orbital margin obliquely bridging the anterior and ventral processes of the postorbital; (1) presence of a strongly concave anteroventrally orbital margin that is often semicircular or deeply arcuate.

181. Morphology of the triangular ventral process of the postorbital (modified from Prieto-Márquez and Wagner, 2009 character 138): (0) presence of a relatively robust ventral process in lateral view, strongly expanding anteroventrally along the anterior margin of its dorsal half; (1) presence of a slender ventral process that is gradually tapering ventrally; (2) presence of an anteroposteriorly broad ventral process in lateral profile, strongly expanded along its anterior and posterior margins.

182. Strongly bulgy laterally main body of the postorbital bearing a deep inner cavity located anterior to the medial ridge of the ventral process of the postorbital (modified from Prieto-Márquez and Wagner, 2009 character 138): (0) absent; (1) present.

183. Morphological character of the posterior process (squamosal process) of the postorbital (corresponding with Evans and Reisz, 2007 character 36): (0) presence of a relatively long posterior process forming a broad dorsal margin of the infratemporal fenestra; (1) presence of a relatively short and dorsoventrally deep posterior process that forms a truncated dorsal margin of the infratemporal fenestra.

184. Bifurcation of the posterior process of the postorbital (corresponding with Evans and Reisz, 2007 character 35): (0) absent; (1) present.

185. Length of the ventral process of the postorbital relative to maximum anteroposterior width of the orbit (modified from Prieto-Márquez and Wagner, 2009 character 139): (0) presence of a relatively short ventral process which is shorter than the anteroposterior width of the orbit; (1) presence of a relatively long vental process which is approximately equal to the anteroposterior width of the orbit.

186. Position of the posterior end of the postorbital posterior process relative to the quadrate cotylus of the squamosal (corresponding with Prieto-Márquez and Wagner, 2009 character 141):

(0) located anterior to the quadrate cotylus; (1) located above the anterior half of the quadrate cotylus; (2) located dorsal to the posterior end of the quadrate cotylus.

Squamosal

187. Dorsoventral expansion of the main body of the squamosal in lateral view (modified from Horner et al., 2004 character 64): (0) slightly expanded dorsoventrally above the quadrate cotylus; (1) markedly expanded dorsoventrally and elevated above the quadrate cotylus.

188. Degree of the elongation of the precotyloid process of the squamosal (ratio between the length of the precotyloid process and the width of the quadrate cotylus of the squamosal) (corresponding with Prieto-Márquez and Wagner, 2009 character 142): (0) presence of a relatively short precotyloid process, with the ratio less than 1.00; (1) presence of a moderately elongate precotyloid process, with the ratio from 1.00 to 1.25; (2) presence of a very long precotyloid process, with the ratio greater than 1.25.

189. Strong anteromedial curvature of the medial ramus of the squamosal (corresponding with Prieto-Márquez and Wagner, 2009 character 145): (0) present; (1) absent.

190. Complete separation of the squamosals along the occipital margin of the skull roof in adults (corresponding with Horner et al., 2004 character 63): (0) present, the paired squamosals are widely separated by the parietal; (1) present, the squamosals approach the midline of the skull, and are separated by a narrow band of the parietal; (2) absent, the medial rami of the squamosals contact with each other.

Frontal

191. Anteroventrally sloping frontal platform (nasal sutural surface of the frontal) for supporting the supracranial crest (corresponding with Xing et al., 2012 character 184): (0) absent; (1) present.

192. Deeply excavated anterior platform composed of the prefrontal and the frontal (corresponding with Godefroit et al., 2008 character 8): (0) absent; (1) present, the anterior platform is inclined anteroventrally and occupies the anterior part of the frontal in adults; (2) present, the anterior platform markedly extends posterodosally and overhangs the parietal in adults.

193. Ratio between the anteroposterior length of the nasal contact surface of the frontal and the total length of the frontal (corresponding with Wang et al., 2015 character 193): (0) less than 0.40; (1) 0.40 or greater.

194. Angle between the dorsal surface of the frontal anterior part and the level (corresponding with Xing et al., 2012 character 186): (0) up to 10º; (1) greater than 10º and less than 25º; (2) 25º to 35º; (3) more than 35º.

195. Median cleft on the nasal sutural surface of the frontal (corresponding with Evans and Reisz, 2007 character 40): (0) absent; (1) present.

196. Frontal doming on the dorsal surface of the braincase in subadult or adult individuals (modified from Horner et al., 2004 character 58): (0) absent; (1) present.

197. Participation of the frontal in the dorsal margin of the orbit (corresponding with Horner et al., 2004 character 57): (0) present, the frontal forms part of the orbital margin; (1) absent, the frontal is excluded by prefrontal-postorbital contact.

198. Ratio between the anteroposterior length of the interfrontal suture of the frontal ectocranial surface and the maximum mediolateral width of the frontal ectocranial surface in dorsal view (modified from Godefroit et al., 2008 character 7): (0) presence of a relatively long ectocranial surface, with the ratio greater than 0.8; (1) presence of a relatively short ectocranial surface because of the extension of the nasal articular surface of the frontal, with the ratio from 0.4 to 0.8; (2) presence of an extremely short ectocranial surface by reason of the thickening of the nasal sutural surface of the frontal which obviously extends posterodosally and overhangs the parietal, with the ratio less than 0.4.

199. Fontanelle of the skull roof in juvenile or subadult individuals (corresponding with Xing et al., 2012 character 191): (0) presence of the nasal-frontal fontanelle surrounded by the nasal and the frontal; (1) presence of the premaxilla-nasal fontanelle enclosed by the nasal and the premaxilla; (2) absence of the fontanelle of the skull roof.

200. Nasal-frontal fontanelle on the dorsal surface of the braincase (modified from Prieto-Márquez and Wagner, 2009 character 147): (0) present, the nasal-frontal fontanelle is still well developed and large in subadults or young adults; (1) absent, the nasal-frontal fontanelle is strongly contractive and entirely closed in subadults or young adults; (2) absent, the nasal-frontal fontanelle cannot be identified owing to the development of the supracranial crest.

201. Morphological character of the annular ridge on the ventral side of the frontal that defines the anterior extent of the cerebral fossa (corresponding with Evans and Reisz, 2007 character 43): (0) relatively wide and shallow; (1) relatively narrow and sharp.

Parietal

202. Ratio between the anteroposterior length of the parietal along the sagittal plane and the width of the parietal at half the length in adults (modified from Godefroit et al., 2008 character 2): (0) less than 2.2; (1) 2.2 or greater.

203. Lateral profile of the parietal sagittal crest (modified from Horner et al., 2004 character 69): (0) the sagittal crest is nearly straight or slightly down-warped, level with the skull roof; (1) this crest is nearly straight, gradually ascending posteriorly relative to the skull roof; (2) this crest is strongly downwarped well below the skull roof.

204. Morphology of the median anterior process of the parietal along the frontoparietal suture (modified from Prieto-Márquez and Wagner, 2009 character 157): (0) arcuate or subtriangular, anteroposteriorly short and mediolaterally wide; (1) finger-shaped or subtriangular, anteroposteriorly long and mediolaterally narrow.

205. Morphology of the sagittal crest on the dorsal surface of the parietal (modified from Horner et al., 2004 character 70): (0) the sagittal crest only extends along the anterior half of the parietal, and splits into two secondary ridges on the dorsal surfaces of paired posterolateral processes; (1) the sagittal crest extends along the entire length of the parietal, and its anterior half is relatively wide mediolaterally, gradually narrowing and sharpening posteriorly; (2) the sagittal crest extends along the entire length of the parietal, and keeps narrow and sharp all the while.

206. Angle between the paired posterolateral secondary ridges of the parietal sagittal crest (modified from Xing et al., 2014 character 206): (0) up to 60º, including the absence of secondary ridges; (1) more than 60º, usually with an obvious longitudinal eminence in between.

Supraoccipital

207. Orientation of the posterior surface of the supraoccipital (corresponding with Horner et al., 2004 character 65): (0) nearly vertical and directed posteriorly; (1) markedly inclined anteriorly and directed posterodorsally.

208. Lateroventral portion of the supraoccipital deeply inserted into the exoccipital, with two flanges along the supraoccipital-exoccipital suture in posterior view (corresponding with Horner et al., 2004 character 66): (0) absent; (1) present.

Exoccipital

209. Degree of the posterodorsal expansion of the exoccipital central shelf relative to the

foramen magnum in ventral view (modified from Godefroit et al., 2008 character 26): (0) slightly elongate posterodorsally; (1) moderately expanded posterodorsally; (2) extremely expanded posterodorsally.

210. Orientation of the distal end of the paroccipital process (corresponding with Horner et al., 2004 character 62): (0) anteroventrally directed; (1) ventrally directed.

Basioccipital

211. Participation of the basioccipital in the ventral margin of the foramen magnum (corresponding with Weishampel et al., 1993 character 24): (0) absent, the paired exoccipital condyles completely exclude the basioccipital from the ventral margin of the foramen magnum; (1) present, the exoccipital condyles are well separated and allow the basioccipital to form the ventral margin of the foramen magnum.

212. Shallow groove of the occipital condyle along the exoccipital-basioccipital junction in posterior view (modified from Prieto-Márquez and Wagner, 2009 character 160): (0) absent; (1) present.

213. Degree of the constriction of the basioccipital portion between the occipital condyle and the spheno-occipital tubercles (corresponding with Prieto-Márquez and Wagner, 2009 character 161): (0) strongly constricted, forming a relatively long constricted portion of the basioccipital; (1) faintly constricted, forming a relatively short constricted portion of the basioccipital.

Basisphenoid

214. Length of the basipterygoid process of the basisphenoid (corresponding with Godefroit et al., 2001 character 2): (0) very short; (1) relatively long, markedly extending below the ventral border of the occipital condyle.

215. Angle between the ventral margin of the paired basipterygoid processes of the basisphenoid (modified from Prieto-Márquez and Wagner, 2009 character 162): (0) greater than 100º; (1) up to 100º.

216. Development of the paired alar processes of the basisphenoid (corresponding with Prieto-Márquez and Wagner, 2009 character 163): (0) moderately developed and relatively small in size; (1) well developed and relatively large in size.

217. Ventral transverse ridge between the basipterygoid processes of the basisphenoid (corresponding with Gates and Sampson, 2007 character 78): (0) present; (1) absent.

218. Ventral median process between the basipterygoid processes of the basisphenoid (corresponding with Gates and Sampson, 2007 character 79): (0) present; (1) absent.

219. Ratio between the maximum width across the spheno-occipital tubercles and the minimum width of the anterior constriction of the basisphenoid (modified from Prieto-Márquez and Wagner, 2009 character 166): (0) less than 1.5; (1) 1.5 to 1.9; (2) greater than 1.9.

Laterosphenoid

220. Complete closure of the forward sulcus as the passage of the ramus ophthalmicus of the trigeminal nerve (V_1) on the lateral surface of the laterosphenoid (corresponding with Evans and Reisz, 2007 character 51): (0) absent; (1) present.

221. Great reduction of the length of the postorbital process of the laterosphenoid (corresponding with Prieto-Márquez and Wagner, 2009 character 168): (0) absent; (1) present.

Pterygoid

222. Dorsal expansion of the posterodorsal border of the pterygoid palatine ramus forming a small flange (corresponding with Prieto-Márquez and Wagner, 2009 character 173): (0) absent; (1) present.

223. Ventral expansion of the lamina restricted by two ventrally oriented ridges along the medial surface of the ectopterygoid process and the quadrate ramus of the pterygoid (corresponding with Prieto-Márquez and Wagner, 2009 character 174): (0) moderately expanded ventrally, relatively large portions of the ectopterygoid process and the quadrate ramus are located below the ventral margin of the lamina; (1) markedly expanded ventrally, only relatively small portions of the ectopterygoid process and the quadrate ramus are located below the ventral margin of the lamina.

Regional Cranial Characters

224. General shape of the orbit in lateral view (modified from Prieto-Márquez and Wagner, 2009 character 197): (0) almost circular; (1) elliptic, the anteroposterior width of the orbit is less than the dorsoventral height of this element.

225. Anteroposterior width of the orbit relative to that of the infratemporal fenestra (modified from Prieto-Márquez and Wagner, 2009 character 199): (0) the anteroposterior width of the orbit is approximately equal to that of the infratemporal fenestra; (1) the anteroposterior width of the orbit is greater than that of the infratemporal fenestra.

226. Position of the dorsal margin of the orbit relative to that of the infratemporal fenestra

(corresponding with Prieto-Márquez and Wagner, 2009 character 201): (0) the dorsal margin of the orbit and the dorsal margin of the infratemporal fenestra are generally at the same level and height; (1) the dorsal margin of the orbit is obviously lower than that of the infratemporal fenestra; (2) the dorsal margin of the orbit is slightly higher than that of the infratemporal fenestra.

227. General shape of the infratemporal fenestra in lateral view (corresponding with Xing et al., 2012 character 218): (0) subrectangular in shape; (1) subtriangular in shape.

228. Anteroposterior width of the dorsal margin relative to that of the ventral margin of the infratemporal fenestra (corresponding with Prieto-Márquez and Wagner, 2009 character 200): (0) the dorsal margin of the infratemporal fenestra is approximately as wide as the ventral margin of this element; (1) the dorsal margin of the infratemporal fenestra is narrower than the ventral margin of this element.

229. General shape of the supratemporal fenestra in dorsal view (modified from Prieto-Márquez and Wagner, 2009 character 202): (0) subrectangular, with the long axis directed anteriorly; (1) oval, with the long axis directed anterolaterally.

230. Length/height ratio of the skull (the length of the skull is measured from the posterior margin of the quadrate to the anteriormost end of the premaxilla, and the height of the skull is measured from the ventral margin of the quadrate to the dorsal margin of the squamosal) (modified from You et al., 2003a character 1): (0) up to 2; (1) greater than 2.

231. Ratio between the length of the external naris and that of the skull (the length of the skull is measured from the posterior margin of the quadrate to the anteriormost end of the premaxilla) (modified from Horner et al., 2004 character 28): (0) up to 0.25; (1) greater than 0.25 but less than 0.40; (2) 0.40 or greater.

232. Ratio between the mediolateral width of the skull roof across the postorbitals and that across the quadrate cotyli of the paired squamosals (modified from Prieto-Márquez and Wagner, 2009 character 204): (0) more than 1.20; (1) up to 1.20.

233. Shape of the occiput in posterior view (modified from Horner et al., 2004 character 68): (0) rectangular; (1) trapezoidal.

234. Angle of the ventral deflection of the occipital condyle relative to the horizontal line (corresponding with Xing et al., 2014 character 234): (0) up to 155º; (1) greater than 155º.

235. Basisphenoid participating in the anteroventral margin of the foramen for the trigeminal

nerve (corresponding with Xing et al., 2014 character 235): (0) present; (1) absent.

Trunk Skeleton

Cervical Vertebrae

236. Number of the cervical vertebrae (corresponding with Horner et al., 2004 character 72): (0) 11 or fewer; (1) 12 or more.

237. Morphology of the dorsal margin of the axis (corresponding with Prieto-Márquez and Wagner, 2009 character 206): (0) presence of a convex dorsal margin of the neural spine extending to the region above the postzygapophyses; (1) presence of a concave dorsal margin in the posterior region of the axial neural spine.

238. Ratio between the anteroposterior length of the postzygapophyseal process and the anteroposterior width of the neural arch of the anterior and middle cervical vertebrae (modified from Prieto-Márquez and Wagner, 2009 character 207): (0) less than 3; (1) 3 or greater.

239. Morphological character of the postzygapophyseal processes of the cervical vertebrae (corresponding with Horner et al., 2004 character 74): (0) low and relatively short; (1) elevated and relatively long.

Dorsal Vertebrae

240. Number of the dorsal vertebrae, not including the last dorsal vertebra (the dorsosacral vertebra) incorporated into the sacrum (corresponding with Xing et al., 2012 character 229): (0) 16 or fewer; (1) 17 or more.

241. Slightly elongated neural spines of the anterior dorsal vertebrae (corresponding with Prieto-Márquez and Wagner, 2009 character 210): (0) absent; (1) present.

242. Ratio between the height and the length of the neural spines of the middle dorsal vertebrae (modified from Norman, 2002 character 41): (0) greater than 2.5 but less than 4.0; (1) 4.0 or greater.

243. Neural spine height greater than 4 times centrum height in the middle dorsal vertebrae (corresponding with Evans and Reisz, 2007 character 67): (0) absent; (1) present.

Sacral Vertebrae

244. Number of the sacral vertebrae, including the dorsosacral and caudosacral vertebrae fused to the sacrum (corresponding with Godefroit et al., 2000 character 27): (0) 7 or fewer; (1) 8 or more.

245. Ratio between the height of the neural spine and that of the centrum of the tallest sacral vertebra (modified from Prieto-Márquez and Wagner, 2009 character 209): (0) up to 2.0; (1) greater than 2.0 and up to 3.5; (2) greater than 3.5.

246. Participation of the caudal vertebrae (the caudosacral vertebrae) in the sacrum (corresponding with Xing et al., 2012 character 234): (0) absent; (1) present.

Caudal Vertebrae

247. Length of the chevrons relative to that of the neural spines in the anterior region of the caudal vertebrae (corresponding with Prieto-Márquez and Wagner, 2009 character 212): (0) the chevrons are shorter than or nearly as long as the neural spines; (1) the chevrons are longer than the neural spines.

Sternum

248. Length of the handle-shaped process (posterolateral process) relative to that of the proximal plate (anteromedial plate) of the sternum (corresponding with Prieto-Márquez and Wagner, 2009 character 213): (0) the distal handle-shaped process is slightly shorter than or as long as the proximal plate; (1) the distal handle-shaped process is longer than the proximal plate.

Shoulder Girdle

Coracoid

249. Coracoid size relative to the length of the scapula (corresponding with Horner et al., 2004 character 77): (0) relatively large in size; (1) reduced in size relative to the scapula.

250. Morphological character of the anterodorsal margin of the coracoid (modified from Horner et al., 2004 character 78): (0) straight or convex in lateral view; (1) concave in lateral view.

251. Development of the biceps tubercle in the anterior region of the coracoid (corresponding with Godefroit et al., 2008 character 47): (0) presence of a relatively small and slightly projecting biceps tubercle; (1) presence of a relatively large and markedly projecting laterally biceps tubercle.

252. Ratio between the length of the lateral margin of the scapular articular surface and that of the lateral margin of the glenoid (modified from Prieto-Márquez and Wagner, 2009 character 215): (0) greater than 1.3; (1) 1.0 to 1.3; (2) less than 1.0.

253. Angle between the lateral margin of the scapular articular surface and the lateral margin of the glenoid of the coracoid (corresponding with Prieto-Márquez and Wagner, 2009 character 216):

(0) greater than 115º; (1) up to 115º.

254. Morphological character of the hook-shaped ventral process of the coracoid (corresponding with Horner et al., 2004 character 79): (0) relatively short and directed ventrally; (1) relatively long, recurved and directed posteroventrally.

255. Ratio between the dorsoventral height and the anteroposterior width of the hook-shaped ventral process of the coracoid (modified from Prieto-Márquez and Wagner, 2009 character 218): (0) less than 0.6; (1) 0.6 to 0.8; (2) more than 0.8.

Scapula

256. General profile of the dorsal margin of the scapula in lateral view (modified from Sereno, 1986): (0) nearly straight anteroposteriorly from the anterior end of the acromial process to the posterior margin of the scapular blade; (1) obviously curved from the anterior end of the acromial process to the posterior margin of the scapular blade.

257. General profile of the ventral margin along the posterior half of the scapular blade (corresponding with Prieto-Márquez and Wagner, 2009 character 222): (0) almost straight or slightly convex ventrally; (1) remarkably convex ventrally.

258. Orientation of the dorsal margin of the scapular distal end relative to the ventral margin of this structure (modified from Horner et al., 2004 character 81): (0) the dorsal margin of the scapular distal end is markedly sloping posterodorsally and divergent relative to the ventral margin of this structure; (1) the dorsal margin of the scapular distal end is slightly divergent relative to the ventral margin of this structure; (2) the dorsal margin of the scapular distal end is nearly parallel to the ventral margin of this structure, so that they are in the same direction.

259. Ratio between the maximum dorsoventral depth of the scapular proximal end and the distance from the anterior end of the acromial process to the posterior margin of the scapular blade (modified from Prieto-Márquez and Wagner, 2009 character 221): (0) greater than 0.25; (1) up to 0.25.

260. Ratio between the maximum dorsoventral depth of the scapular blade and that of the proximal end of the scapula (corresponding with Prieto-Márquez and Wagner, 2009 character 223): (0) less than 1; (1) 1 or greater.

261. Ratio between the dorsoventral depth of the scapular neck and the maximum dorsoventral depth of the scapular proximal end (corresponding with Prieto-Márquez and Wagner, 2009

character 224): (0) up to 0.6; (1) more than 0.6.

262. Ratio between the distance from the anterior margin of the coracoid facet to the anteriormost end of the acromial process and the maximum dorsoventral height of the scapular anterior end (corresponding with Prieto-Márquez and Wagner, 2009 character 227): (0) less than 0.45; (1) 0.45 or greater.

263. Morphological character of the acromial process of the scapula (modified from Horner et al., 2004 character 80): (0) strongly recurved, with the anterior end of the acromial process directed dorsally; (1) slightly recurved, with the anterior end of the acromial process directed anterodorsally; (2) almost straight, with the anterior end of the acromial process directed anteriorly.

264. Development of the deltoid ridge on the lateral surface of the scapular anterior portion (corresponding with Prieto-Márquez and Wagner, 2009 character 228): (0) presence of a poorly developed deltoid ridge which is dorsoventrally narrow and relatively faint; (1) presence of a well developed deltoid ridge which is dorsoventrally deep and relatively sharp.

Forelimb and Manus

Humerus

265. Morphology of the humerus in posterior view (modified from Weishampel et al., 1993 character 36): (0) relatively long and gracile; (1) relatively short and robust.

266. Development of the deltopectoral crest in adults (modified from Godefroit et al., 2000 character 26): (0) presence of a poorly developed deltopectoral crest moderately expanded anterolaterally and ventrally; (1) presence of a well developed deltopectoral crest markedly expanded anterolaterally and ventrally.

267. Position of the maximum lateral expansion of the deltopectoral crest relative to the midshaft of the humerus (modified from Horner et al., 2004 character 83): (0) located above the midshaft of the humerus; (1) located at or below the midshaft of the humerus.

268. Strong constriction of the distal half of the humerus below the deltopectoral crest (corresponding with Xing et al., 2012 character 256): (0) present; (1) absent.

269. Ratio between the distance from the dorsal margin of the lateral tuberosity to the maximum lateral expansion of the deltopectoral crest and the proximodistal length of the humerus (corresponding with Prieto-Márquez and Wagner, 2009 character 229): (0) less than 0.48; (1) 0.48

to 0.55; (2) greater than 0.55.

270. Ratio between the width of the humerus at the distal third of the deltopectoral crest and the width of the distal constriction of the humerus (corresponding with Prieto-Márquez and Wagner, 2009 character 230): (0) less than 1.65; (1) 1.65 to 1.90; (2) greater than 1.90.

271. Angle between the anterolateral margin and the ventral margin of the humeral deltopectoral crest (modified from Weishampel et al., 1993 character 37): (0) greater than 130º; (1) 110º to 130º; (2) less than 110º.

272. Ratio between the width of the humerus at the midshaft and the proximodistal length of the humerus in posterior view (corresponding with Xing et al., 2012 character 260): (0) up to 0.20; (1) greater than 0.20 but less than 0.25; (2) 0.25 or greater.

273. Torsion between the proximal and distal ends of the humerus (corresponding with Xing et al., 2012 character 261): (0) moderate, with the angle up to 20º; (1) very strong, with the angle more than 20º.

Ulna

274. Ratio between the proximodistal length of the ulna and the anteroposterior width of this element at the midshaft (corresponding with Prieto-Márquez and Wagner, 2009 character 233): (0) less than 10; (1) 10 or greater.

275. Ratio between the proximodistal length of the ulna and that of the humerus (modified from Norman, 2002 character 47): (0) presence of a relatively short ulna, with the ratio up to 1.0; (1) presence of a moderately long ulna, with the ratio greater than 1.0 and up to 1.2; (2) presence of a very long ulna, with the ratio greater than 1.2.

Carpals

276. Composition of the carpus (corresponding with Horner et al., 2004 character 86): (0) presence of fused ulnare, radiale, intermedium and distal carpals; (1) presence of two small unfused carpals.

Metacarpals

277. Metacarpal I (modified from Norman, 2002 character 49): (0) present and fused to the carpus; (1) absent.

278. Ratio between the proximodistal length and the mediolateral width at the midshaft of metacarpal III (modified from Horner et al., 2004 character 89): (0) up to 5; (1) greater than 5.

279. Ratio between the proximodistal length of metacarpal V and the mediolateral width of the proximal end of this bone (modified from Prieto-Márquez and Wagner, 2009 character 238): (0) less than 2; (1) 2 or greater.

280. Strong mediolateral expansion of the proximal end of metacarpal V relative to the distal end (corresponding with Prieto-Márquez and Wagner, 2009 character 239): (0) absent; (1) present.

281. Position of the proximal end of the metacarpal III (corresponding with Horner et al., 2004 character 88): (0) the proximal end of the metacarpal III is aligned with those of the metacarpal II and IV; (1) the proximal end of the metacarpal III is offset distally relative to those of the metacarpal II and IV.

Manual Phalanges

282. Morphological character of manual phalanx III-1 in dorsal view (modified from Prieto-Márquez and Wagner, 2009 character 240): (0) moderately elongated proximodistally, with the proximodistal length almost equal to or slightly greater than the mediolateral width; (1) greatly elongated proximodistally, with the proximodistal length obviously greater than the mediolateral width; (2) strongly compressed proximodistally, with the proximodistal length less than the mediolateral width.

283. Manual digit I (corresponding with Norman, 2002 character 51): (0) present; (1) absent.

284. General shape of manual ungual II (corresponding with Norman, 2002 character 53): (0) claw-shaped; (1) hoof-shaped.

285. General shape of manual phalanx III-2 in dorsal view (corresponding with Horner et al., 2004 character 90): (0) rectangular, the medial and lateral margins are subequal in length; (1) wedge-shaped and strongly compressed, the medial margin is significantly shorter than the lateral margin.

286. Proximodistal length of manual phalanx II-1 relative to that of the manual phalanx II-2 (modified from Prieto-Márquez and Wagner, 2009 character 242): (0) manual phalanx II-1 is less than three times as long as manual phalanx II-2; (1) manual phalanx II-1 is three times or more as long as manual phalanx II-2.

Pelvic Girdle

Ilium

287. Degree of the ventral deflection of the iliac preacetabular process (modified from Suzuki et

al., 2004 character 69): (0) slightly deflected ventrally, with the angle greater than 150º; (1) markedly deflected ventrally, with the angle up to 150º.

288. Ratio between the anteroposterior length of the preacetabular process and that of the central plate (main blade) of the ilium (modified from Prieto-Márquez and Wagner, 2009 character 244): (0) up to 1.7; (1) more than 1.7.

289. Ratio between the maximum dorsoventral depth of the posterior end of the preacetabular process and the dorsoventral distance from the pubic peduncle to the dorsal margin of the ilium (corresponding with Prieto-Márquez and Wagner, 2009 character 245): (0) less than 0.50; (1) 0.50 to 0.55; (2) greater than 0.55.

290. Ratio between the dorsoventral height and anteroposterior length of the iliac central plate (corresponding with Prieto-Márquez and Wagner, 2009 character 246): (0) 0.8 or greater; (1) less than 0.8.

291. Position of the apex of the supraacetabular process (antitrochanter) relative to the posterior end of the iliac ischial peduncle (corresponding with Brett-Surman and Wagner, 2007): (0) located posterodorsal to the posterior end of the iliac ischial peduncle; (1) located anterodorsal to the posterior end of the iliac ischial peduncle.

292. Ratio between the anteroposterior breadth of the supraacetabular process along its dorsal margin and the anteroposterior length of the iliac central plate (modified from Prieto-Márquez and Wagner, 2009 character 249): (0) greater than 0.85; (1) greater than 0.70 and up to 0.85; (2) 0.55 to 0.70; (3) less than 0.55.

293. Lateroventral expansion of the iliac supraacetabular process (modified from Horner et al., 2004 character 91): (0) absent, the supraacetabular process slightly swells laterally along the dorsal margin of the central plate of the ilium; (1) present, the supraacetabular process slightly expands lateroventrally, with its lateroventral margin located at three-fourths of the dorsoventral height of the iliac central plate; (2) present, the supraacetabular process moderately expands lateroventrally, with its lateroventral margin located at approximately half dorsoventral height of the iliac central plate; (3) present, the supraacetabular process extremely expands lateroventrally and nearly overlaps the whole central plate of the ilium in lateral view.

294. General shape of the lateroventral margin of the iliac supraacetabular process in lateral view (corresponding with Prieto-Márquez and Wagner, 2009 character 251): (0) strip-shaped; (1)

widely arcuate; (2) U-shaped or V-shaped.

295. Symmetry of the lateral profile of the supraacetabular process (corresponding with Prieto-Márquez and Wagner, 2009 character 250): (0) absent; (1) present.

296. A strong ridge connecting the posteroventral margin of the iliac supraacetabular process with the dorsal margin of the iliac postacetabular process (corresponding with Prieto-Márquez.2009 character 252): (0) present; (1) absent.

297. Lateral profile of the dorsal margin of the ilium above the supraacetabular process (corresponding with Horner et al., 2004 character 100): (0) nearly straight or slightly convex; (1) strongly concave.

298. Morphological character of the pubic peduncle of the ilium (modified from Horner et al., 2004 character 92): (0) relatively long and stick-shaped in lateral view; (1) relatively short and triangular in lateral view.

299. Morphological character of the ischial peduncle of the ilium (modified from Godefroit et al., 2001 character 30): (0) formed by a single large knob; (1) composed of a relatively large knob and a relatively small knob separated by a shallow embayment; (2) composed of two knobs with similar size separated by a shallow embayment.

300. Ratio between the anteroposterior length of the postacetabular process and that of the central plate of the ilium (corresponding with Prieto-Márquez and Wagner, 2009 character 255): (0) up to 0.8; (1) greater than 0.8 but less than 1.1; (2) 1.1 or greater.

301. General profile of the postacetabular process of the ilium in lateral view (modified from Horner et al., 2004 character 93): (0) gradually tapering posteriorly, forming a wedge-shaped postacetabular process; (1) rectangular, with a straight or arched posterior margin.

302. Brevis shelf at the base of the postacetabular process of the ilium (modified from Godefroit et al., 2008 character 52): (0) present; (1) absent.

303. Mediolateral thickening of the posterior portion of the iliac postacetabular process (modified from Prieto-Márquez and Wagner, 2009 character 257): (0) present, the posterior portion of the postacetabular process moderately thickens mediolaterally on account of the mediolateral expansion of its ventral surface; (1) present, the posterior portion of the postacetabular process markedly thickens mediolaterally triggered by the dorsomedial twist of the postacetabular process; (2) absent, the posterior portion of the postacetabular process is

compressed mediolaterally.

304. Orientation of the dorsal margin of the postacetabular process relative to the ventral margin of the iliac acetabular region (modified from Prieto-Márquez and Wagner, 2009 character 260): (0) either almost horizontal or slightly directed posteroventrally; (1) directed posterodorsally.

305. Position of the sacral ridge on the medial surface of the iliac central plate (corresponding with Prieto-Márquez and Wagner, 2009 character 261): (0) located at 50%–70% the dorsoventral height of the iliac central plate; (1) located at 75%–80% the dorsoventral height of the iliac central plate.

306. Morphological character of the sacral ridge on the medial surface of the iliac central plate in medial view (modified from Prieto-Márquez and Wagner, 2009 character 262): (0) nearly straight and oriented anteroposteriorly; (1) arched and convex dorsally, with the anterior portion of the sacral ridge directed anteroventrally.

Pubis

307. Strong constriction of the posterior neck of the pubic prepubic process relative to the acetabular region (ratio between the dorsoventral depth of the posterior neck and the distance between the anterodorsal corner of the iliac peduncle and the anteriormost end of the ischial peduncle) (corresponding with Xing et al., 2014 character 307): (0) absent, with the ratio greater than 0.5; (1) present, with the ratio up to 0.5.

308. Orientation of the dorsoventral expansion in the anterior region of the pubic prepubic process (corresponding with Prieto-Márquez and Wagner, 2009 character 264): (0) the dorsal portion of the anterior blade of the prepubic process is more expanded than the ventral portion, so that the expansion of the anterior blade is directed dorsally; (1) the ventral portion of the anterior blade of the prepubic process is more expanded than the dorsal portion, so that the expansion of the anterior blade is directed ventrally.

309. Lateral profile of the anterior blade of the pubic prepubic process (modified from Prieto-Márquez and Wagner, 2009 character 265): (0) elliptic, anteroposteriorly longer than dorsoventrally tall, with the dorsal region slightly more expanded than the ventral region; (1) subovate, slightly deeper dorsoventrally than long anteroposteriorly, with the dorsal region much more expanded than the ventral region; (2) circular, with the ventral region much more expanded than the dorsal region; (3) subrectangular and anteroposteriorly elongate, with the ventral region

much more expanded than the dorsal region.

310. Dorsoventral depth of the anterior blade of the pubic prepubic process relative to that of the pubic acetabular margin (modified from Prieto-Márquez and Wagner, 2009 character 266): (0) the dorsoventral depth of the anterior blade is greater than that of the pubic acetabular margin; (1) the dorsoventral depth of the anterior blade is equal to or less than that of the pubic acetabular margin.

311. Anteroposterior length of the anterior blade of the prepubic process relative to that of the posterior neck (posterior constriction) of the prepubic process (modified from Horner et al., 2004 character 96): (0) the length of the anterior blade is less than that of the posterior neck; (1) the length of the anterior blade is equal to or greater than that of the posterior neck.

312. Morphological character of the iliac peduncle of the pubis (corresponding with Xing et al., 2012 character 300): (0) relatively short and robust; (1) relatively long and slender.

313. A sharp ridge on the lateral surface of the iliac and ischial peduncles, bounding the cranioventral margin of the acetabulum (corresponding with Prieto-Marquez and Wagner, 2009 character 269): (0) present; (1) absent or bearing a faint ridge.

314. A pubic obturator notch ventral to the ischial peduncle of the pubis for the passage of the obturator nerve (modified from Horner et al., 2004 character 97): (0) present; (1) absent.

315. Length/width ratio of the ischial peduncle of the pubis (modified from Prieto-Márquez and Wagner, 2009 character 271): (0) less than 2; (1) 2 to 3; (2) greater than 3.

316. Lateroventral protuberance in the proximal region of the ischial peduncle of the pubis (corresponding with Prieto-Márquez and Wagner, 2009 character 272): (0) absent or faintly developed; (1) present.

317. Ratio between the anteroposterior distance from the anterior margin of the prepubic process to the acetabular margin and the dorsoventral distance from the dorsal margin of the iliac peduncle to the ventral margin of the anterior end of the postpubic process (modified from Prieto-Márquez and Wagner, 2009 character 274): (0) less than 2.8; (1) 2.8 to 3.0; (2) greater than 3.0.

Ischium

318. Posterior curvature of the iliac peduncle of the ischium (corresponding with Prieto-Márquez and Wagner, 2009 character 275): (0) present, the ischial iliac peduncle is slightly curved posteriorly; (1) present, the ischial iliac peduncle is markedly curved posteriorly; (2) absent.

319. Angle between the anterodorsal margin and the anteroventral margin of the iliac peduncle of

the ischium (corresponding with Prieto-Márquez and Wagner, 2009 character 276): (0) up to 115º; (1) greater than 115º.

320. Ratio between the dorsoventral height of the iliac peduncle of the ischium and the length of the anterodorsal margin of this peduncle (corresponding with Prieto-Márquez and Wagner, 2009 character 277): (0) less than 1.5; (1) 1.5 to 2; (2) greater than 2.

321. Orientation of the anteroventral margin of the ischial iliac peduncle relative to the posterodorsal margin of this element (corresponding with Prieto-Márquez and Wagner, 2009 character 278): (0) the anteroventral margin is either parallel or convergent with the posterodorsal margin along the posteroventral direction; (1) the anteroventral margin is slightly divergent with the posterodorsal margin along the posteroventral direction.

322. Orientation of the anteroposterior axis of the ischial pubic peduncle relative to the ischial shaft in parallel with the level (modified from Prieto-Márquez and Wagner, 2009 character 279): (0) directed anteroventrally; (1) almost directed anteriorly.

323. Anteroposterior length of the ischial pubic peduncle relative to the dorsoventral depth of this element (corresponding with Prieto-Márquez and Wagner, 2009 character 280): (0) the anteroposterior length is greater than the dorsoventral depth; (1) the anteroposterior length is approximately equal to the dorsoventral depth; (2) the anteroposterior length is less than the dorsoventral depth.

324. A completely enclosed foramen surrounded by the obturator process and the pubic peduncle of the ischium in adults (corresponding with Xing et al., 2012 character 312): (0) absent; (1) present.

325. Location of the anterodorsal corner of the ischial pubic peduncle relative to the dorsal margin of the ischial shaft (corresponding with Prieto-Márquez and Wagner, 2009 character 281): (0) ventral to or at the same level as the dorsal margin of the ischial shaft; (1) dorsal to the dorsal margin of the ischial shaft.

326. Morphological character of the ischial shaft in lateral view (corresponding with Horner et al., 2004 character 98): (0) strongly curved downwards; (1) nearly straight.

327. Ratio between the dorsoventral depth of the ischial shaft at the midshaft and the anteroposteriorly length of the ischial shaft (corresponding with Prieto-Márquez and Wagner, 2009 character 282): (0) up to 0.05; (1) more than 0.05 and up to 0.075; (2) greater than 0.075.

328. Morphological character of the posterior end of the ischial shaft (modified from Godefroit et al., 2001 character 31): (0) strongly expanded ventrally, forming a large foot-shaped or boot-like protuberance at the posterior end of the ischium; (1) straight and gracile, without the pronounced ventral expansion at the posterior end of the ischium.

329. Orientation of the long axis of the ventral expansion of the ischial posterior end relative to the ischial shaft (corresponding with Prieto-Márquez and Wagner, 2009 character 286): (0) oriented ventrally; (1) oriented anteroventrally.

330. Strong anteroventral curvation of the ventral expansion of the ischial posterior end (corresponding with Prieto-Márquez and Wagner, 2009 character 285): (0) absent; (1) present.

Hindlimb and Pes

Femur

331. Slightly curved posteromedially distal half of the femoral shaft (modified from Norman, 2002 character 62): (0) present; (1) absent.

332. Development of the lesser trochanter on the anterolateral surface of the proximal portion of the femur (corresponding with Xing et al., 2012 character 320): (0) presence of a strongly developed lesser trochanter; (1) presence of a moderately developed lesser trochanter which is sometimes fused to the greater trochanter.

333. General profile of the posterior margin of the femoral fourth trochanter in lateral view (corresponding with Prieto-Márquez and Wagner, 2009 character 288): (0) triangular; (1) arcuate and smooth.

334. Morphological character of the anterior intercondylar groove in the distal region of the femur (modified from Norman, 2002 character 64): (0) fully open ventrally; (1) nearly or completely enclosed by the lateral and medial condyles owing to the fusion of these two condyles.

Tibia

335. Morphological character of the cnemial crest of the tibia (corresponding with Godefroit et al., 2000 character 31): (0) presence of an expanded anteriorly cnemial crest restricted to the proximal end of the tibia; (1) presence of an expanded ventrally cnemial crest along the proximal half of the tibial shaft.

Fibula

336. General shape of the distal end of the fibula in lateral view (modified from Godefroit et al.,

2000 character 32): (0) subtriangular, forming a moderately expanded anteriorly fibular distal end; (1) club-shaped, forming a greatly expanded anteriorly fibular distal end.

Tarsals

337. General shape of the anterior ascending process of the astragalus in anterior view (corresponding with Godefroit et al., 2000 character 33): (0) subtriangular in shape and skewed laterally; (1) triangular in shape and equilateral for each margin of the anterior ascending process.

338. Development of the articular surface of the astragalus for the internal malleolus of the tibia (corresponding with Prieto-Márquez and Wagner, 2009 character 291): (0) markedly expanded medially, articulating with the whole ventral surface of the tibial internal malleolus; (1) moderately expanded medially, articulating with only part of the ventral surface of the tibial internal malleolus.

339. Distal tarsals II and III (corresponding with Horner et al., 2004 character 102): (0) present; (1) absent.

Metatarsals

340. Metatarsal I (modified from Norman, 2002 character 66): (0) present, slender and rod-shaped; (1) absent.

341. Ratio between the proximodistal length of the metatarsal III and the mediolateral width of this element at the midshaft (modified from Prieto-Márquez and Wagner, 2009 character 294): (0) greater than 4.5; (1) up to 4.5.

Pedal Phalanges

342. Proximodistal length of pedal phalanx II-2 relative to the mediolateral width of this element at the midshaft (modified from Prieto-Márquez and Wagner, 2009 character 295): (0) the proximodistal length is slightly less than the mediolateral width at the midshaft; (1) the mediolateral width at the midshaft is approximately twice as long as the proximodistal length.

343. Ratio between the mediolateral width at the midshaft and the proximodistal length of pedal phalanges III-2 and III-3 (modified from Horner et al., 2004 character 104): (0) up to 3; (1) greater than 3.

344. Ratio between the mediolateral width at the midshaft and the proximodistal length of pedal phalanges IV-2, IV-3 and IV-4 (modified from Prieto-Márquez and Wagner, 2009 character 297): (0) up to 3; (1) greater than 3.

345. General shape of pedal unguals in dorsal view (modified from Norman, 2002 character 67): (0) claw-shaped, with the presence of prominent claw grooves; (1) hoof-shaped, with the presence of faint claw grooves or absence of the claw grooves.

346. Plantar median ridge on the ventral surface of pedal ungual (corresponding with Godefroit et al., 2008 character 56): (0) absent; (1) present.

References

Brett-Surman MK, Wagner JR (2007) Discussion of character analysis of the appendicular anatomy in Campanian and Maastrichtian North American hadrosaurids – variation and ontogeny. In: Carpenter K ed. Horns and Beaks: Ceratopsian and Ornithopod Dinosaurs. Bloomington: Indiana University Press. pp. 135–169.

Evans DC (2006) Nasal cavity homologies and cranial crest function in lambeosaurine dinosaurs. Paleobiology 32: 109–125.

Evans DC, Reisz RR (2007) Anatomy and relationships of *Lambeosaurus magnicristatus*, a crested hadrosaurid dinosaur (Ornithischia) from the Dinosaur Park Formation, Alberta. Journal of Vertebrate Paleontology 27: 373–393.

Gates TA, Sampson SD (2007) A new species of *Gryposaurus* (Dinosauria: Hadrosauridae) from the late Campanian Kaiparowits Formation, southern Utah, USA. Zoological Journal of the Linnean Society 151: 351–376.

Godefroit P, Zan SQ, Jin LY (2000) *Charonosaurus jiayinensis* n.g., n.sp., a lambeosaurine dinosaur from the Late Cretaceous of northeastern China. Comptes Rendus de l'Academie des Sciences de Paris, Sciences de la Terre et des Planetes 330: 875–882.

Godefroit P, Zan SQ, Jin LY (2001) The Maastrichtian (Late Cretaceous) lambeosaurine dinosaur *Charonosaurus jiayinensis* from north-eastern China. Bulletin de l'Institut Royal des Sciences Naturelles du Belgique, Sciences de la Terre 71: 119–168.

Godefroit P, Bolotsky YL, Van Itterbeeck J (2004) The lambeosaurine dinosaur *Amurosaurus riabinini*, from the Maastrichtian of Far Eastern Russia. Acta Palaeontologica Polonica 49: 585–618.

Godefroit P, Hai SL, Yu TX, Lauters P (2008) New hadrosaurid dinosaurs from the uppermost

Cretaceous of northeastern China. Acta Palaeontologica Polonica 53: 47–74.

Horner JR, Weishampel DB, Forster CA (2004) Hadrosauridae. In: Weishampel DB, Dodson P, Osmólska H, eds. The Dinosauria, second edition. Berkeley: University of California Press. pp. 438–463.

Kobayashi Y, Azuma. Y (1999) Cranial material of a new iguanodontian dinosaur from the Lower Cretaceous Kitadani Formation of Japan. Journal of Vertebrate Paleontology 19: (3, suppl.). 57A.

Norman DB (2002) On Asian ornithopods (Dinosauria: Ornithischia). 4. *Probactrosaurus* Rozhdestvensky, 1966. Zoological Journal of the Linnean Society 136: 113–144.

Prieto-Márquez A, Wagner JR (2009) *Pararhabdodon isonensis* and *Tsintaosurus spinorhinus*: a new clade of lambeosaurine hadrosaurids from Eurasia. Cretaceous Research 30: 1238–1246.

Prieto-Márquez A (2010) *Glishades ericksoni*, a new hadrosauroid (Dinosauria: Ornithopoda) from the Late Cretaceous of North America. Zootaxa 2452: 1–17.

Sereno PC (1986) Phylogeny of the bird-hipped dinosaurs (Order Ornithischia). National Geographic Research 2: 234–256.

Suzuki D, Weishampel DB, Minoura N (2004) *Nipponosaurus sachalinensis* (Dinosauria: Ornithopoda): anatomy and systematic position within Hadrosauridae. Journal of Vertebrate Paleontology 24: 145–164.

Wagner JR (2001) The hadrosaurian dinosaurs (Ornithischia: Hadrosauria) of Big Bend tional Park, Brewster County, Texas, with implications for Late Cretaceous Paleozoogeography. Unpubished Master's Thesis. Austin: Texas Tech University. pp. 1–417.

Wang RF, You HL, Wang SZ, Xu SC, Yi J, Xie LJ, et al. (2017) A second hadrosauroid dinosaur from the early Late Cretaceous of Zuoyun, Shanxi Province, China. Historical Biology 29(1): 17–24.

Weishampel DB (1981) The nasal cavity of lambeosaurine hadrosaurids (Reptilia: Ornithischia): comparative anatomy and homologies. Journal of Paleontology 55: 1046–1057.

Weishampel DB, Horner JR (1986) The hadrosaurid dinosaurs of the Iren Dabasu Formation (People's Republic of China, Late Cretaceous). Journal of Vertebrate Paleontology 6: 38–45.

Weishampel DB, Norman DB, Grigorescu D (1993) *Telmatosaurus transsylvanicus* from the Late Cretaceous of Romania: the most basal hadrosaurid. Palaeontology 36(2): 361–385.

Xing H, Prieto-Márquez A, Gu W, Yu TX (2012) Re-evaluation and phylogenetic analysis of *Wulagasaurus dongi*, a hadrosaurine dinosaur from the Maastrichtian of northeast China. Vertebrata PalAsiatica 50(2): 160–169.

Xing H, Wang DY, Han FL, Sullivan C, Ma QY, He YM, et al. (2014) A new basal hadrosauroid dinosaur (Dinosauria: Ornithopoda) with transitional features from the Late Cretaceous of Henan Province, China. PLoS ONE 9(6): e98821.

You HL, Luo ZX, Shubin NH, Witmer LM, Tang ZL, Tang F, et al. (2003) The earliest-known duck-billed dinosaur from deposits of late Early Cretaceous age in northwest China and hadrosaur evolution. Cretaceous Research 24: 347–355.
