# Supplementary material for: Supplementary cranial description of the types of Edmontosaurus regalis (Ornithischia: Hadrosauridae), with comments on the phylogenetics and biogeography of Hadrosaurinae
Source: PLoS One. 2017 Apr 6;12(4):e0175253. doi: 10.1371/journal.pone.0175253 (PMC5383305; doi:10.1371/journal.pone.0175253)
Supplement: S2 Text — (PDF) [file pone.0175253.s005.pdf]

S2 Text

Probability calculation of ancestral areas for nodes within Hadrosaurinae

Abbreviations: a, Asia; e, Europe; n, North America; na, not applicable; P, probabilities; s, South America.

Node 1

P (*Hadrosaurus foulkii*) × P (node 2) × P (node 3) (polytomy)

Condition 1: [P (*Hadrosaurus foulkii*) × P (node 2)] × P (node 3)

Step C11: [1/1 n × (1/3 a, 2/3 n)] × (7/38 a, 28/38 n, 3/38 s)

Step C12: (1/3 an, 2/3 n^2) × (7/38 a, 28/38 n, 3/38 s)

Step C13: (1/6 a, 1/6 n + 2/3 n) × (7/38 a, 28/38 n, 3/38 s)

Step C14: (1/6 a, 5/6 n) × (7/38 a, 28/38 n, 3/38 s)

Step C15: 7/228 a^2, 28/228 an, 3/228 as (na), 35/228 an, 140/228 n^2, 15/228 ns

Step C16: 7/228 a + 28/456 a + 35/456 a, 28/456 n + 35/456 n + 140/228 n + 15/456 n, 15/456 s

Step C17: 77/456 a, 358/456 n, 15/456 s

Step C18: 77/450 a, 358/450 n, 15/450 s ≈ a, 17%; n, 80%; s, 3%.

Condition 2: [P (*Hadrosaurus foulkii*) × P (node 3)] × P (node 2)

Step C21: [1/1 n × (7/38 a, 28/38 n, 3/38 s)] × (1/3 a, 2/3 n)

Step C22: (7/38 an, 28/38 n^2, 3/38 ns) × (1/3 a, 2/3 n)

Step C23: (7/76 a, 7/76 n + 28/38 n + 3/76 n, 3/76 s) × (1/3 a, 2/3 n)

Step C24: (7/76 a, 66/76 n, 3/76 s) × (1/3 a, 2/3 n)

Step C25: 7/228 a^2, 66/228 an, 3/228 as (na), 14/228 an, 132/228 n^2, 6/228 ns

Step C26: 7/228 a + 66/456 a + 14/456 a, 66/456 n + 14/456 n + 132/228 n + 6/456 n, 6/456 s

Step C27: 94/456 a, 350/456 n, 6/456 s

Step C28: 94/450 a, 350/450 n, 6/450 s ≈ a, 21%; n, 78%; s, 1%.

Condition 3: [P (node 2) × P (node 3)] × P (*Hadrosaurus foulkii*)

Step C31: [(1/3 a, 2/3 n) × (7/38 a, 28/38 n, 3/38 s)] × 1/1 n

Step C32: [7/114 a^2, 28/114 an, 3/114 as (na), 14/114 an, 56/114 n^2, 6/114 ns] × 1/1 n

Step C33: (7/114 a + 28/228 a + 14/228 a, 28/228 n + 14/228 n + 56/114 n + 6/228 n, 6/228 s) × 1/1 n

Step C34: (56/228 a, 160/228 n, 6/228 s) × 1/1 n

Step C35: (56/222 a, 160/222 n, 6/222 s) × 1/1 n

Step C36: 56/222 an, 160/222 n^2, 6/222 ns

Step C37: 56/444 a, 56/444 n + 160/222 n + 6/444 n, 6/444 s

Step C38: 56/444 a, 382/444 n, 6/444 s ≈ a, 13%; n, 86%; s, 1%.

P (condition 1) + P (condition 2) + P (condition 3)

Step 1: (77/450 a, 358/450 n, 15/450 s) + (94/450 a, 350/450 n, 6/450 s) + (56/444 a, 382/444 n, 6/444 s)

Step 2: 77/450 a + 94/450 a + 56/444 a, 358/450 n + 350/450 n + 382/444 n, 15/450 s + 6/450 s + 6/444 s

Step 3: 101124/199800 a, 486252/199800 n, 12024/199800 s

Step 4: 101124/599400 a, 486252/599400 n, 12024/599400 s ≈ **a, 17%; n, 81%; s, 2%**.

Node 2

P (*Wulagasaurus dongi*) × P (*Acristavus gagslarsoni*) × P (node 4) (polytomy)

Condition 1: [P (*Wulagasaurus dongi*) × P (*Acristavus gagslarsoni*)] × P (node 4)

Step C11: (1/1 a × 1/1 n) × 1/1 n

Step C12: 1 an × 1/1 n

Step C13: (1/2 a, 1/2 n) × 1/1 n

Step C14: 1/2 an, 1/2 n^2

Step C15: 1/4 a, 1/4 n + 1/2 n

Step C16: 1/4 a, 3/4 n = a, 25%; n, 75%.

Condition 2: [P (*Wulagasaurus dongi*) × P (node 4)] × P (*Acristavus gagslarsoni*)

Step C21: (1/1 a × 1/1 n) × 1/1 n

Step C22: 1 an × 1/1 n

Step C23: (1/2 a, 1/2 n) × 1/1 n

Step C24: 1/2 an, 1/2 n^2

Step C25: 1/4 a, 1/4 n + 1/2 n

Step C26: 1/4 a, 3/4 n = a, 25%; n, 75%.

Condition 3: [P (*Acristavus gagslarsoni*) × P (node 4)] × P (*Wulagasaurus dongi*)

Step C31: (1/1 n × 1/1 n) × 1/1 a

Step C32: 1 n^2 × 1/1 a

Step C33: 1/1 n × 1/1 a

Step C34: 1 an

Step C35: 1/2 a, 1/2 n = a, 50%; n, 50%.

P (condition 1) + P (condition 2) + P (condition 3)

Step 1: (1/4 a, 3/4 n) + (1/4 a, 3/4 n) + (1/2 a, 1/2 n)

Step 2: 1/4 a + 1/4 a + 1/2 a, 3/4 n + 3/4 n + 1/2 n

Step 3: 4/4 a, 8/4 n

Step 4: 4/12 a, 8/12 n ≈ **a, 33%; n, 67%**.

Node 3

P (node 6) × P (node 7)

Step 1: (3/4 n, 1/4 s) × (7/16 a, 9/16 n)

Step 2: 21/64 an, 27/64 n^2, 7/64 as (na), 9/64 ns

Step 3: 21/128 a, 21/128 n + 27/64 n + 9/128 n, 9/128 s

Step 4: 21/128 a, 84/128 n, 9/128 s

Step 5: 21/114 a, 84/114 n, 9/114 s ≈ **a, 18%; n, 74%; s, 8%**.

Node 4

P (*Maiasaura peeblesorum*) × P (node 5)

Step 1: 1/1 n × 1/1 n

Step 2: 1 n^2

Step 3: 1 n = **n, 100%**.

Node 5

P (*Brachylophosaurus canadensis*) × P (*Probrachylophosaurus bergei*)

Step 1: 1/1 n × 1/1 n

Step 2: 1 n^2

Step 3: 1 n = **n, 100%**.

Node 6

P (*Kritosaurus navajovius*) × P (node 8)

Step 1: 1/1 n × (1/2 n, 1/2 s)

Step 2: 1/2 n^2, 1/2 ns

Step 3: 1/2 n + 1/4 n, 1/4 s

Step 4: 3/4 n, 1/4 s = **n, 75%; s, 25%**.

Node 7

P (node 12) × P (node 15)

Step 1: (3/4 a, 1/4 n) × (1/8 a, 7/8 n)

Step 2: 3/32 a^2, 21/32 an, 1/32 an, 7/32 n^2

Step 3: 3/32 a + 21/64 a + 1/64 a, 21/64 n + 1/64 n + 7/32 n

Step 4: 28/64 a, 36/64 n ≈ **a, 44%; n, 56%**.

Node 8

P (*Secernosaurus koerneri*) × P (node 9)

Step 1: 1/1 s × 1/1 n

Step 2: 1 ns

Step 3: 1/2 n, 1/2 s = **n, 50%; s, 50%**.

Node 9

P (node 10) × P (node 11)

Step 1: 1/1 n × 1/1 n

Step 2: 1 n^2

Step 3: 1 n = **n, 100%**.

Node 10

P (*Gryposaurus latidens*) × P (*Rhinorex condrupus*)

Step 1: 1/1 n × 1/1 n

Step 2: 1 n^2

Step 3: 1 n = **n, 100%**.

Node 11

P (*Gryposaurus monumentensis*) × P (*Gryposaurus notabilis*)

Step 1: 1/1 n × 1/1 n

Step 2: 1 n^2

Step 3: 1 n = **n, 100%**.

Node 12

P (*Kerberosaurus manakini*) × P (node 13)

Step 1: 1/1 a × (1/2 a, 1/2 n)

Step 2: 1/2 a^2, 1/2 an

Step 3: 1/2 a + 1/4 a, 1/4 n

Step 4: 3/4 a, 1/4 n = **a, 75%; n, 25%**.

Node 13

P (*Shantungosaurus giganteus*) × P (node 14)

Step 1: 1/1 a × 1/1 n

Step 2: 1 an

Step 3: 1/2 a, 1/2 n = **a, 50%; n, 50%**.

Node 14

P (*Edmontosaurus regalis*) × P (*Edmontosaurus annectens*)

Step 1: 1/1 n × 1/1 n

Step 2: 1 n^2

Step 3: 1 n = **n, 100%**.

Node 15

P (*Lophorhothon atopus*) × P (node 16)

Step 1: 1/1 n × (1/4 a, 3/4 n)

Step 2: 1/4 an, 3/4 n^2

Step 3: 1/8 a, 1/8 n + 3/4 n

Step 4: 1/8 a, 7/8 n ≈ **a, 13%; n, 87%**.

Node 16

P (*Prosaurolophus maximus*) × P (node 17)

Step 1: 1/1 n × (1/2 a, 1/2 n)

Step 2: 1/2 an, 1/2 n^2

Step 3: 1/4 a, 1/4 n + 1/2 n

Step 4: 1/4 a, 3/4 n = **a, 25%; n, 75%**.

Node 17

P (*Saurolophus angustirostris*) × P (*Saurolophus osborni*)

Step 1: 1/1 a × 1/1 n

Step 2: 1 an

Step 3: 1/2 a, 1/2 n = **a, 50%; n, 50%**.
